# Supplementary figures and images for: Molecular structures enumeration and virtual screening in the chemical space with RetroPath2.0 (part 1 of 2)
Source: J Cheminform. 2017 Dec 19;9:64. doi: 10.1186/s13321-017-0252-9 (PMC5736515; doi:10.1186/s13321-017-0252-9)

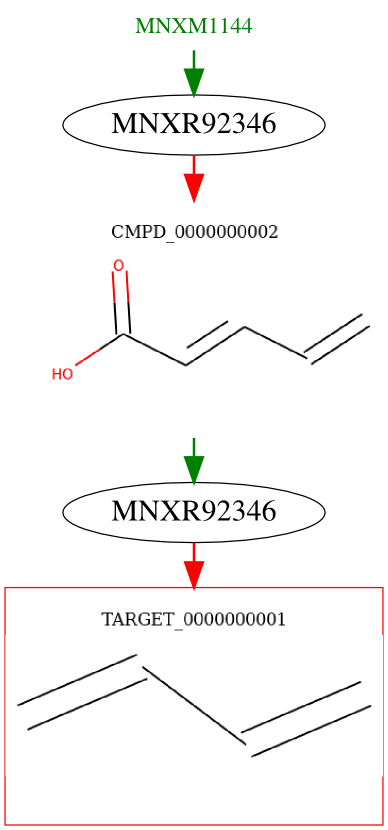

Supplement: Supplementary file 1 — Additional file 1. Monomers maps obtained running Retropath2.0 in section “Virtual screening in the chemical space”. The 17 compounds of the 158 available monomers that can be naturally synthesized and the corresponding synthesis pathways. [file 13321_2017_252_MOESM1_ESM.zip › maps-monomers/1,4-butadiene/path1.png]

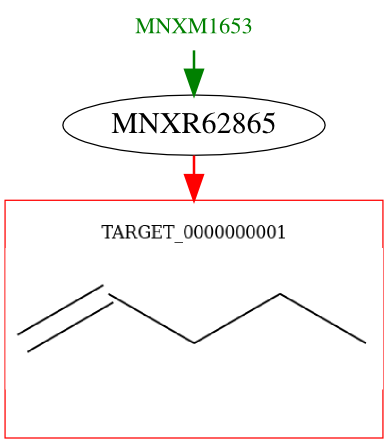

Supplement: Supplementary file 1 — Additional file 1. Monomers maps obtained running Retropath2.0 in section “Virtual screening in the chemical space”. The 17 compounds of the 158 available monomers that can be naturally synthesized and the corresponding synthesis pathways. [file 13321_2017_252_MOESM1_ESM.zip › maps-monomers/1-pentene/path1.png]

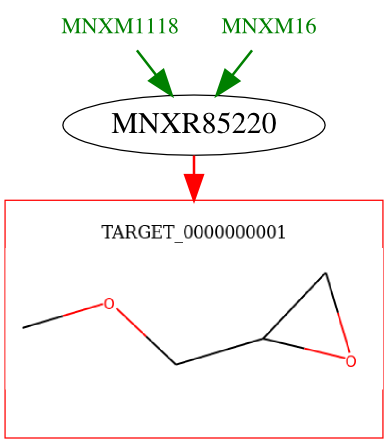

Supplement: Supplementary file 1 — Additional file 1. Monomers maps obtained running Retropath2.0 in section “Virtual screening in the chemical space”. The 17 compounds of the 158 available monomers that can be naturally synthesized and the corresponding synthesis pathways. [file 13321_2017_252_MOESM1_ESM.zip › maps-monomers/3-methoxypropylene_oxide/path1.png]

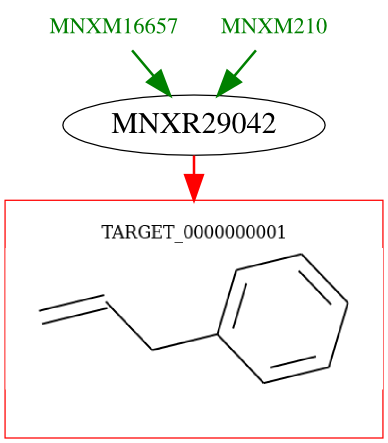

Supplement: Supplementary file 1 — Additional file 1. Monomers maps obtained running Retropath2.0 in section “Virtual screening in the chemical space”. The 17 compounds of the 158 available monomers that can be naturally synthesized and the corresponding synthesis pathways. [file 13321_2017_252_MOESM1_ESM.zip › maps-monomers/3-phenyl-1-propene/path1.png]

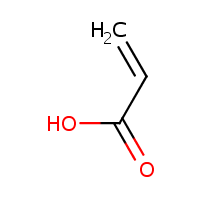

Supplement: Supplementary file 1 — Additional file 1. Monomers maps obtained running Retropath2.0 in section “Virtual screening in the chemical space”. The 17 compounds of the 158 available monomers that can be naturally synthesized and the corresponding synthesis pathways. [file 13321_2017_252_MOESM1_ESM.zip › maps-monomers/acrylic_acid/path1.png]

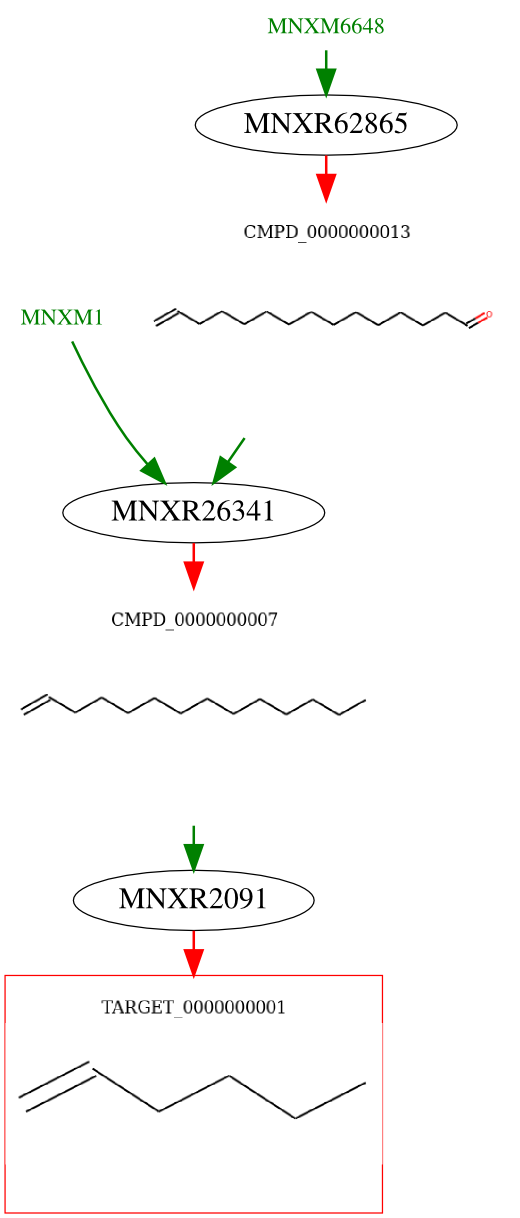

Supplement: Supplementary file 1 — Additional file 1. Monomers maps obtained running Retropath2.0 in section “Virtual screening in the chemical space”. The 17 compounds of the 158 available monomers that can be naturally synthesized and the corresponding synthesis pathways. [file 13321_2017_252_MOESM1_ESM.zip › maps-monomers/butylethylene/path1.png]

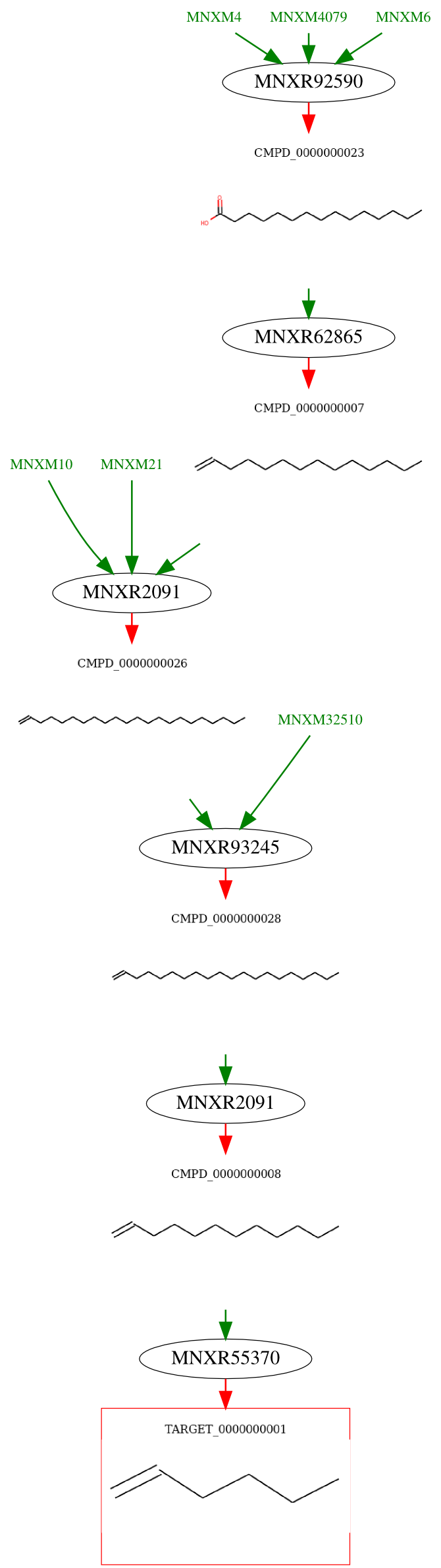

Supplement: Supplementary file 1 — Additional file 1. Monomers maps obtained running Retropath2.0 in section “Virtual screening in the chemical space”. The 17 compounds of the 158 available monomers that can be naturally synthesized and the corresponding synthesis pathways. [file 13321_2017_252_MOESM1_ESM.zip › maps-monomers/butylethylene/path10.png]

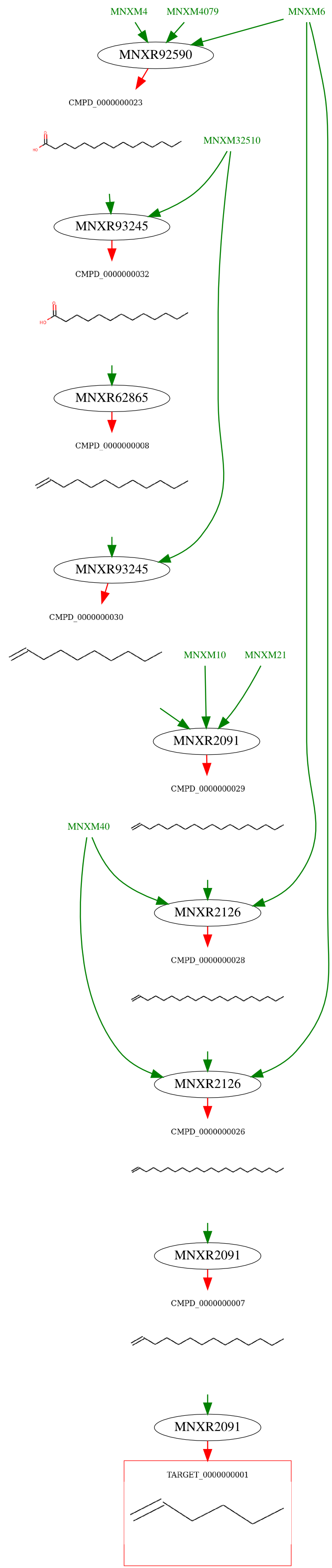

Supplement: Supplementary file 1 — Additional file 1. Monomers maps obtained running Retropath2.0 in section “Virtual screening in the chemical space”. The 17 compounds of the 158 available monomers that can be naturally synthesized and the corresponding synthesis pathways. [file 13321_2017_252_MOESM1_ESM.zip › maps-monomers/butylethylene/path100.png]

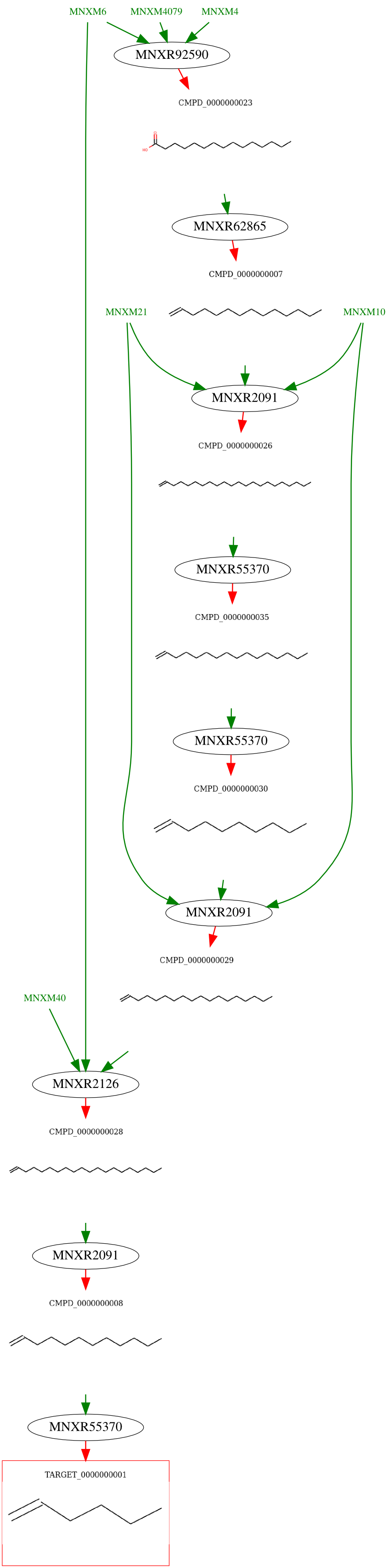

Supplement: Supplementary file 1 — Additional file 1. Monomers maps obtained running Retropath2.0 in section “Virtual screening in the chemical space”. The 17 compounds of the 158 available monomers that can be naturally synthesized and the corresponding synthesis pathways. [file 13321_2017_252_MOESM1_ESM.zip › maps-monomers/butylethylene/path101.png]

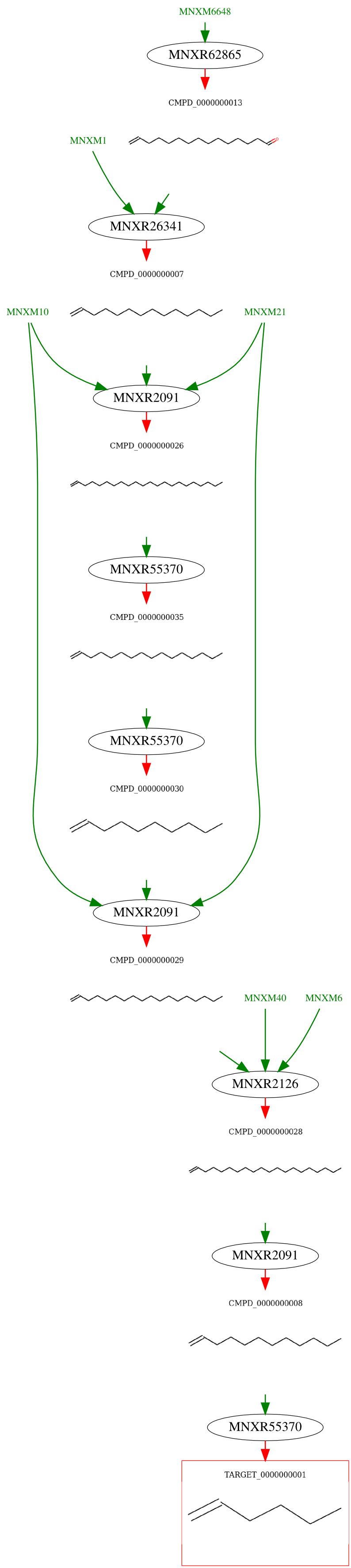

Supplement: Supplementary file 1 — Additional file 1. Monomers maps obtained running Retropath2.0 in section “Virtual screening in the chemical space”. The 17 compounds of the 158 available monomers that can be naturally synthesized and the corresponding synthesis pathways. [file 13321_2017_252_MOESM1_ESM.zip › maps-monomers/butylethylene/path102.png]

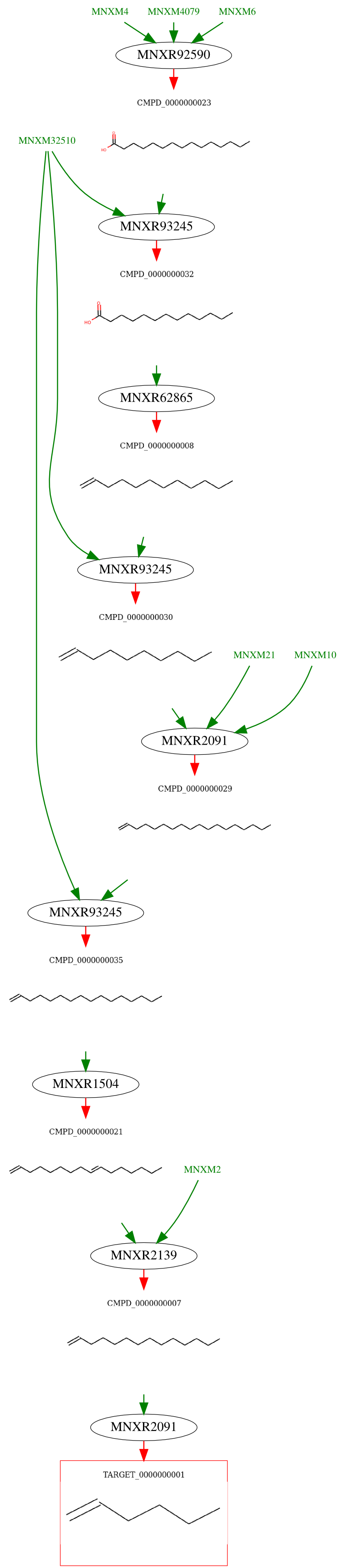

Supplement: Supplementary file 1 — Additional file 1. Monomers maps obtained running Retropath2.0 in section “Virtual screening in the chemical space”. The 17 compounds of the 158 available monomers that can be naturally synthesized and the corresponding synthesis pathways. [file 13321_2017_252_MOESM1_ESM.zip › maps-monomers/butylethylene/path103.png]

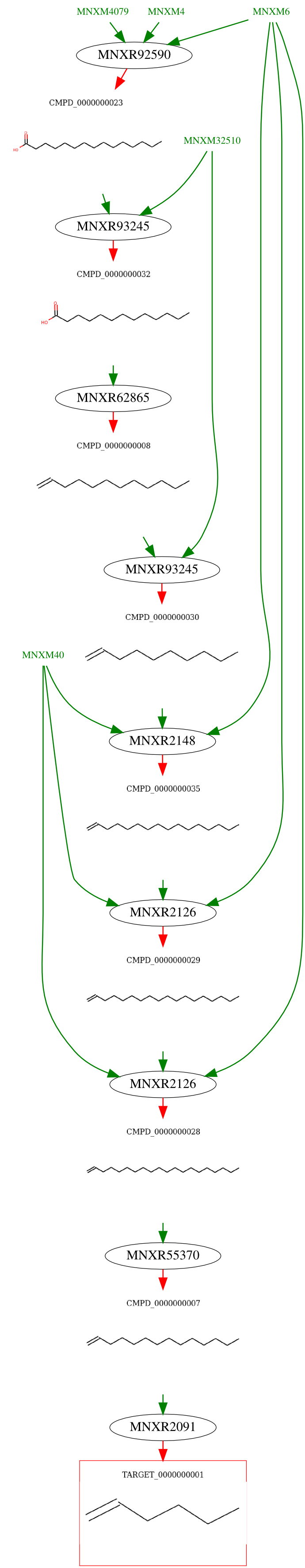

Supplement: Supplementary file 1 — Additional file 1. Monomers maps obtained running Retropath2.0 in section “Virtual screening in the chemical space”. The 17 compounds of the 158 available monomers that can be naturally synthesized and the corresponding synthesis pathways. [file 13321_2017_252_MOESM1_ESM.zip › maps-monomers/butylethylene/path104.png]

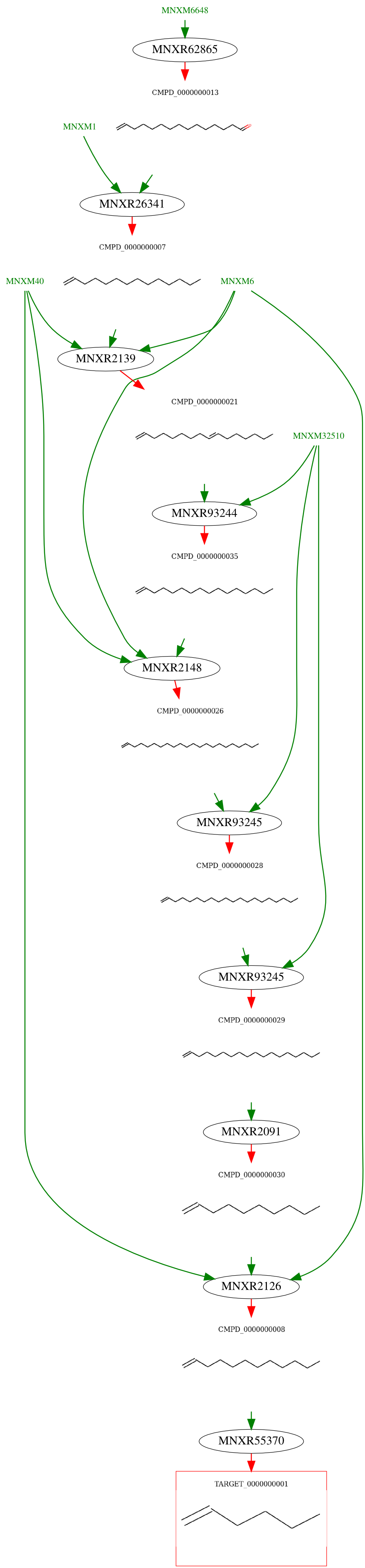

Supplement: Supplementary file 1 — Additional file 1. Monomers maps obtained running Retropath2.0 in section “Virtual screening in the chemical space”. The 17 compounds of the 158 available monomers that can be naturally synthesized and the corresponding synthesis pathways. [file 13321_2017_252_MOESM1_ESM.zip › maps-monomers/butylethylene/path105.png]

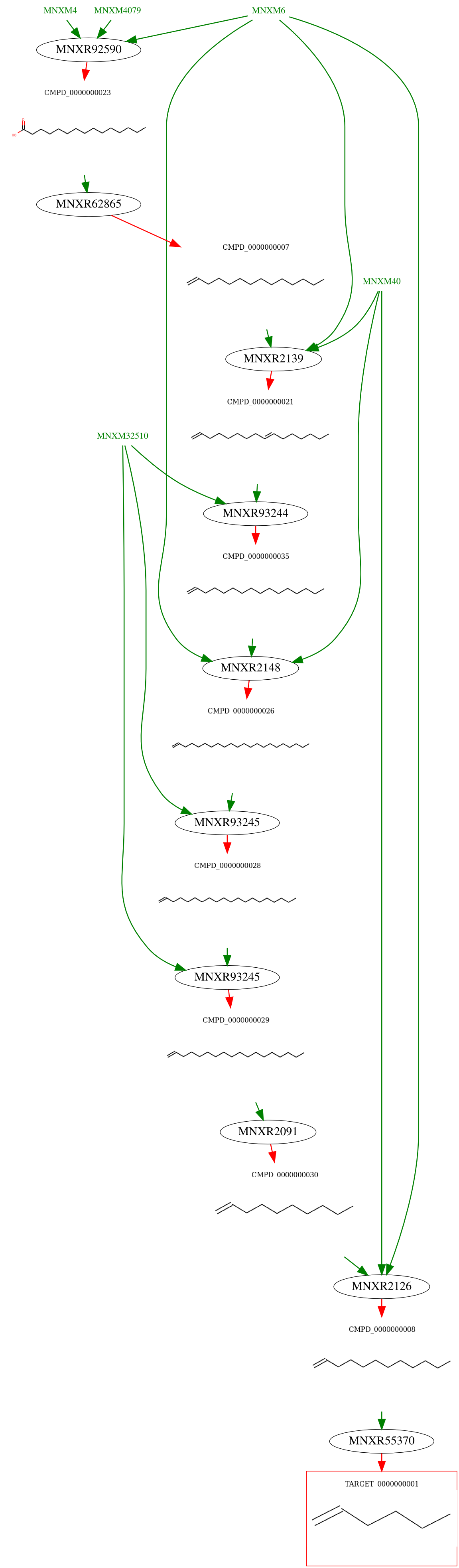

Supplement: Supplementary file 1 — Additional file 1. Monomers maps obtained running Retropath2.0 in section “Virtual screening in the chemical space”. The 17 compounds of the 158 available monomers that can be naturally synthesized and the corresponding synthesis pathways. [file 13321_2017_252_MOESM1_ESM.zip › maps-monomers/butylethylene/path106.png]

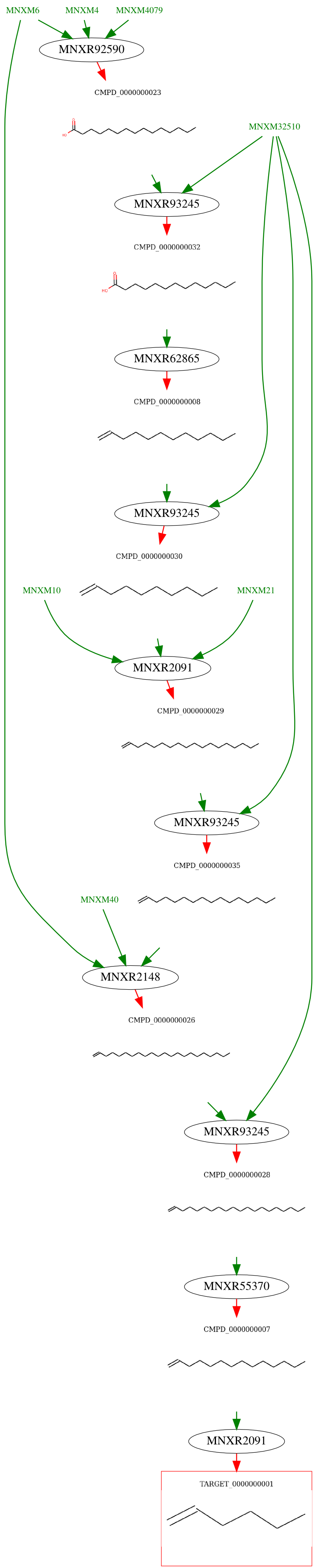

Supplement: Supplementary file 1 — Additional file 1. Monomers maps obtained running Retropath2.0 in section “Virtual screening in the chemical space”. The 17 compounds of the 158 available monomers that can be naturally synthesized and the corresponding synthesis pathways. [file 13321_2017_252_MOESM1_ESM.zip › maps-monomers/butylethylene/path107.png]

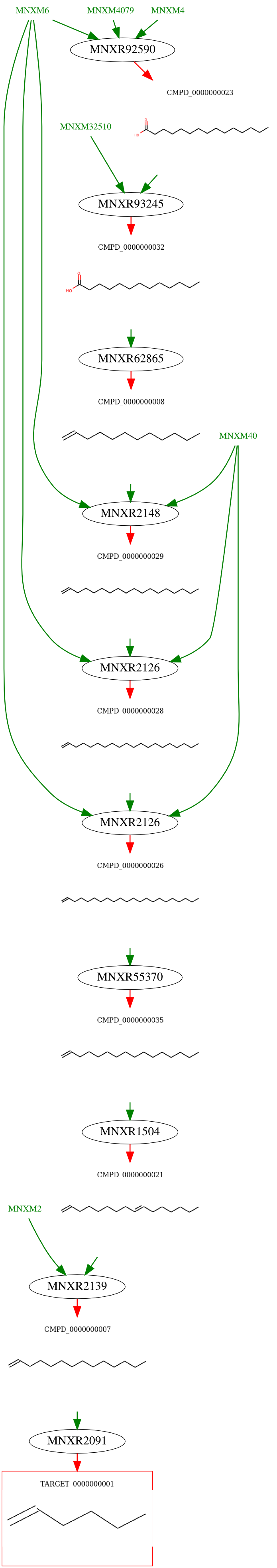

Supplement: Supplementary file 1 — Additional file 1. Monomers maps obtained running Retropath2.0 in section “Virtual screening in the chemical space”. The 17 compounds of the 158 available monomers that can be naturally synthesized and the corresponding synthesis pathways. [file 13321_2017_252_MOESM1_ESM.zip › maps-monomers/butylethylene/path108.png]

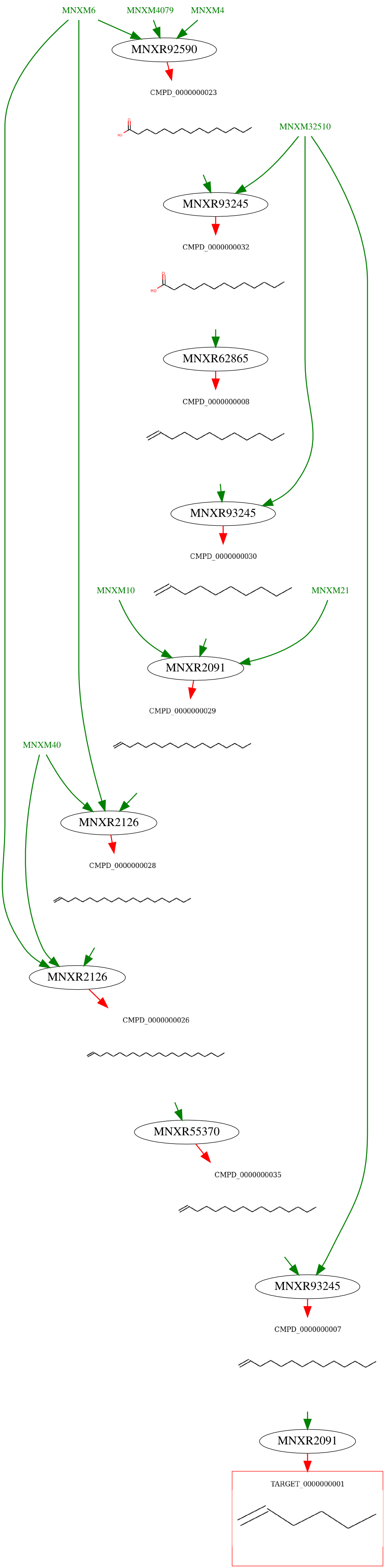

Supplement: Supplementary file 1 — Additional file 1. Monomers maps obtained running Retropath2.0 in section “Virtual screening in the chemical space”. The 17 compounds of the 158 available monomers that can be naturally synthesized and the corresponding synthesis pathways. [file 13321_2017_252_MOESM1_ESM.zip › maps-monomers/butylethylene/path109.png]

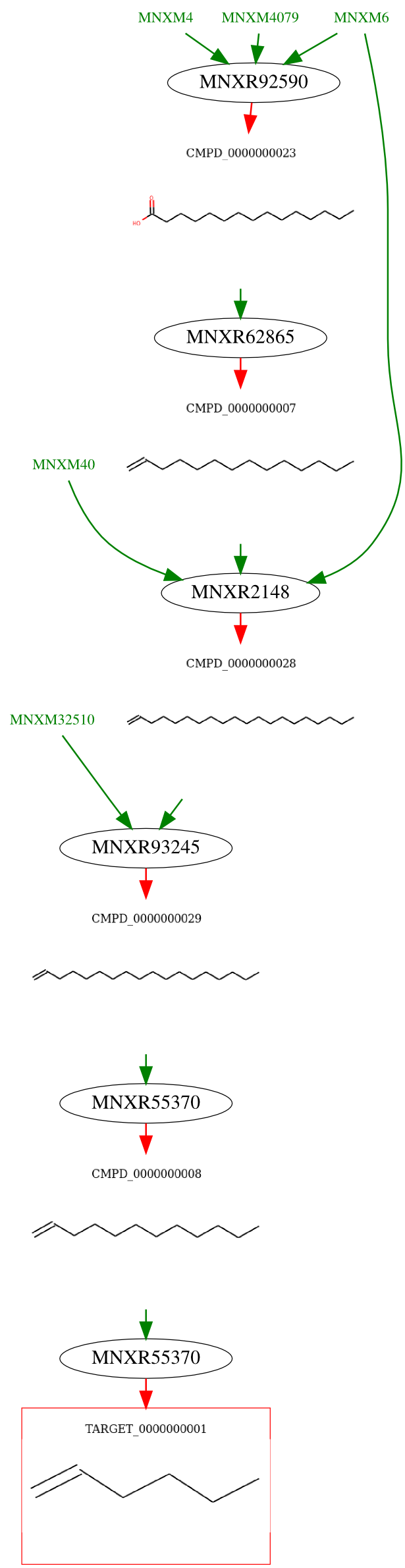

Supplement: Supplementary file 1 — Additional file 1. Monomers maps obtained running Retropath2.0 in section “Virtual screening in the chemical space”. The 17 compounds of the 158 available monomers that can be naturally synthesized and the corresponding synthesis pathways. [file 13321_2017_252_MOESM1_ESM.zip › maps-monomers/butylethylene/path11.png]

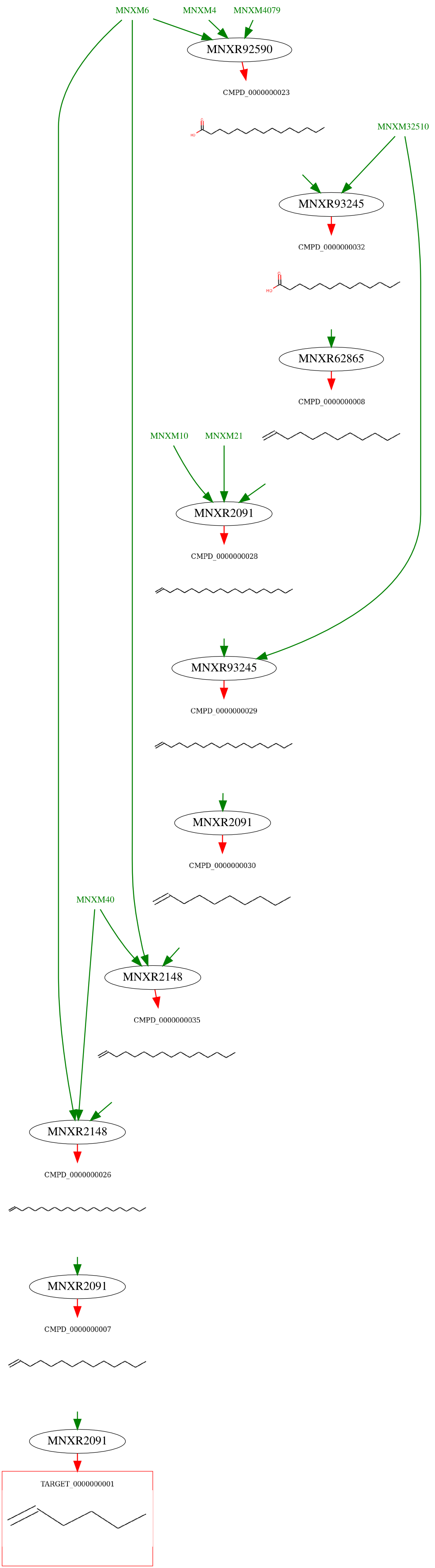

Supplement: Supplementary file 1 — Additional file 1. Monomers maps obtained running Retropath2.0 in section “Virtual screening in the chemical space”. The 17 compounds of the 158 available monomers that can be naturally synthesized and the corresponding synthesis pathways. [file 13321_2017_252_MOESM1_ESM.zip › maps-monomers/butylethylene/path110.png]

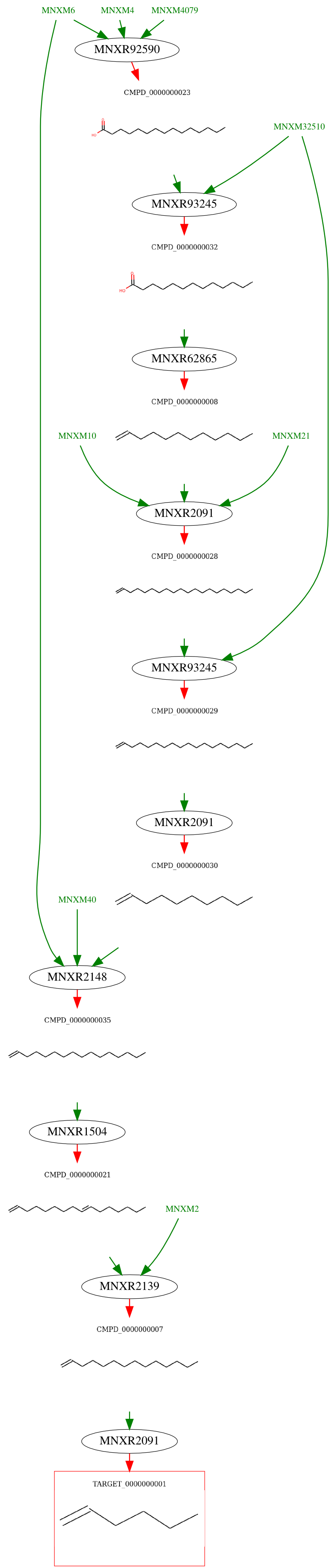

Supplement: Supplementary file 1 — Additional file 1. Monomers maps obtained running Retropath2.0 in section “Virtual screening in the chemical space”. The 17 compounds of the 158 available monomers that can be naturally synthesized and the corresponding synthesis pathways. [file 13321_2017_252_MOESM1_ESM.zip › maps-monomers/butylethylene/path111.png]

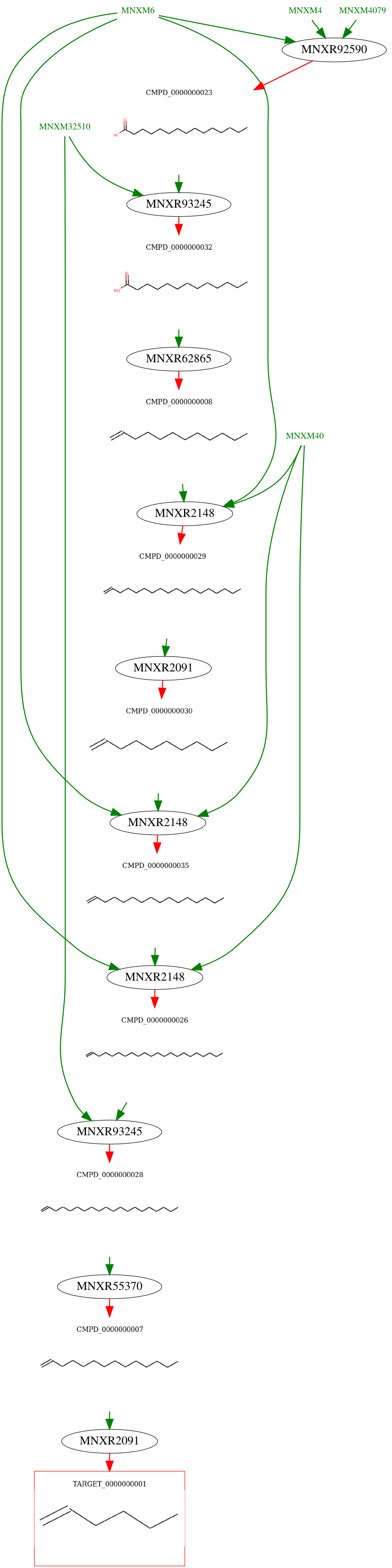

Supplement: Supplementary file 1 — Additional file 1. Monomers maps obtained running Retropath2.0 in section “Virtual screening in the chemical space”. The 17 compounds of the 158 available monomers that can be naturally synthesized and the corresponding synthesis pathways. [file 13321_2017_252_MOESM1_ESM.zip › maps-monomers/butylethylene/path112.png]

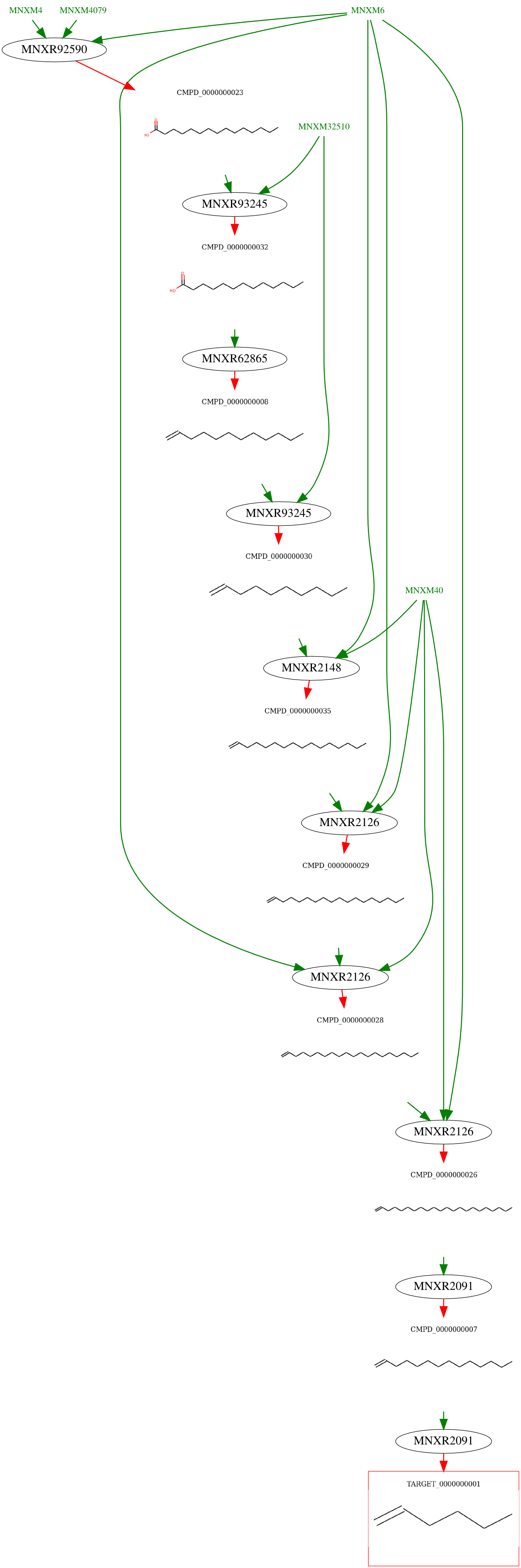

Supplement: Supplementary file 1 — Additional file 1. Monomers maps obtained running Retropath2.0 in section “Virtual screening in the chemical space”. The 17 compounds of the 158 available monomers that can be naturally synthesized and the corresponding synthesis pathways. [file 13321_2017_252_MOESM1_ESM.zip › maps-monomers/butylethylene/path113.png]

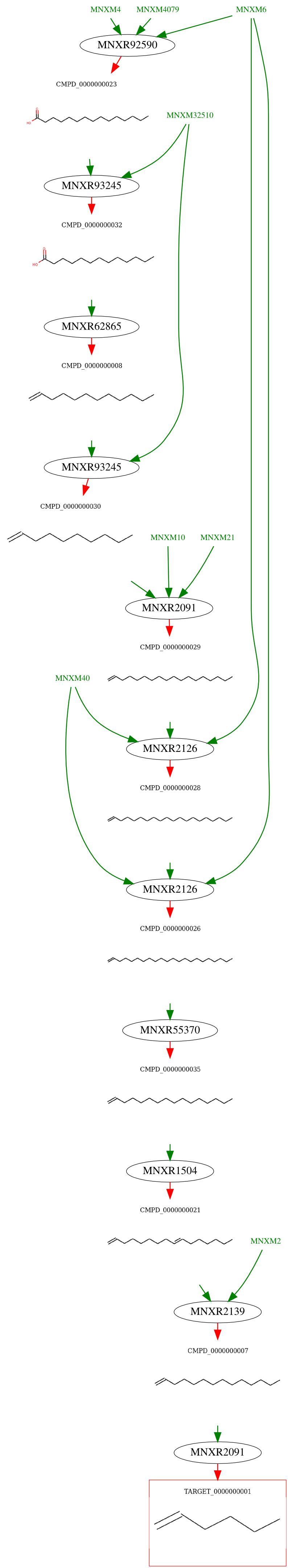

Supplement: Supplementary file 1 — Additional file 1. Monomers maps obtained running Retropath2.0 in section “Virtual screening in the chemical space”. The 17 compounds of the 158 available monomers that can be naturally synthesized and the corresponding synthesis pathways. [file 13321_2017_252_MOESM1_ESM.zip › maps-monomers/butylethylene/path114.png]

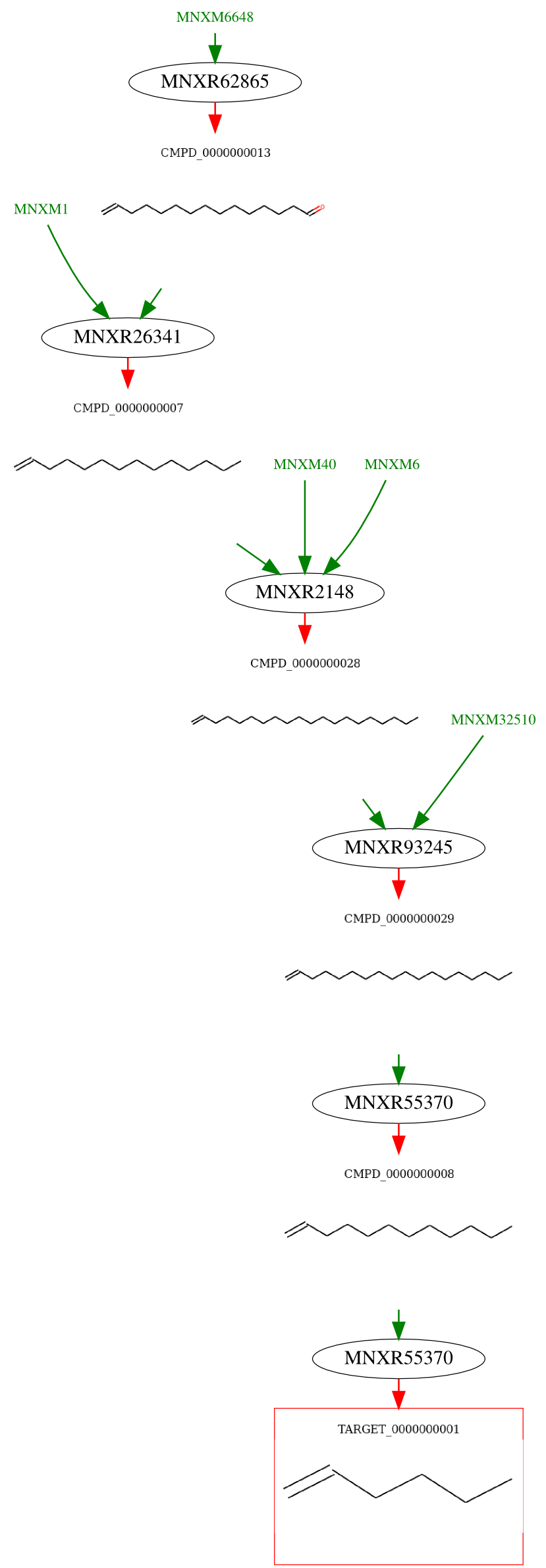

Supplement: Supplementary file 1 — Additional file 1. Monomers maps obtained running Retropath2.0 in section “Virtual screening in the chemical space”. The 17 compounds of the 158 available monomers that can be naturally synthesized and the corresponding synthesis pathways. [file 13321_2017_252_MOESM1_ESM.zip › maps-monomers/butylethylene/path12.png]

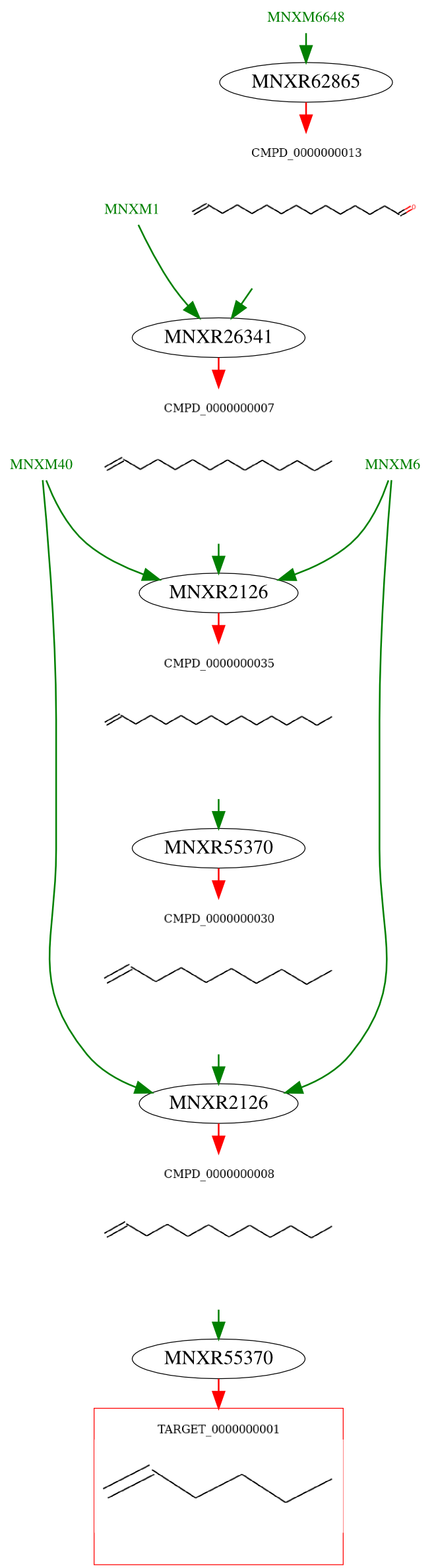

Supplement: Supplementary file 1 — Additional file 1. Monomers maps obtained running Retropath2.0 in section “Virtual screening in the chemical space”. The 17 compounds of the 158 available monomers that can be naturally synthesized and the corresponding synthesis pathways. [file 13321_2017_252_MOESM1_ESM.zip › maps-monomers/butylethylene/path13.png]

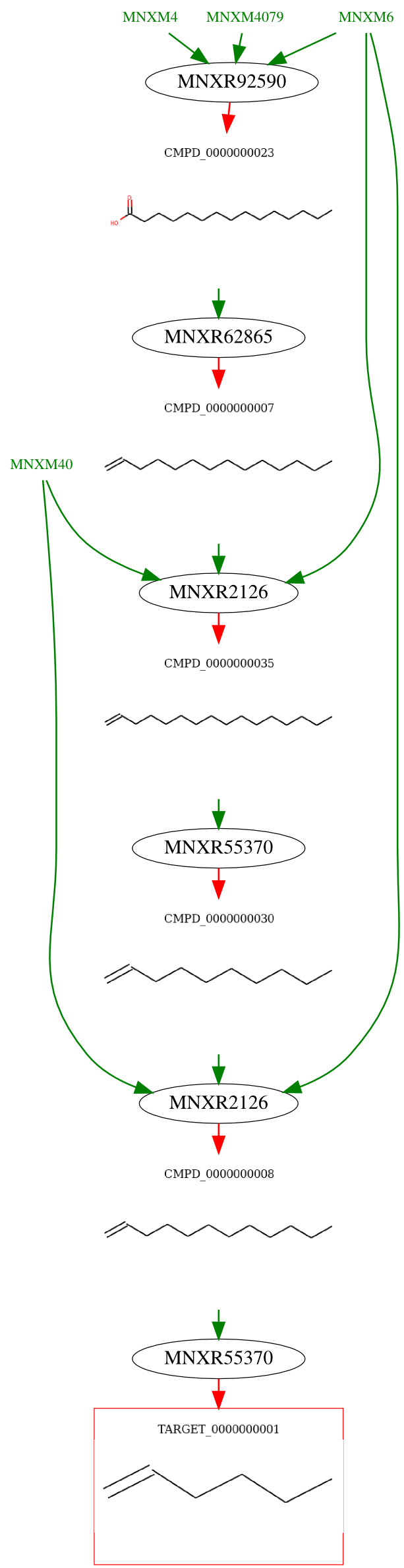

Supplement: Supplementary file 1 — Additional file 1. Monomers maps obtained running Retropath2.0 in section “Virtual screening in the chemical space”. The 17 compounds of the 158 available monomers that can be naturally synthesized and the corresponding synthesis pathways. [file 13321_2017_252_MOESM1_ESM.zip › maps-monomers/butylethylene/path14.png]

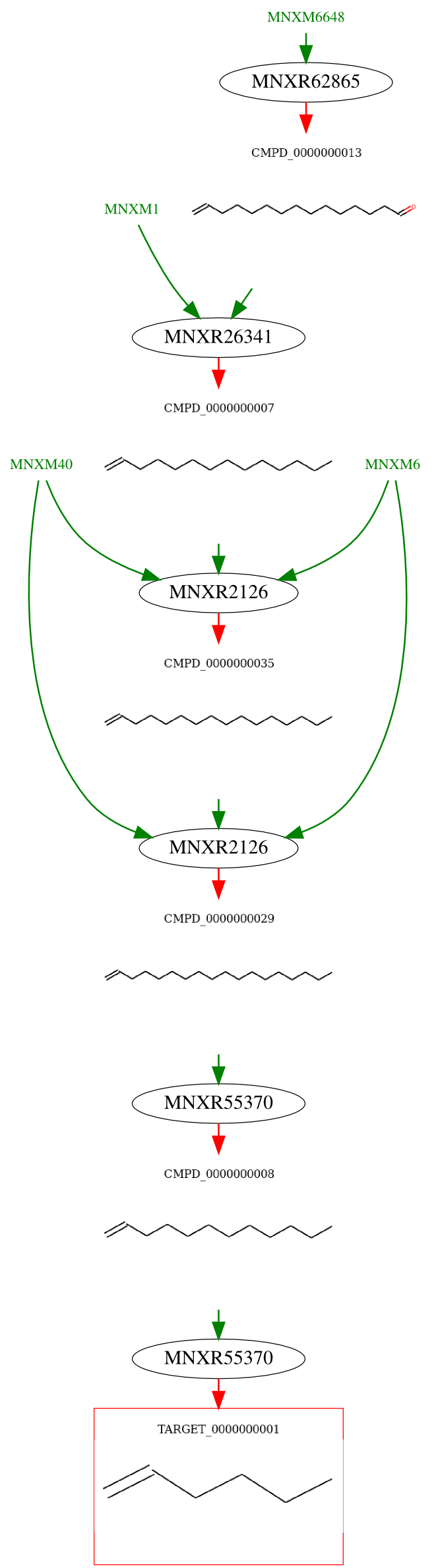

Supplement: Supplementary file 1 — Additional file 1. Monomers maps obtained running Retropath2.0 in section “Virtual screening in the chemical space”. The 17 compounds of the 158 available monomers that can be naturally synthesized and the corresponding synthesis pathways. [file 13321_2017_252_MOESM1_ESM.zip › maps-monomers/butylethylene/path15.png]

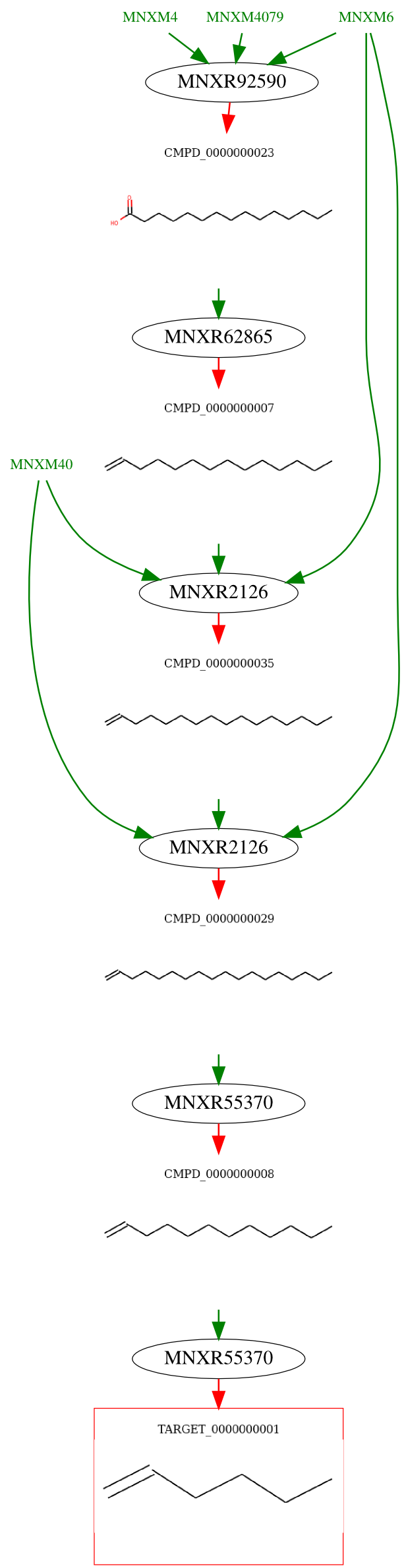

Supplement: Supplementary file 1 — Additional file 1. Monomers maps obtained running Retropath2.0 in section “Virtual screening in the chemical space”. The 17 compounds of the 158 available monomers that can be naturally synthesized and the corresponding synthesis pathways. [file 13321_2017_252_MOESM1_ESM.zip › maps-monomers/butylethylene/path16.png]

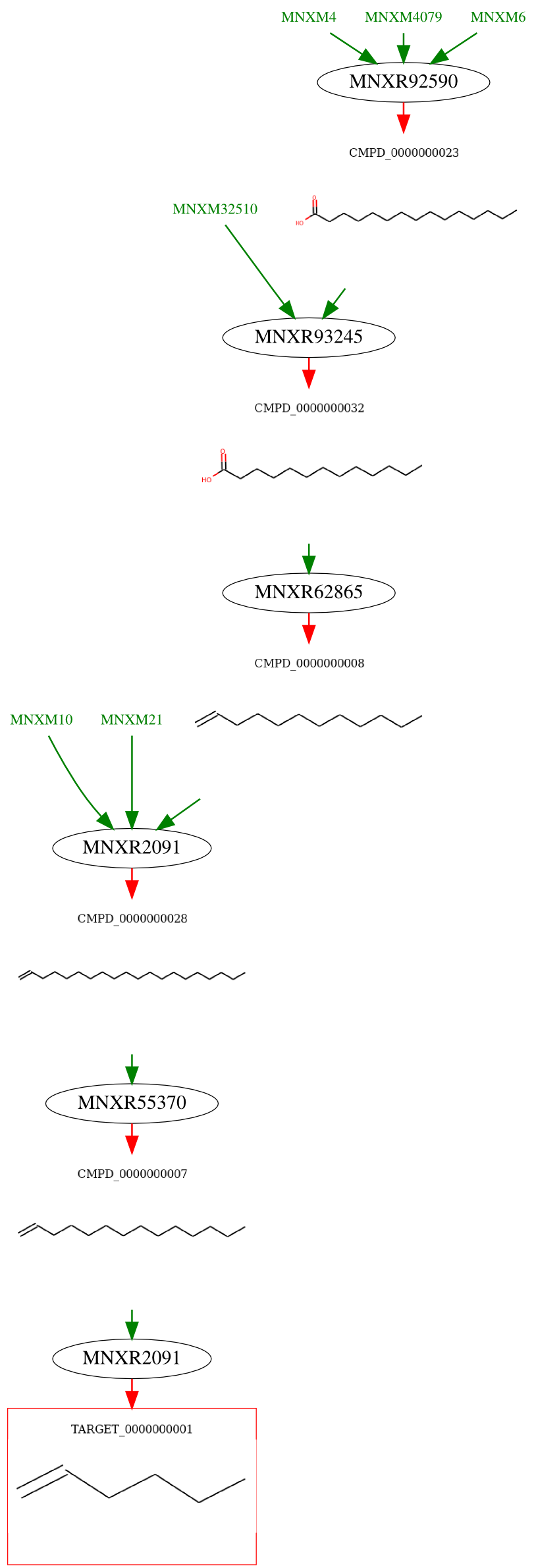

Supplement: Supplementary file 1 — Additional file 1. Monomers maps obtained running Retropath2.0 in section “Virtual screening in the chemical space”. The 17 compounds of the 158 available monomers that can be naturally synthesized and the corresponding synthesis pathways. [file 13321_2017_252_MOESM1_ESM.zip › maps-monomers/butylethylene/path17.png]

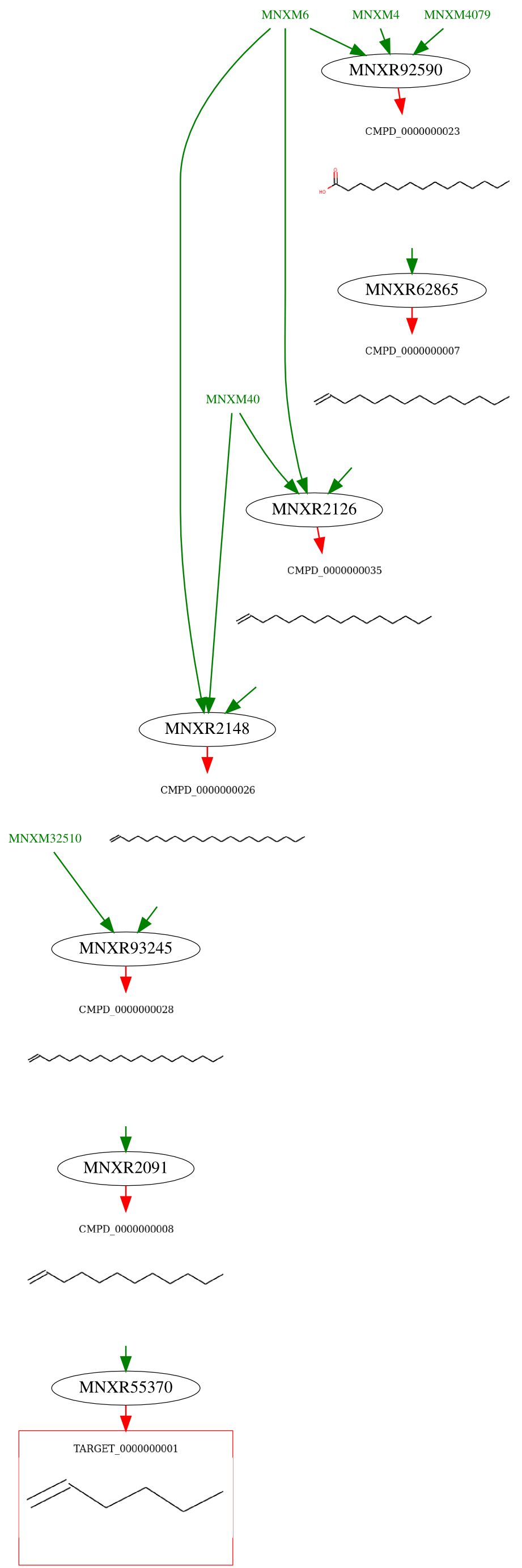

Supplement: Supplementary file 1 — Additional file 1. Monomers maps obtained running Retropath2.0 in section “Virtual screening in the chemical space”. The 17 compounds of the 158 available monomers that can be naturally synthesized and the corresponding synthesis pathways. [file 13321_2017_252_MOESM1_ESM.zip › maps-monomers/butylethylene/path18.png]

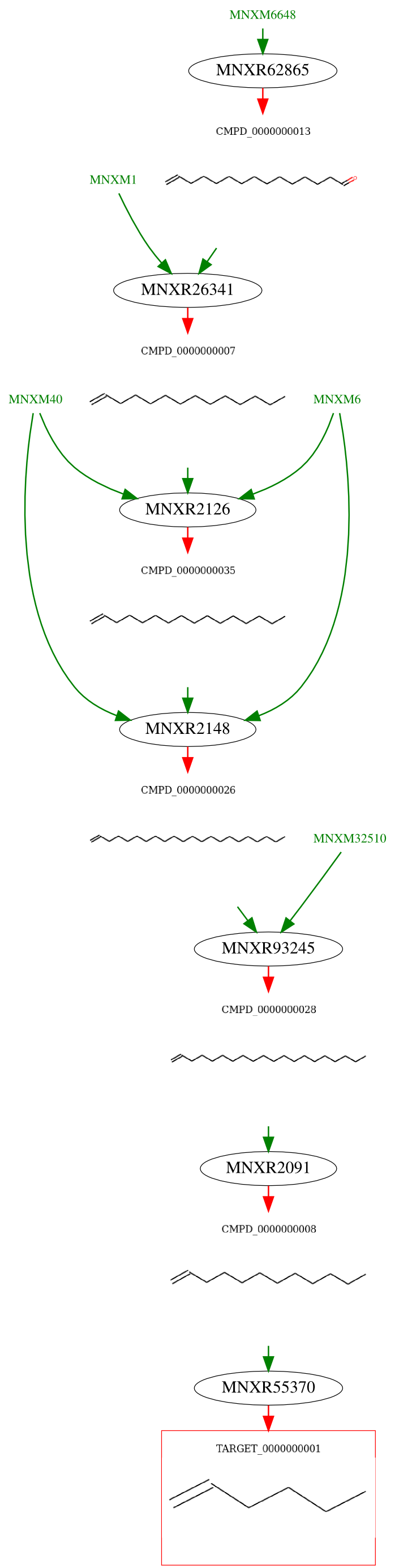

Supplement: Supplementary file 1 — Additional file 1. Monomers maps obtained running Retropath2.0 in section “Virtual screening in the chemical space”. The 17 compounds of the 158 available monomers that can be naturally synthesized and the corresponding synthesis pathways. [file 13321_2017_252_MOESM1_ESM.zip › maps-monomers/butylethylene/path19.png]

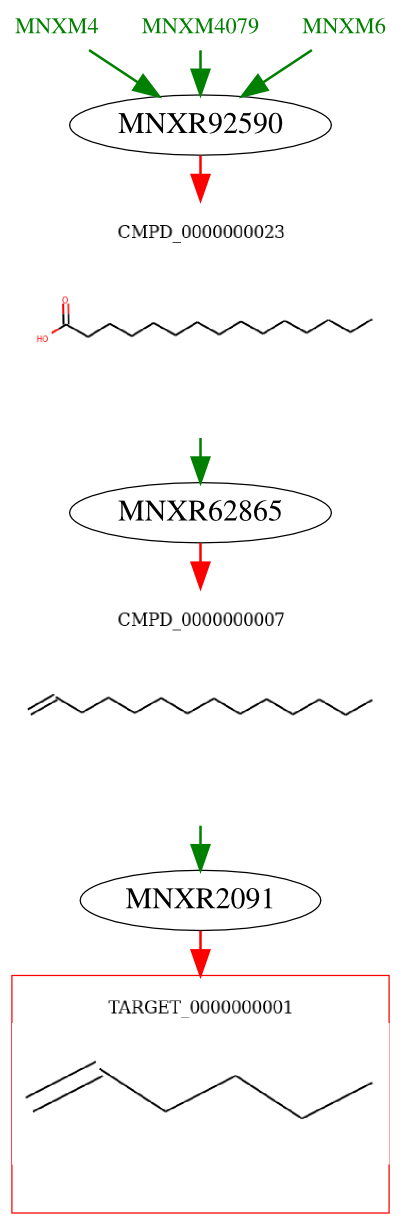

Supplement: Supplementary file 1 — Additional file 1. Monomers maps obtained running Retropath2.0 in section “Virtual screening in the chemical space”. The 17 compounds of the 158 available monomers that can be naturally synthesized and the corresponding synthesis pathways. [file 13321_2017_252_MOESM1_ESM.zip › maps-monomers/butylethylene/path2.png]

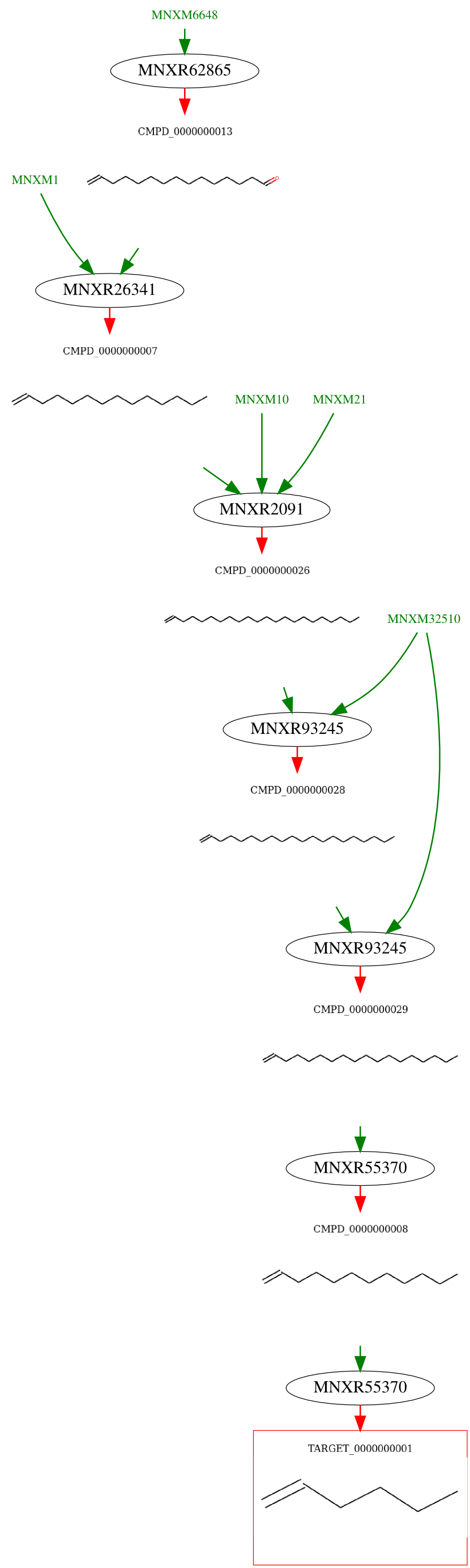

Supplement: Supplementary file 1 — Additional file 1. Monomers maps obtained running Retropath2.0 in section “Virtual screening in the chemical space”. The 17 compounds of the 158 available monomers that can be naturally synthesized and the corresponding synthesis pathways. [file 13321_2017_252_MOESM1_ESM.zip › maps-monomers/butylethylene/path20.png]

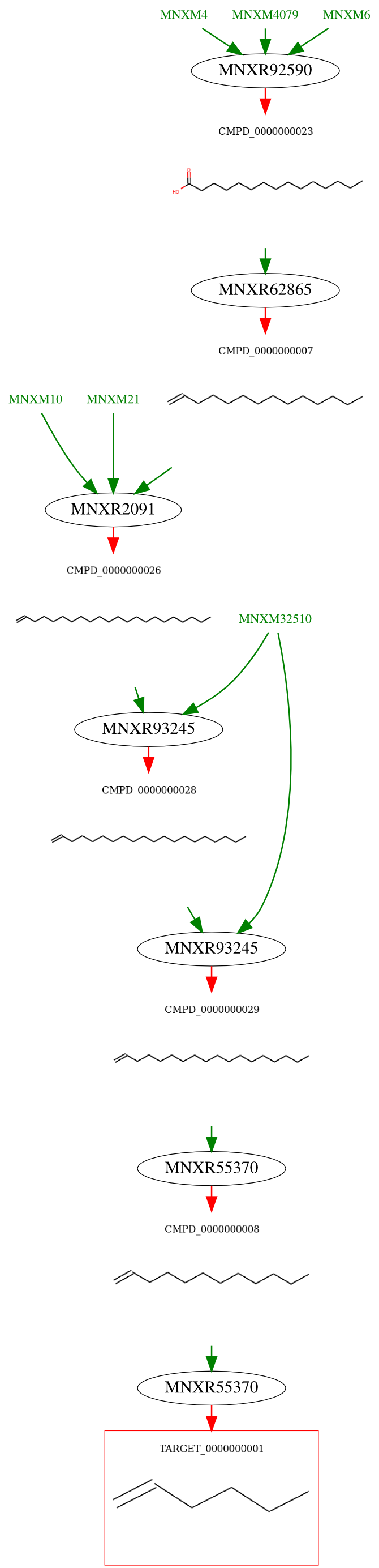

Supplement: Supplementary file 1 — Additional file 1. Monomers maps obtained running Retropath2.0 in section “Virtual screening in the chemical space”. The 17 compounds of the 158 available monomers that can be naturally synthesized and the corresponding synthesis pathways. [file 13321_2017_252_MOESM1_ESM.zip › maps-monomers/butylethylene/path21.png]

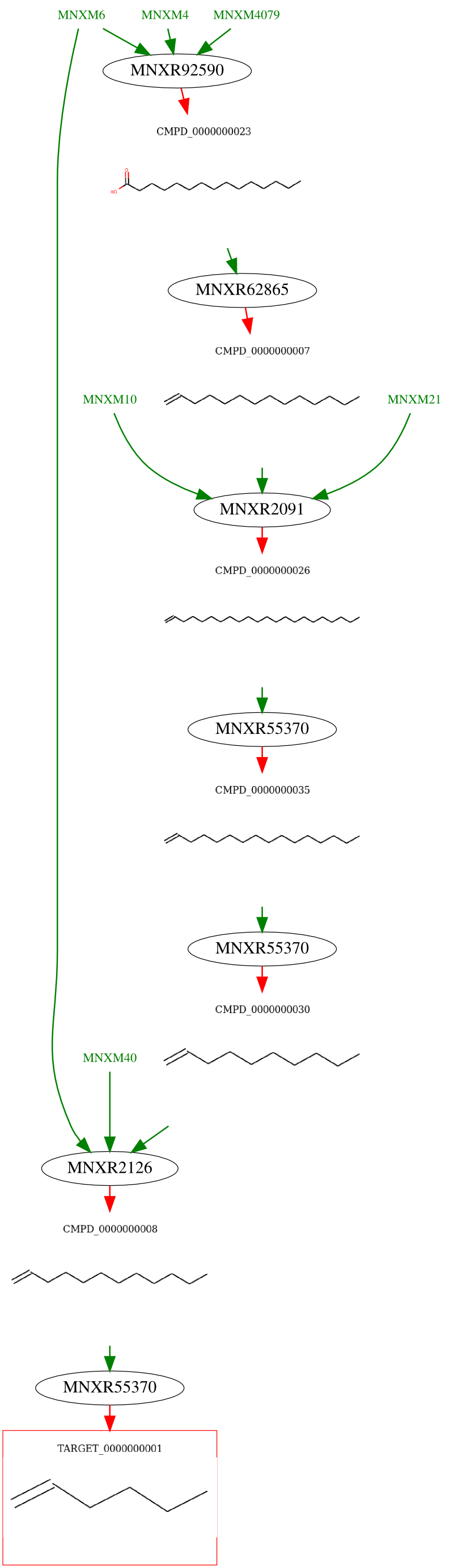

Supplement: Supplementary file 1 — Additional file 1. Monomers maps obtained running Retropath2.0 in section “Virtual screening in the chemical space”. The 17 compounds of the 158 available monomers that can be naturally synthesized and the corresponding synthesis pathways. [file 13321_2017_252_MOESM1_ESM.zip › maps-monomers/butylethylene/path22.png]

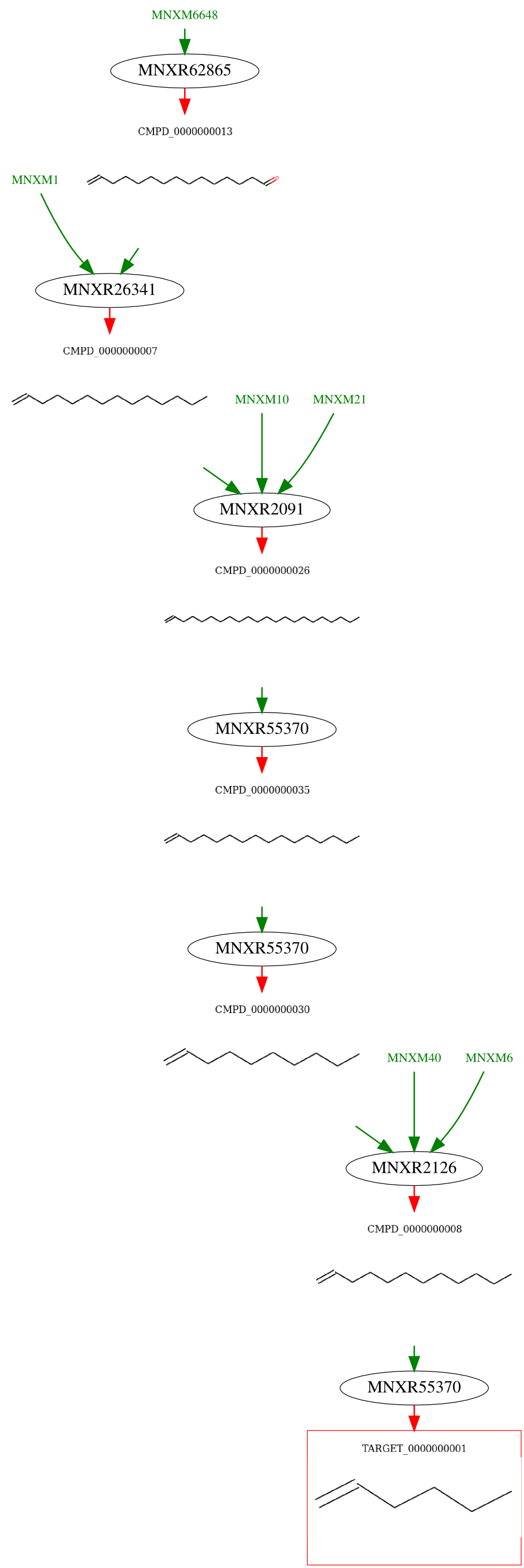

Supplement: Supplementary file 1 — Additional file 1. Monomers maps obtained running Retropath2.0 in section “Virtual screening in the chemical space”. The 17 compounds of the 158 available monomers that can be naturally synthesized and the corresponding synthesis pathways. [file 13321_2017_252_MOESM1_ESM.zip › maps-monomers/butylethylene/path23.png]

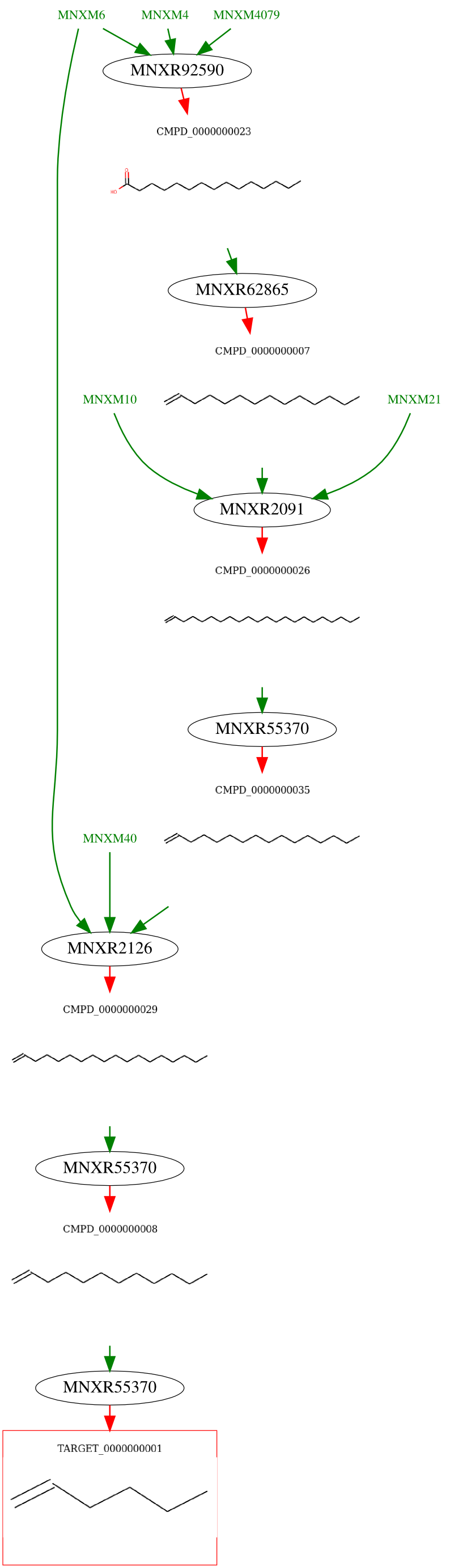

Supplement: Supplementary file 1 — Additional file 1. Monomers maps obtained running Retropath2.0 in section “Virtual screening in the chemical space”. The 17 compounds of the 158 available monomers that can be naturally synthesized and the corresponding synthesis pathways. [file 13321_2017_252_MOESM1_ESM.zip › maps-monomers/butylethylene/path24.png]

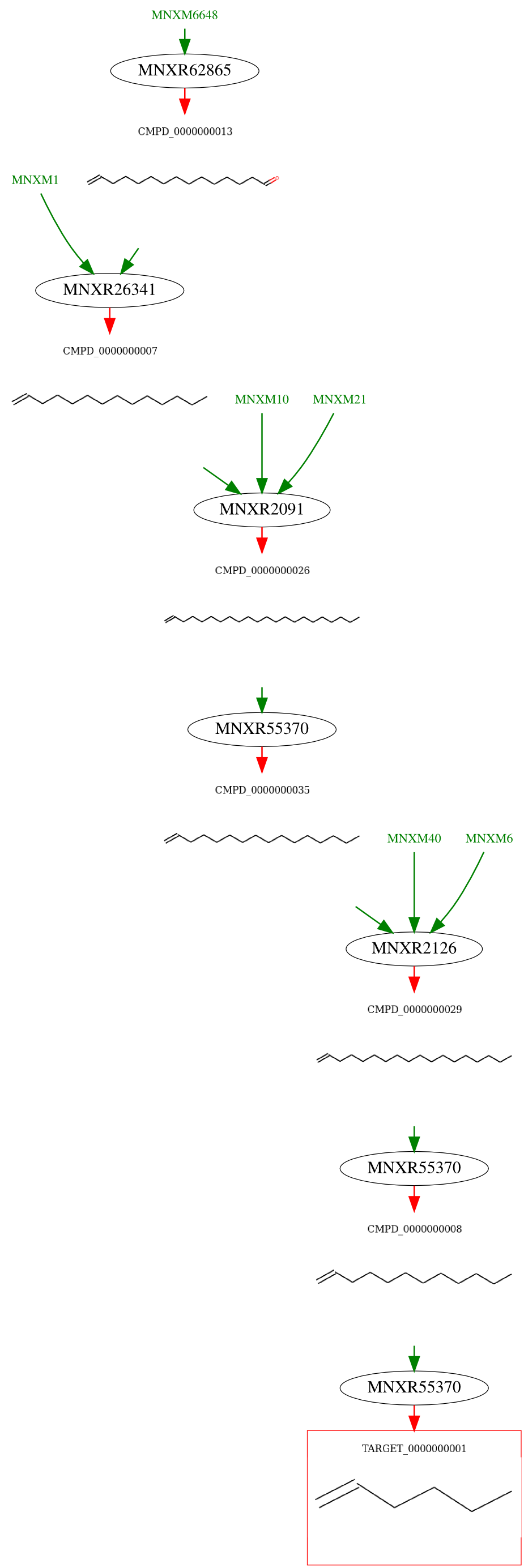

Supplement: Supplementary file 1 — Additional file 1. Monomers maps obtained running Retropath2.0 in section “Virtual screening in the chemical space”. The 17 compounds of the 158 available monomers that can be naturally synthesized and the corresponding synthesis pathways. [file 13321_2017_252_MOESM1_ESM.zip › maps-monomers/butylethylene/path25.png]

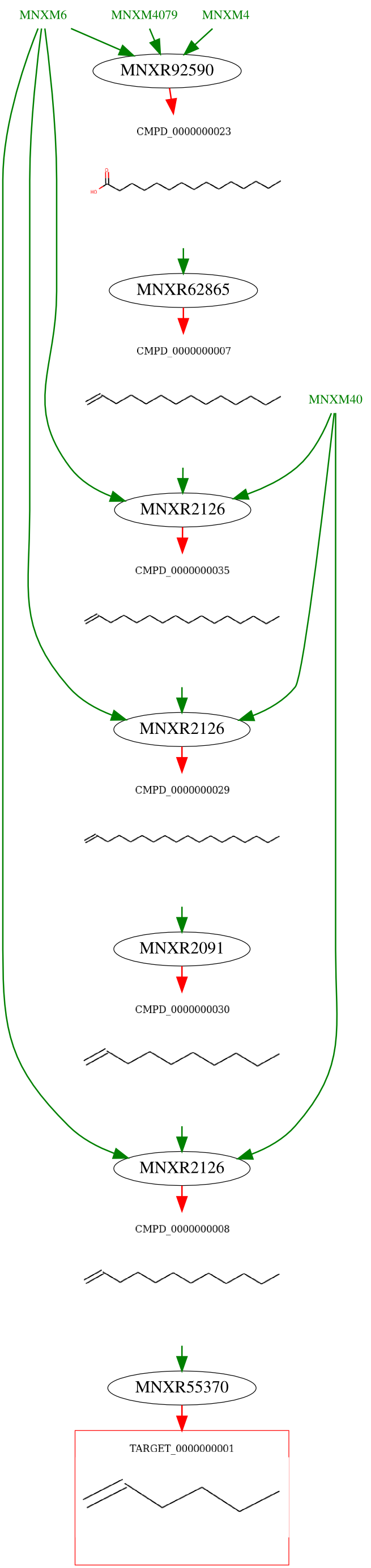

Supplement: Supplementary file 1 — Additional file 1. Monomers maps obtained running Retropath2.0 in section “Virtual screening in the chemical space”. The 17 compounds of the 158 available monomers that can be naturally synthesized and the corresponding synthesis pathways. [file 13321_2017_252_MOESM1_ESM.zip › maps-monomers/butylethylene/path26.png]

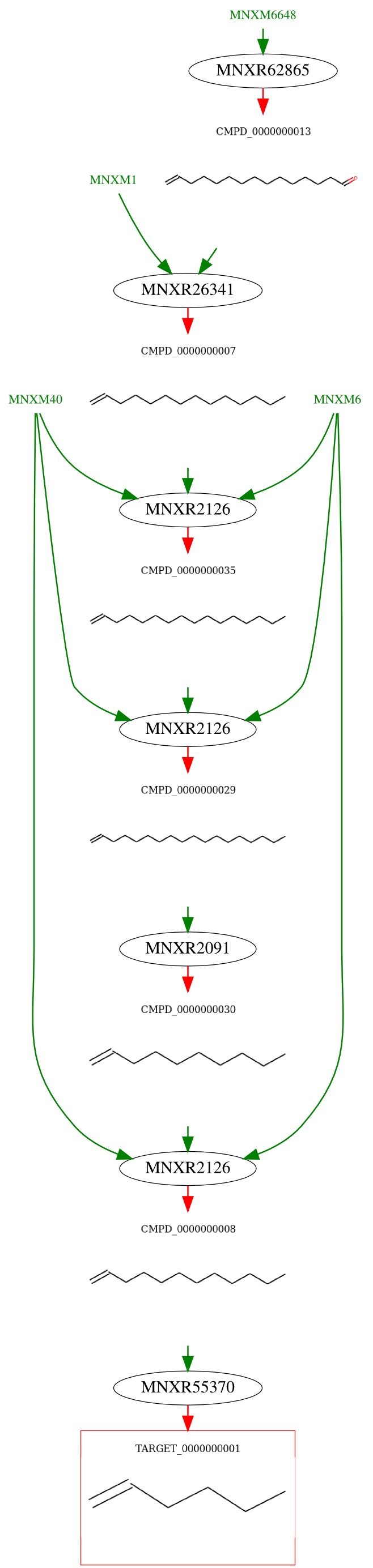

Supplement: Supplementary file 1 — Additional file 1. Monomers maps obtained running Retropath2.0 in section “Virtual screening in the chemical space”. The 17 compounds of the 158 available monomers that can be naturally synthesized and the corresponding synthesis pathways. [file 13321_2017_252_MOESM1_ESM.zip › maps-monomers/butylethylene/path27.png]

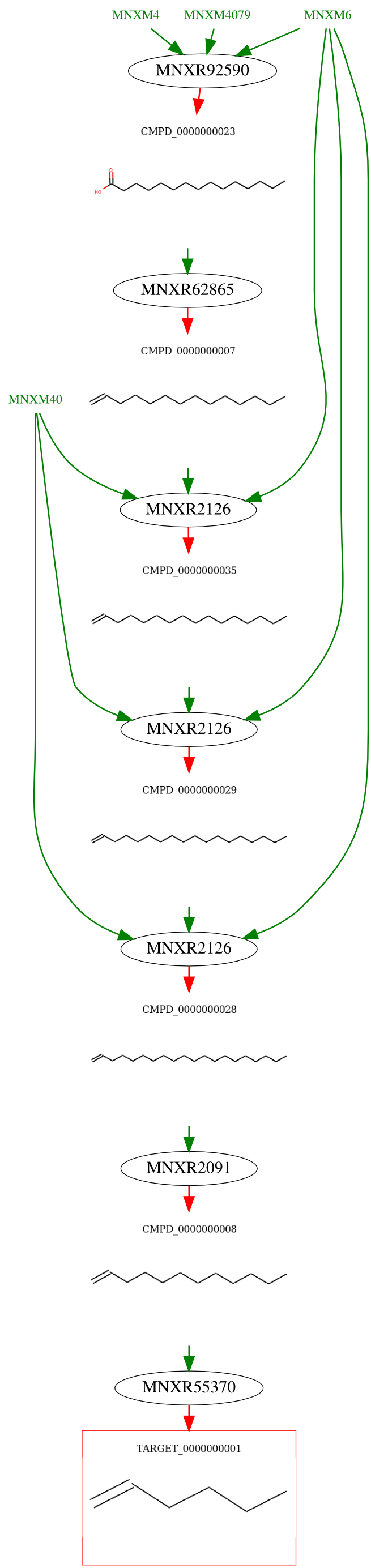

Supplement: Supplementary file 1 — Additional file 1. Monomers maps obtained running Retropath2.0 in section “Virtual screening in the chemical space”. The 17 compounds of the 158 available monomers that can be naturally synthesized and the corresponding synthesis pathways. [file 13321_2017_252_MOESM1_ESM.zip › maps-monomers/butylethylene/path28.png]

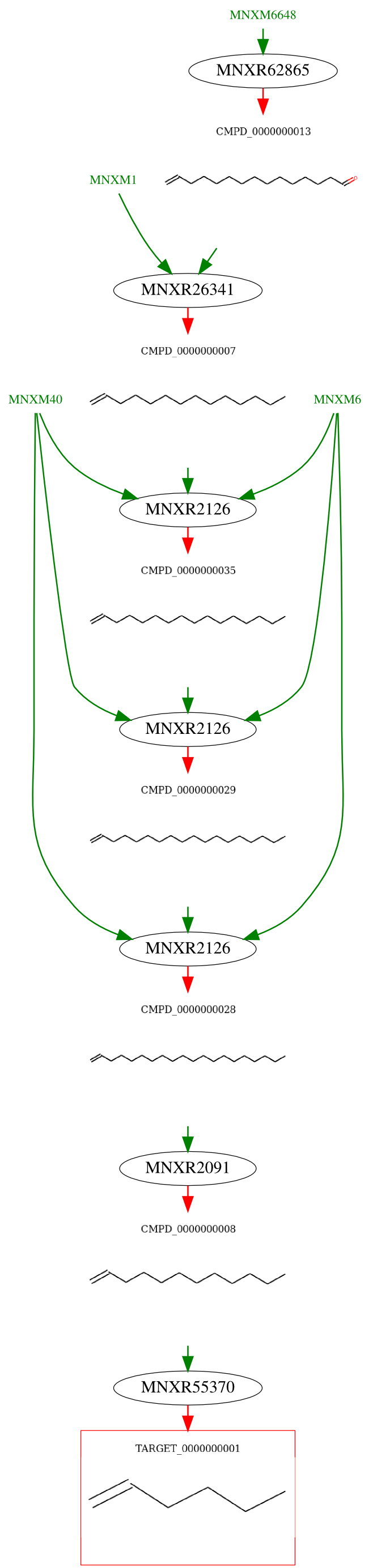

Supplement: Supplementary file 1 — Additional file 1. Monomers maps obtained running Retropath2.0 in section “Virtual screening in the chemical space”. The 17 compounds of the 158 available monomers that can be naturally synthesized and the corresponding synthesis pathways. [file 13321_2017_252_MOESM1_ESM.zip › maps-monomers/butylethylene/path29.png]

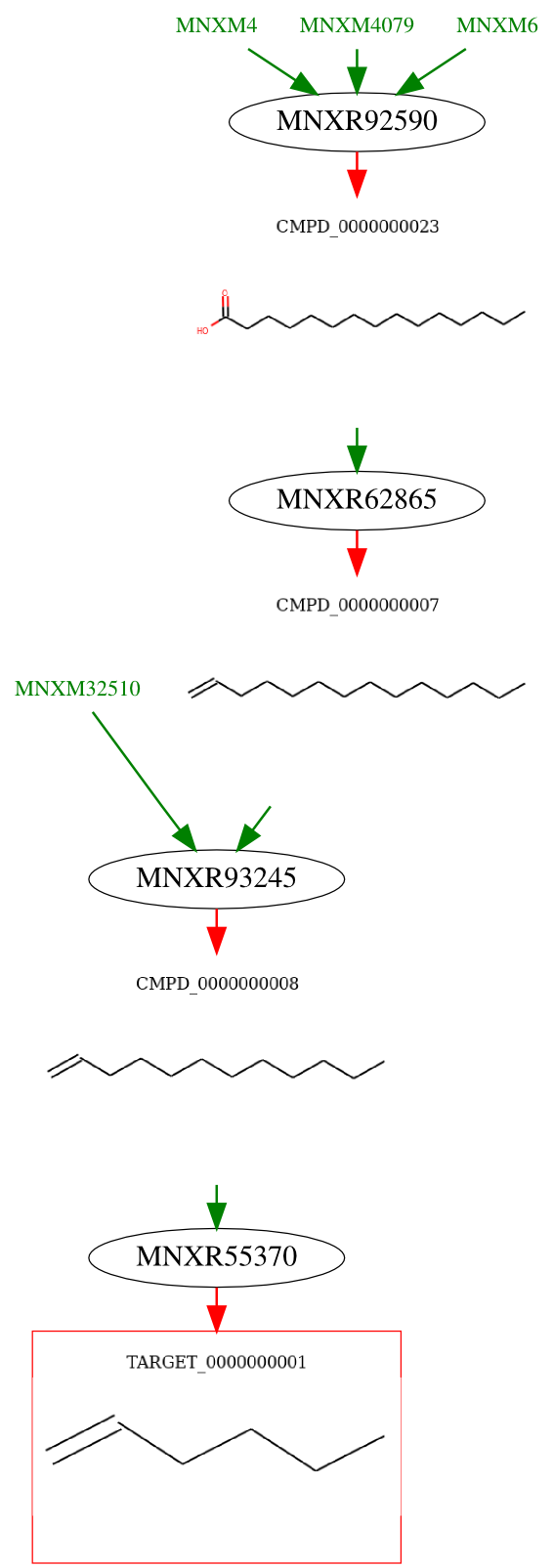

Supplement: Supplementary file 1 — Additional file 1. Monomers maps obtained running Retropath2.0 in section “Virtual screening in the chemical space”. The 17 compounds of the 158 available monomers that can be naturally synthesized and the corresponding synthesis pathways. [file 13321_2017_252_MOESM1_ESM.zip › maps-monomers/butylethylene/path3.png]

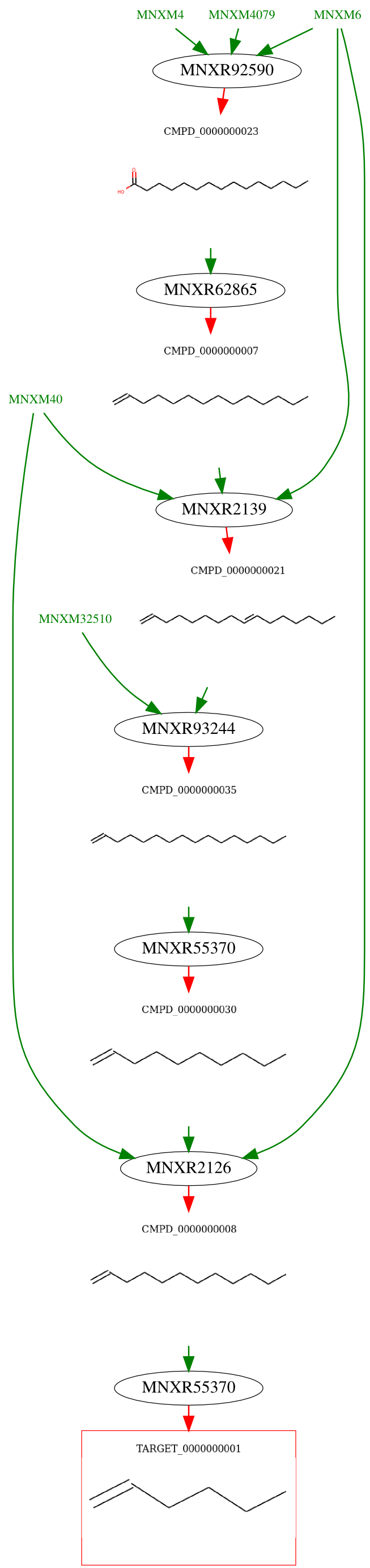

Supplement: Supplementary file 1 — Additional file 1. Monomers maps obtained running Retropath2.0 in section “Virtual screening in the chemical space”. The 17 compounds of the 158 available monomers that can be naturally synthesized and the corresponding synthesis pathways. [file 13321_2017_252_MOESM1_ESM.zip › maps-monomers/butylethylene/path30.png]

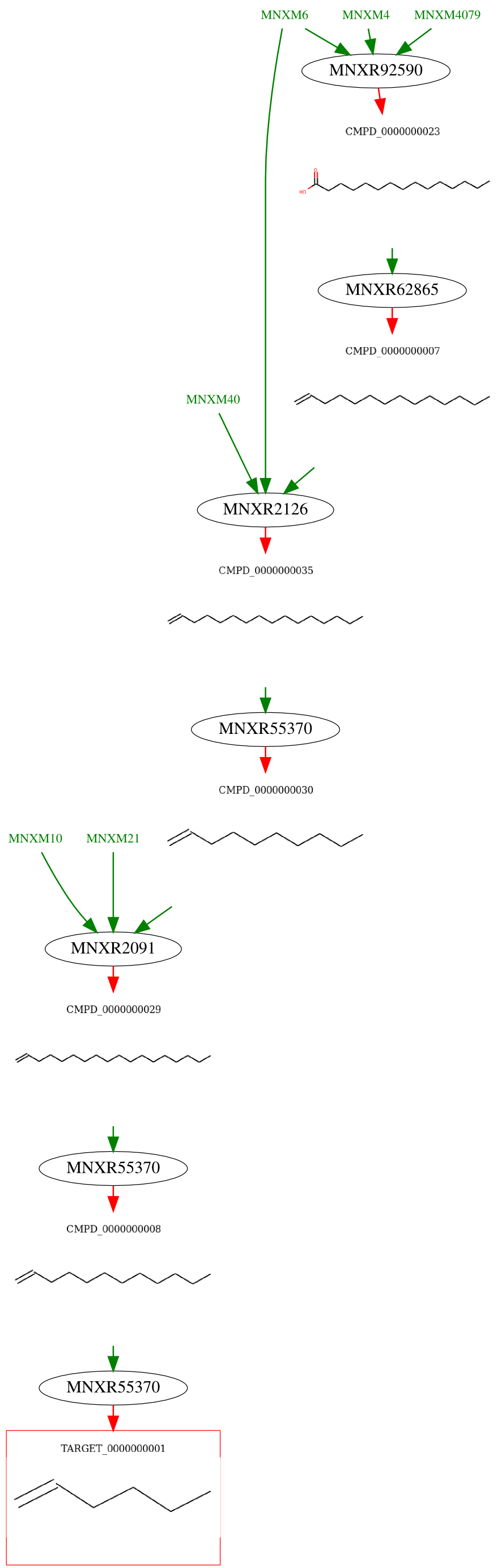

Supplement: Supplementary file 1 — Additional file 1. Monomers maps obtained running Retropath2.0 in section “Virtual screening in the chemical space”. The 17 compounds of the 158 available monomers that can be naturally synthesized and the corresponding synthesis pathways. [file 13321_2017_252_MOESM1_ESM.zip › maps-monomers/butylethylene/path31.png]

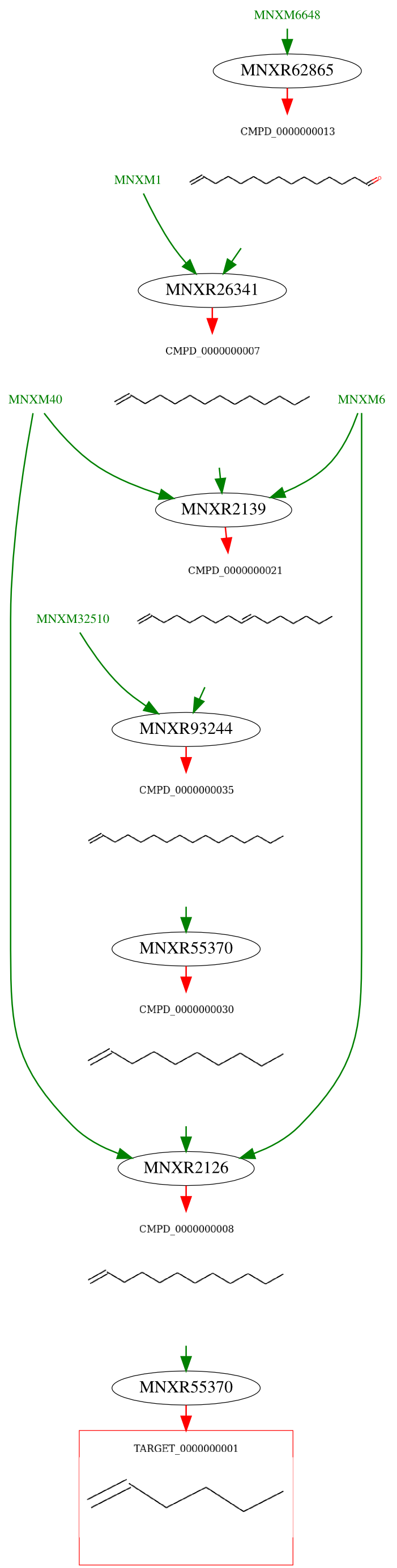

Supplement: Supplementary file 1 — Additional file 1. Monomers maps obtained running Retropath2.0 in section “Virtual screening in the chemical space”. The 17 compounds of the 158 available monomers that can be naturally synthesized and the corresponding synthesis pathways. [file 13321_2017_252_MOESM1_ESM.zip › maps-monomers/butylethylene/path32.png]

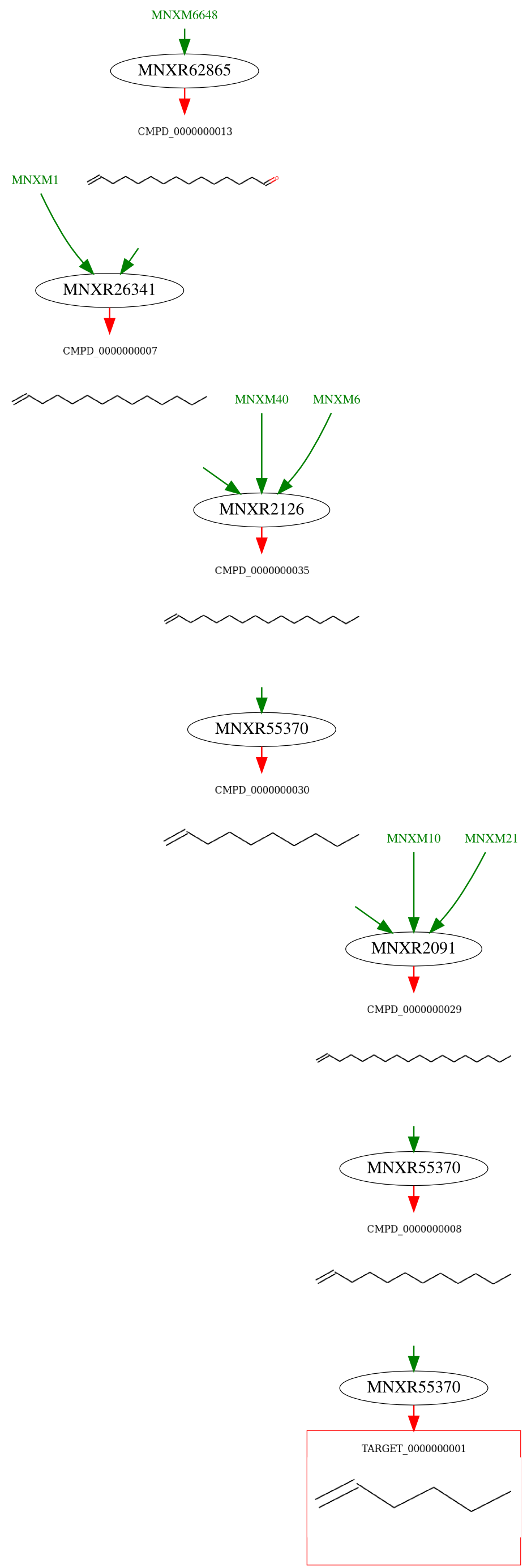

Supplement: Supplementary file 1 — Additional file 1. Monomers maps obtained running Retropath2.0 in section “Virtual screening in the chemical space”. The 17 compounds of the 158 available monomers that can be naturally synthesized and the corresponding synthesis pathways. [file 13321_2017_252_MOESM1_ESM.zip › maps-monomers/butylethylene/path33.png]

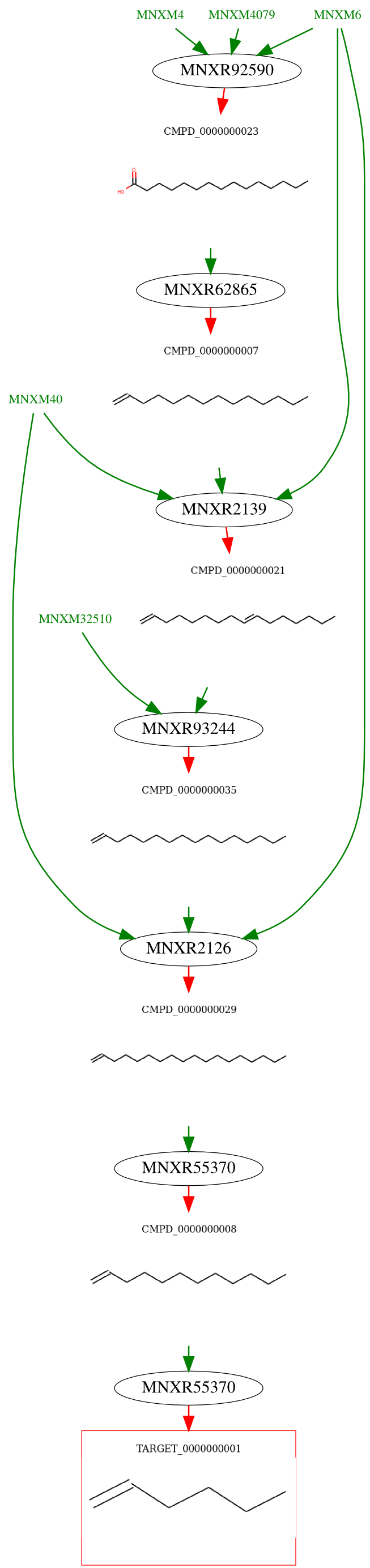

Supplement: Supplementary file 1 — Additional file 1. Monomers maps obtained running Retropath2.0 in section “Virtual screening in the chemical space”. The 17 compounds of the 158 available monomers that can be naturally synthesized and the corresponding synthesis pathways. [file 13321_2017_252_MOESM1_ESM.zip › maps-monomers/butylethylene/path34.png]

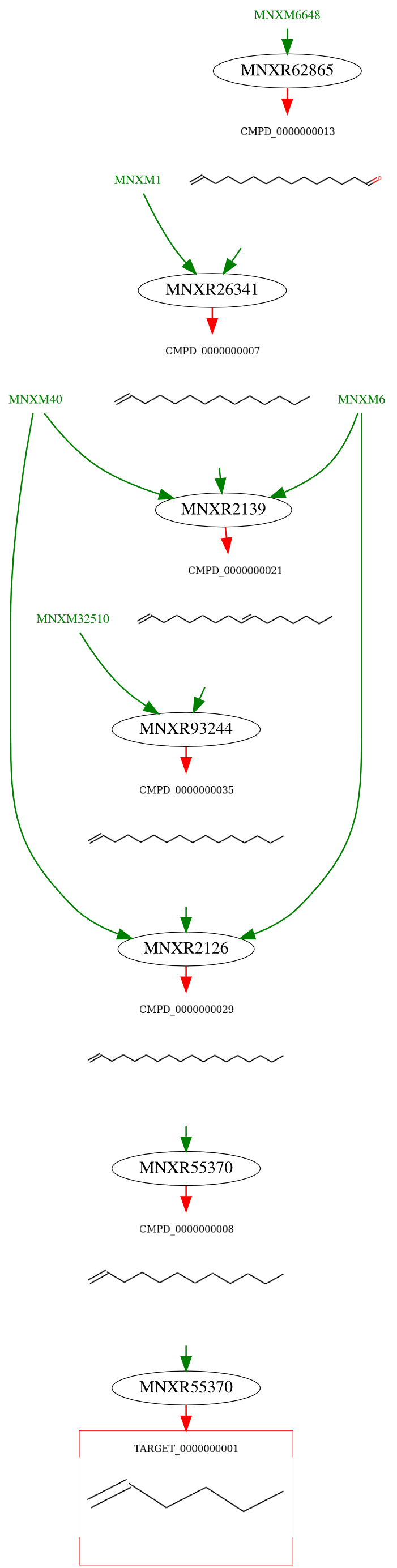

Supplement: Supplementary file 1 — Additional file 1. Monomers maps obtained running Retropath2.0 in section “Virtual screening in the chemical space”. The 17 compounds of the 158 available monomers that can be naturally synthesized and the corresponding synthesis pathways. [file 13321_2017_252_MOESM1_ESM.zip › maps-monomers/butylethylene/path35.png]

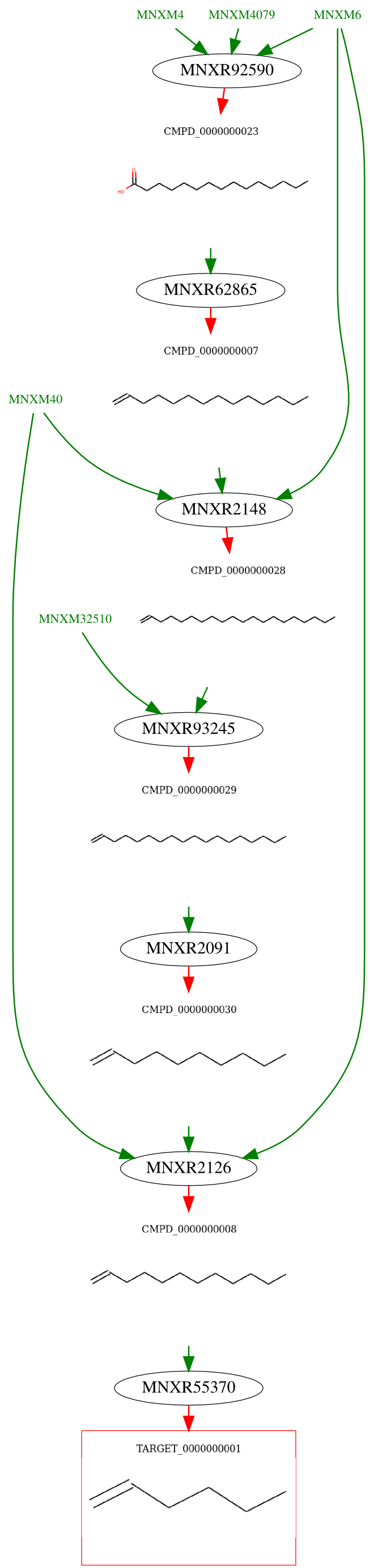

Supplement: Supplementary file 1 — Additional file 1. Monomers maps obtained running Retropath2.0 in section “Virtual screening in the chemical space”. The 17 compounds of the 158 available monomers that can be naturally synthesized and the corresponding synthesis pathways. [file 13321_2017_252_MOESM1_ESM.zip › maps-monomers/butylethylene/path36.png]

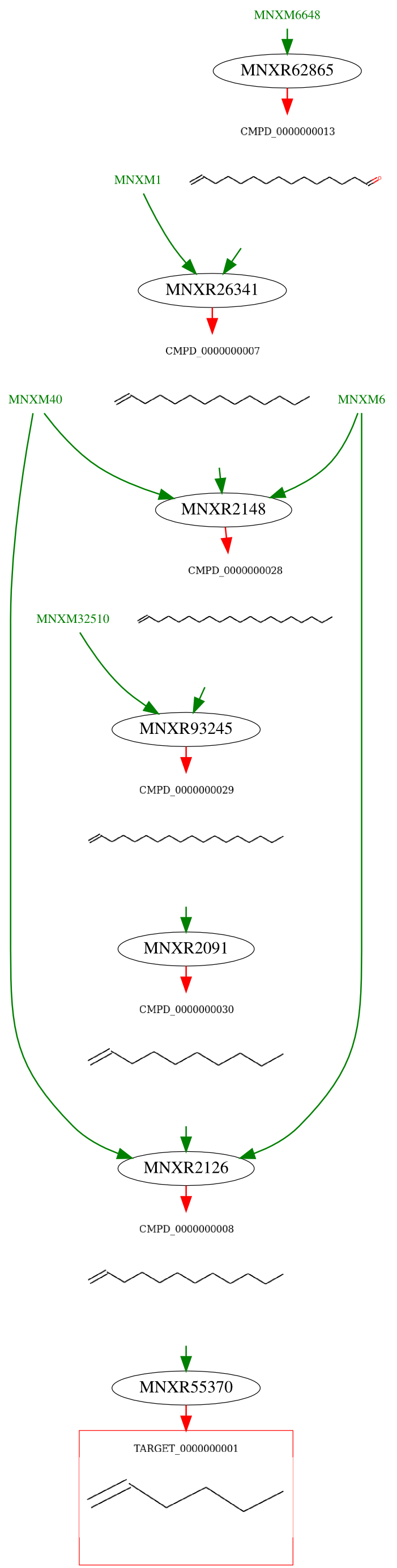

Supplement: Supplementary file 1 — Additional file 1. Monomers maps obtained running Retropath2.0 in section “Virtual screening in the chemical space”. The 17 compounds of the 158 available monomers that can be naturally synthesized and the corresponding synthesis pathways. [file 13321_2017_252_MOESM1_ESM.zip › maps-monomers/butylethylene/path37.png]

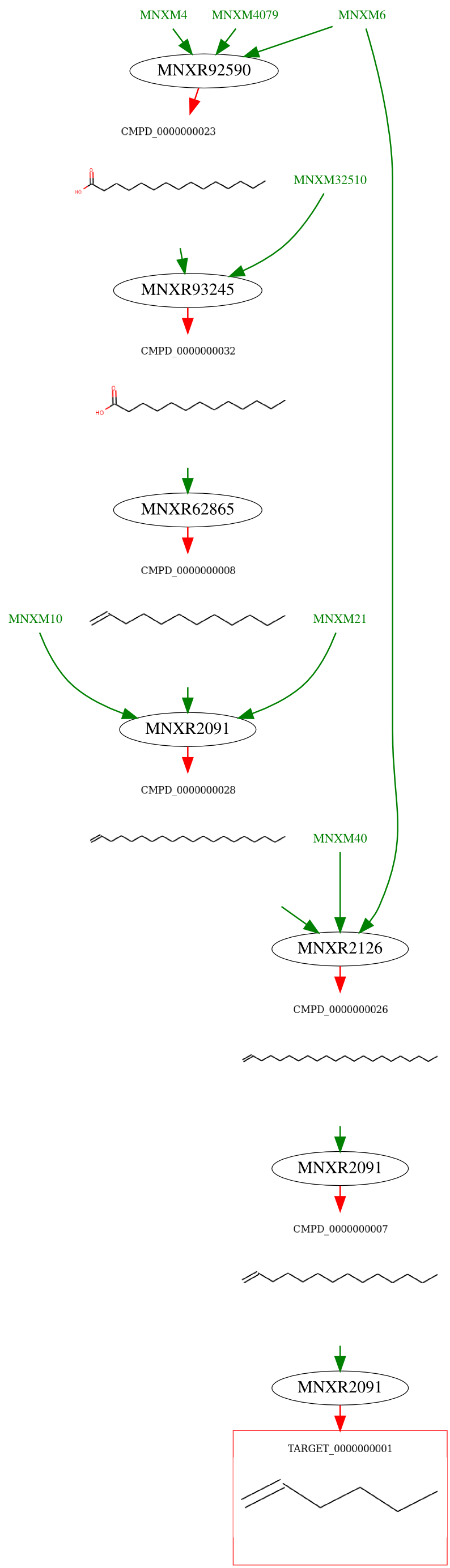

Supplement: Supplementary file 1 — Additional file 1. Monomers maps obtained running Retropath2.0 in section “Virtual screening in the chemical space”. The 17 compounds of the 158 available monomers that can be naturally synthesized and the corresponding synthesis pathways. [file 13321_2017_252_MOESM1_ESM.zip › maps-monomers/butylethylene/path38.png]

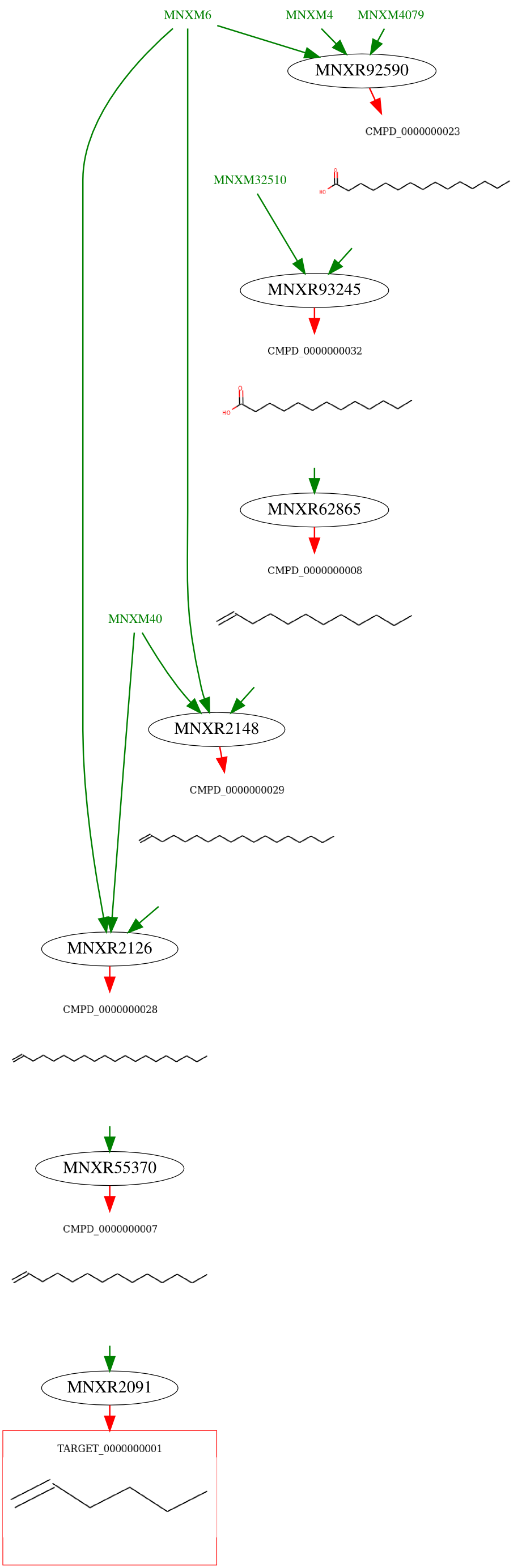

Supplement: Supplementary file 1 — Additional file 1. Monomers maps obtained running Retropath2.0 in section “Virtual screening in the chemical space”. The 17 compounds of the 158 available monomers that can be naturally synthesized and the corresponding synthesis pathways. [file 13321_2017_252_MOESM1_ESM.zip › maps-monomers/butylethylene/path39.png]

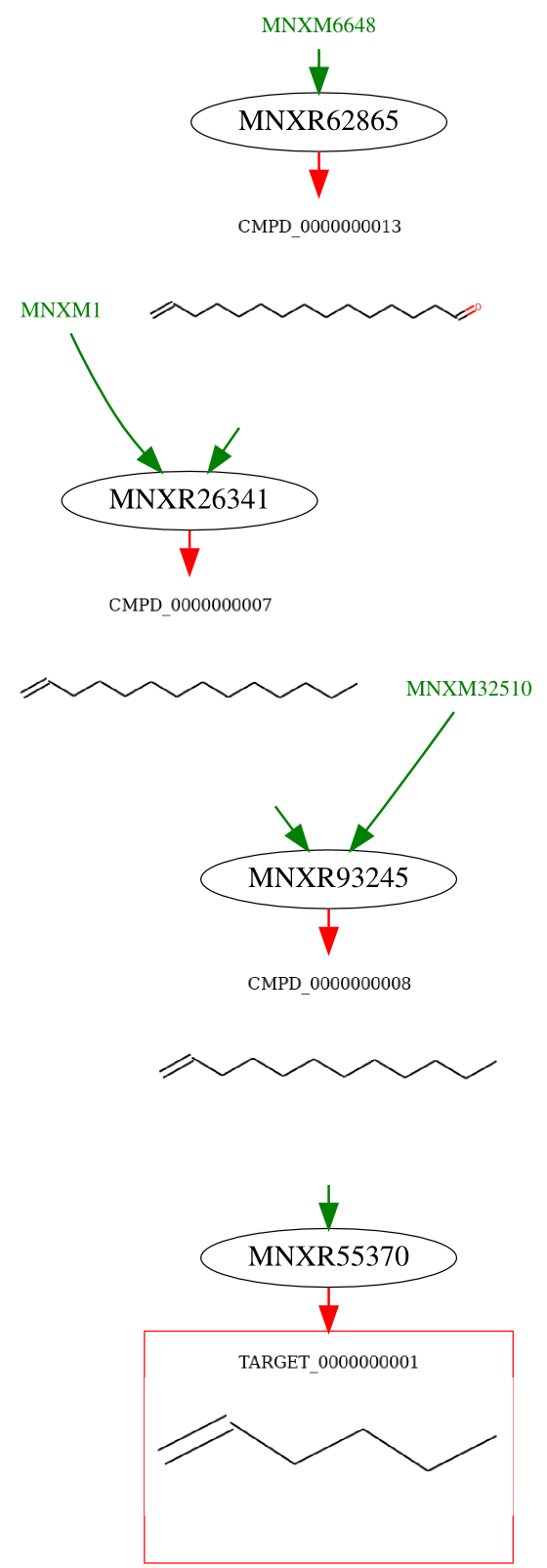

Supplement: Supplementary file 1 — Additional file 1. Monomers maps obtained running Retropath2.0 in section “Virtual screening in the chemical space”. The 17 compounds of the 158 available monomers that can be naturally synthesized and the corresponding synthesis pathways. [file 13321_2017_252_MOESM1_ESM.zip › maps-monomers/butylethylene/path4.png]

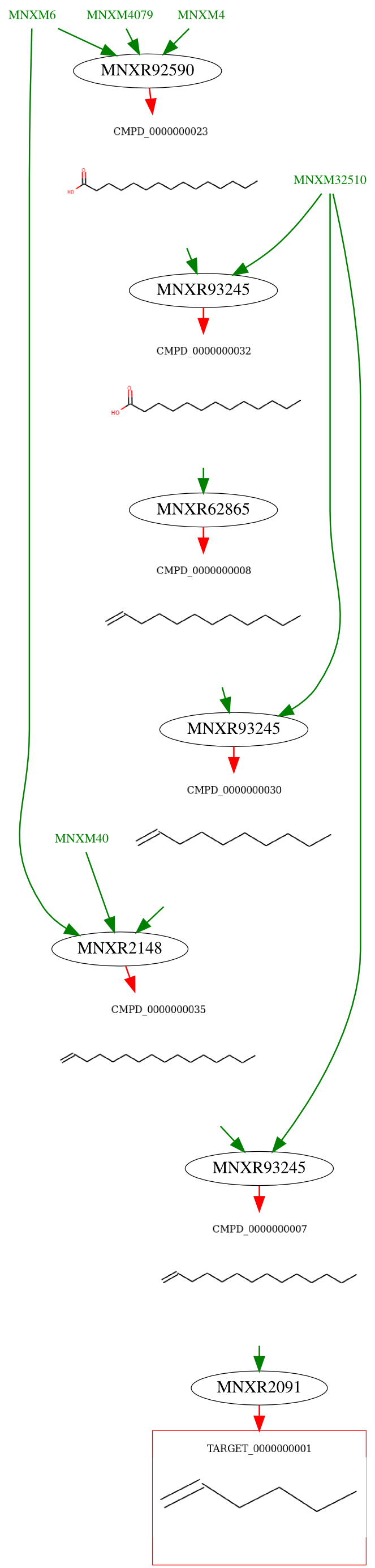

Supplement: Supplementary file 1 — Additional file 1. Monomers maps obtained running Retropath2.0 in section “Virtual screening in the chemical space”. The 17 compounds of the 158 available monomers that can be naturally synthesized and the corresponding synthesis pathways. [file 13321_2017_252_MOESM1_ESM.zip › maps-monomers/butylethylene/path40.png]

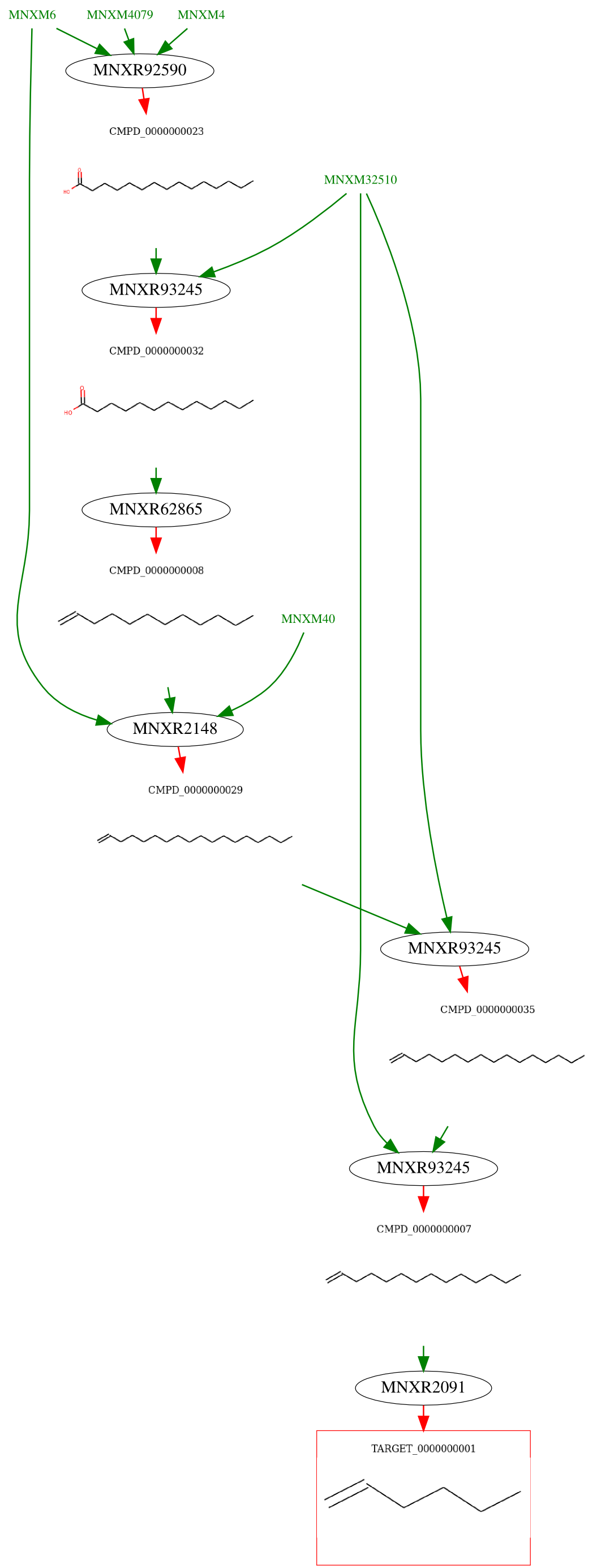

Supplement: Supplementary file 1 — Additional file 1. Monomers maps obtained running Retropath2.0 in section “Virtual screening in the chemical space”. The 17 compounds of the 158 available monomers that can be naturally synthesized and the corresponding synthesis pathways. [file 13321_2017_252_MOESM1_ESM.zip › maps-monomers/butylethylene/path41.png]

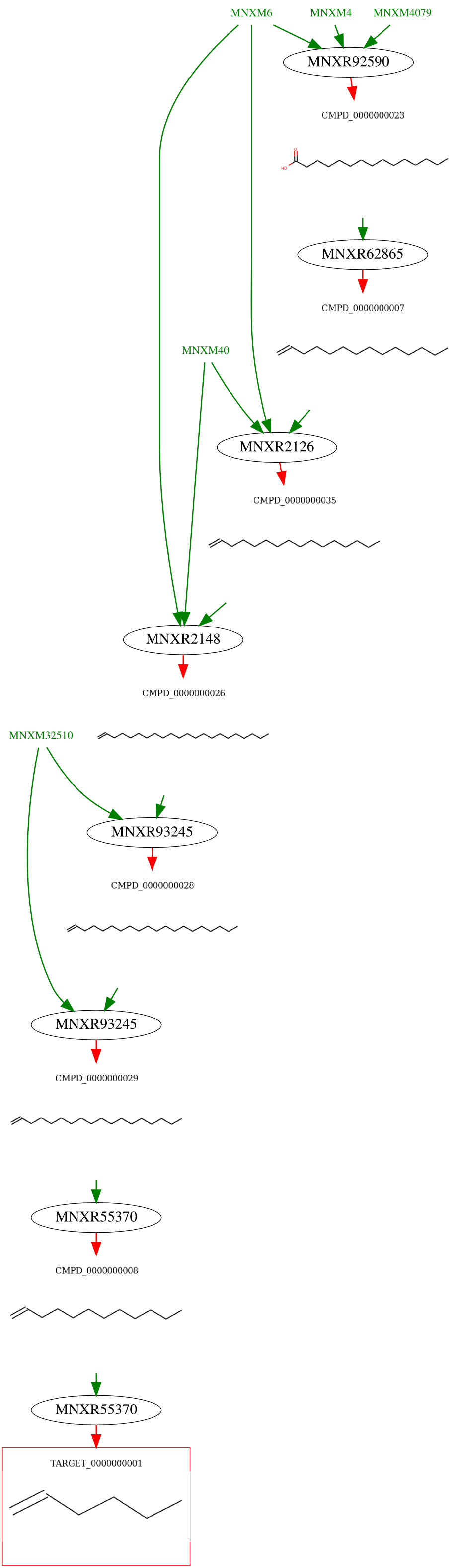

Supplement: Supplementary file 1 — Additional file 1. Monomers maps obtained running Retropath2.0 in section “Virtual screening in the chemical space”. The 17 compounds of the 158 available monomers that can be naturally synthesized and the corresponding synthesis pathways. [file 13321_2017_252_MOESM1_ESM.zip › maps-monomers/butylethylene/path42.png]

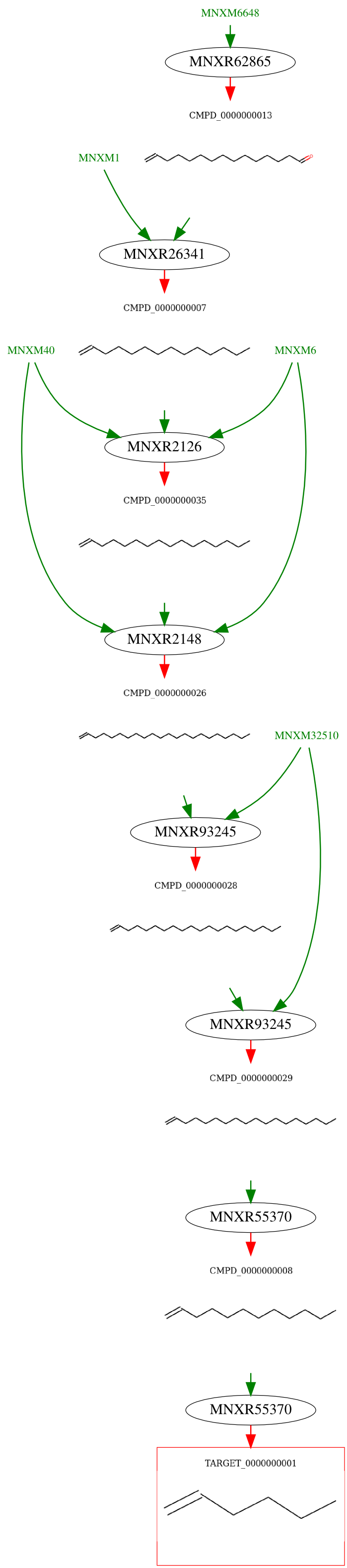

Supplement: Supplementary file 1 — Additional file 1. Monomers maps obtained running Retropath2.0 in section “Virtual screening in the chemical space”. The 17 compounds of the 158 available monomers that can be naturally synthesized and the corresponding synthesis pathways. [file 13321_2017_252_MOESM1_ESM.zip › maps-monomers/butylethylene/path43.png]

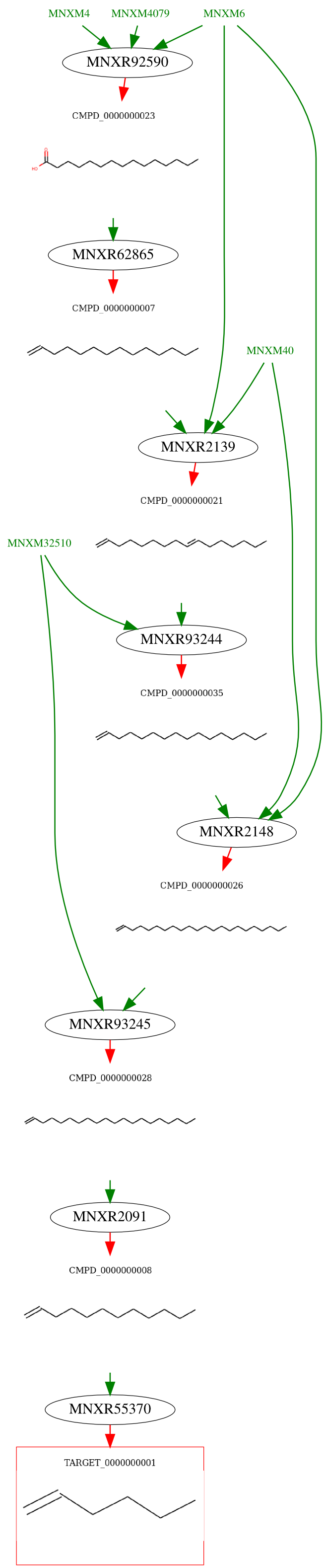

Supplement: Supplementary file 1 — Additional file 1. Monomers maps obtained running Retropath2.0 in section “Virtual screening in the chemical space”. The 17 compounds of the 158 available monomers that can be naturally synthesized and the corresponding synthesis pathways. [file 13321_2017_252_MOESM1_ESM.zip › maps-monomers/butylethylene/path44.png]

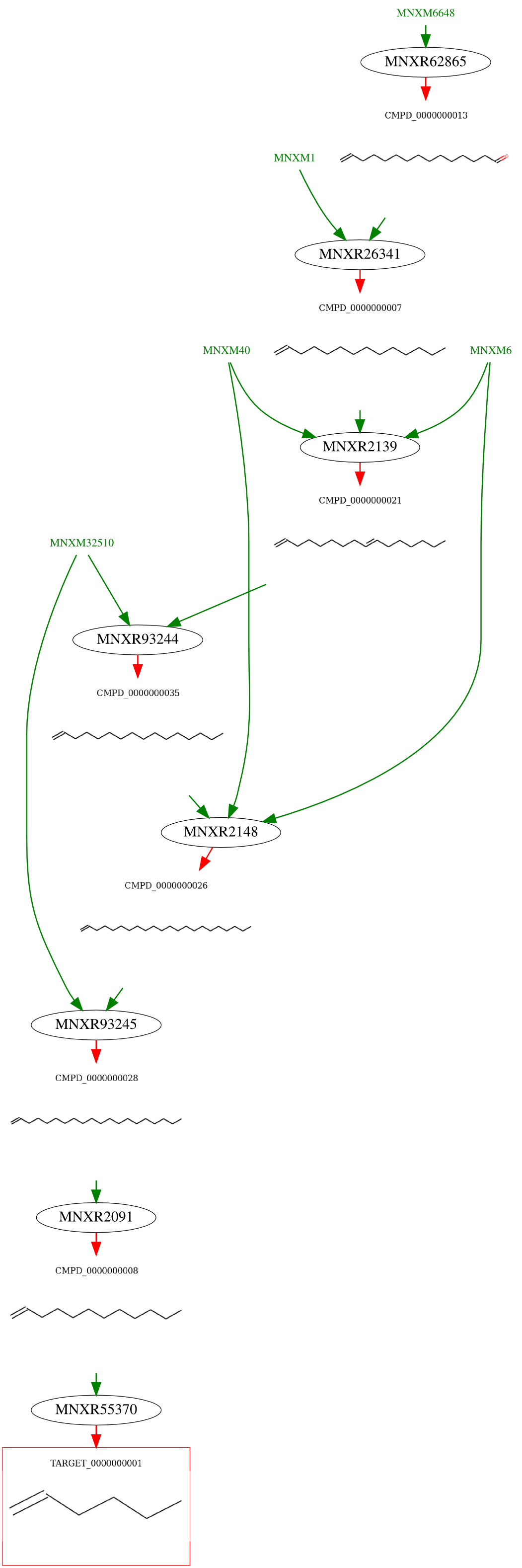

Supplement: Supplementary file 1 — Additional file 1. Monomers maps obtained running Retropath2.0 in section “Virtual screening in the chemical space”. The 17 compounds of the 158 available monomers that can be naturally synthesized and the corresponding synthesis pathways. [file 13321_2017_252_MOESM1_ESM.zip › maps-monomers/butylethylene/path45.png]

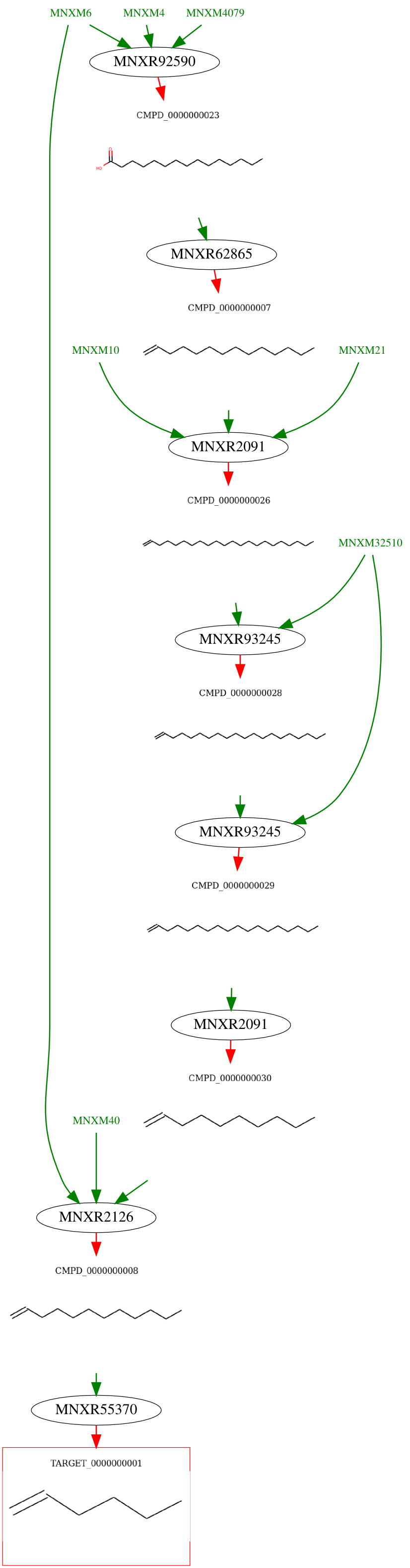

Supplement: Supplementary file 1 — Additional file 1. Monomers maps obtained running Retropath2.0 in section “Virtual screening in the chemical space”. The 17 compounds of the 158 available monomers that can be naturally synthesized and the corresponding synthesis pathways. [file 13321_2017_252_MOESM1_ESM.zip › maps-monomers/butylethylene/path46.png]

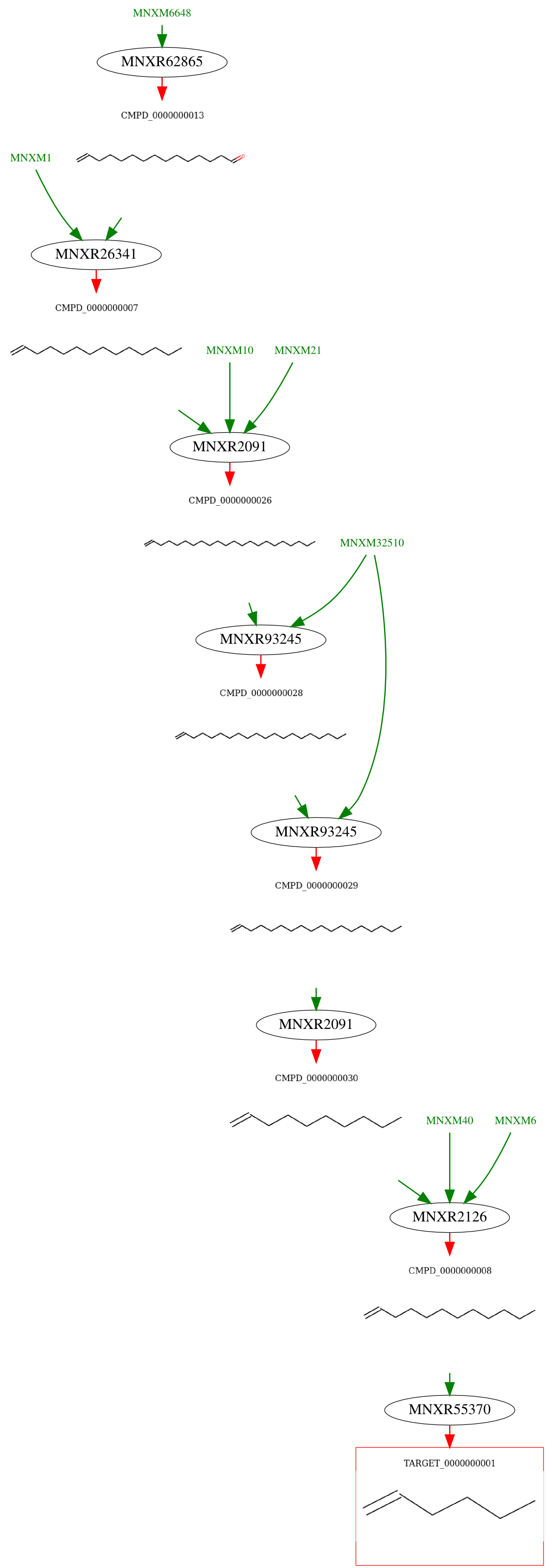

Supplement: Supplementary file 1 — Additional file 1. Monomers maps obtained running Retropath2.0 in section “Virtual screening in the chemical space”. The 17 compounds of the 158 available monomers that can be naturally synthesized and the corresponding synthesis pathways. [file 13321_2017_252_MOESM1_ESM.zip › maps-monomers/butylethylene/path47.png]

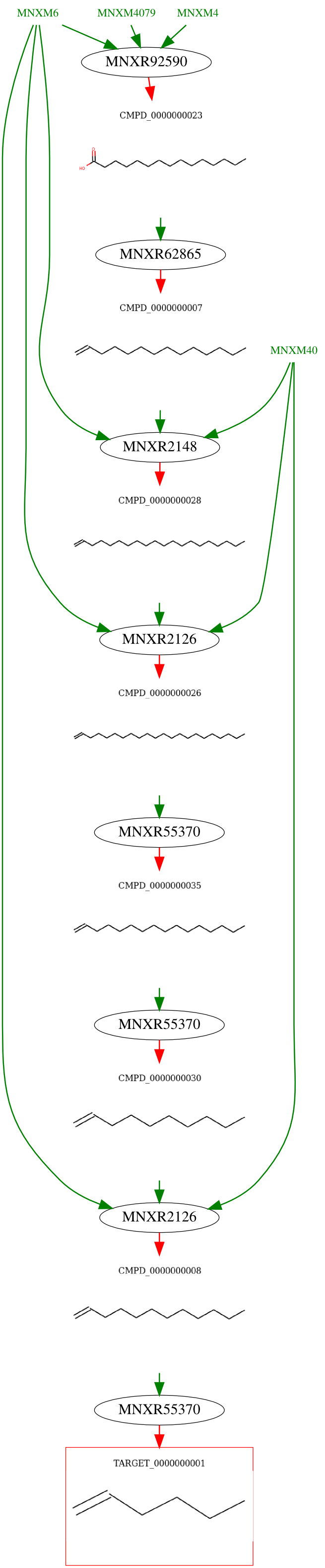

Supplement: Supplementary file 1 — Additional file 1. Monomers maps obtained running Retropath2.0 in section “Virtual screening in the chemical space”. The 17 compounds of the 158 available monomers that can be naturally synthesized and the corresponding synthesis pathways. [file 13321_2017_252_MOESM1_ESM.zip › maps-monomers/butylethylene/path48.png]

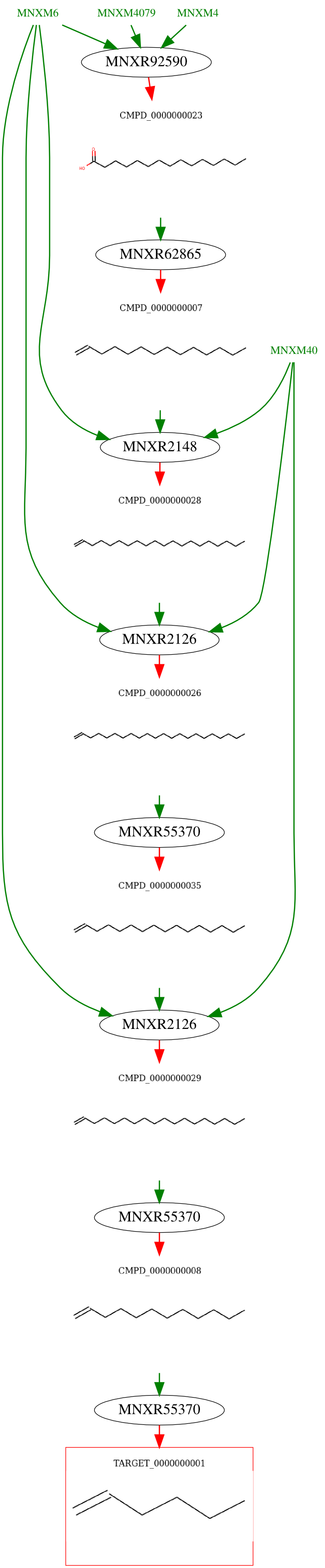

Supplement: Supplementary file 1 — Additional file 1. Monomers maps obtained running Retropath2.0 in section “Virtual screening in the chemical space”. The 17 compounds of the 158 available monomers that can be naturally synthesized and the corresponding synthesis pathways. [file 13321_2017_252_MOESM1_ESM.zip › maps-monomers/butylethylene/path49.png]

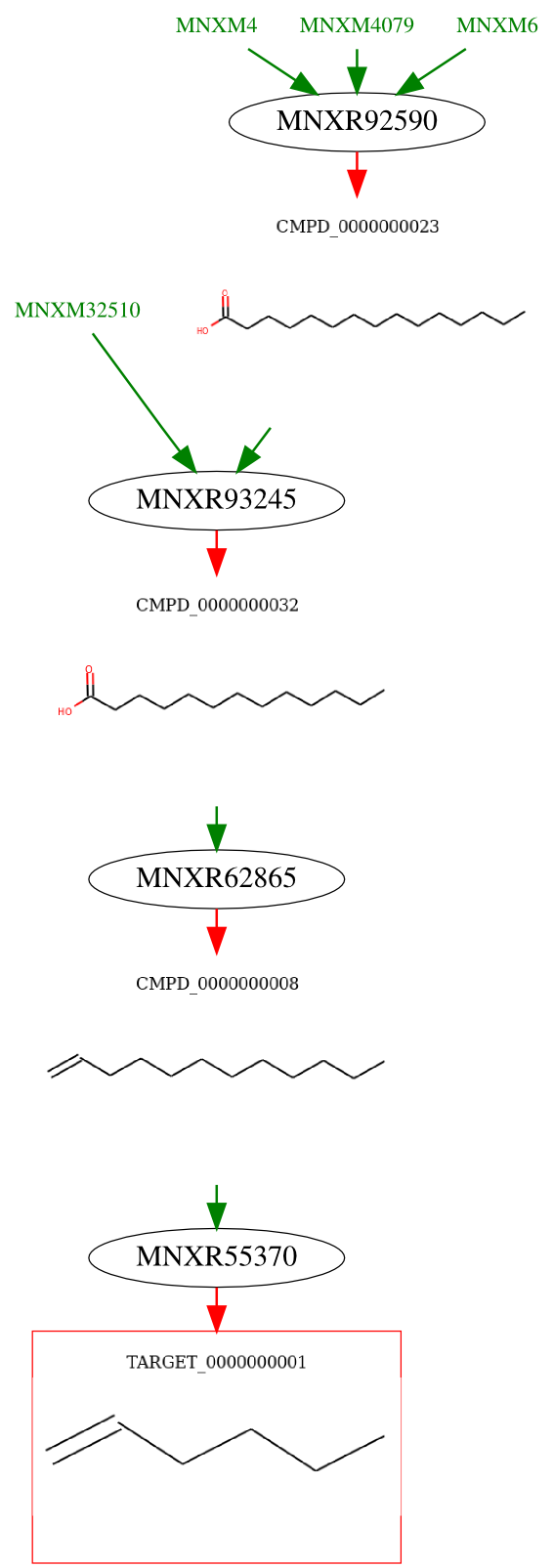

Supplement: Supplementary file 1 — Additional file 1. Monomers maps obtained running Retropath2.0 in section “Virtual screening in the chemical space”. The 17 compounds of the 158 available monomers that can be naturally synthesized and the corresponding synthesis pathways. [file 13321_2017_252_MOESM1_ESM.zip › maps-monomers/butylethylene/path5.png]

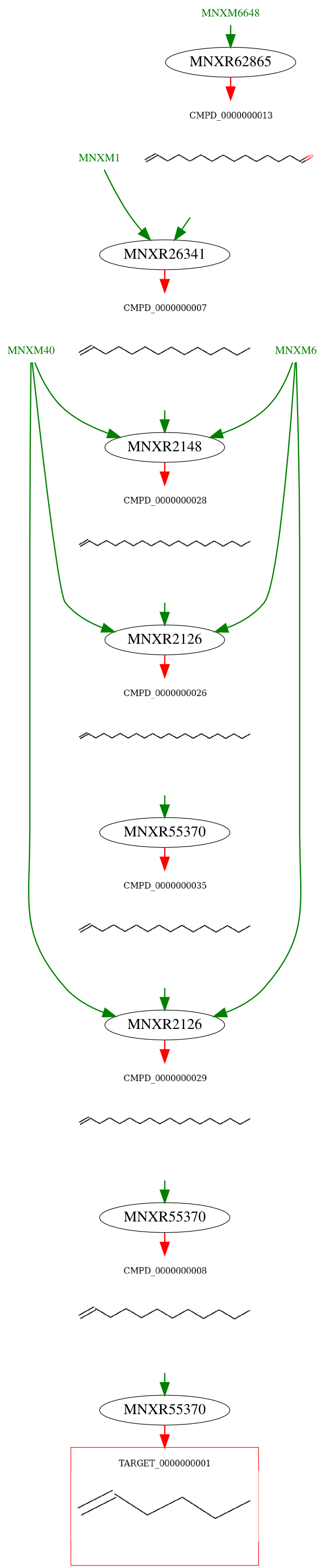

Supplement: Supplementary file 1 — Additional file 1. Monomers maps obtained running Retropath2.0 in section “Virtual screening in the chemical space”. The 17 compounds of the 158 available monomers that can be naturally synthesized and the corresponding synthesis pathways. [file 13321_2017_252_MOESM1_ESM.zip › maps-monomers/butylethylene/path50.png]

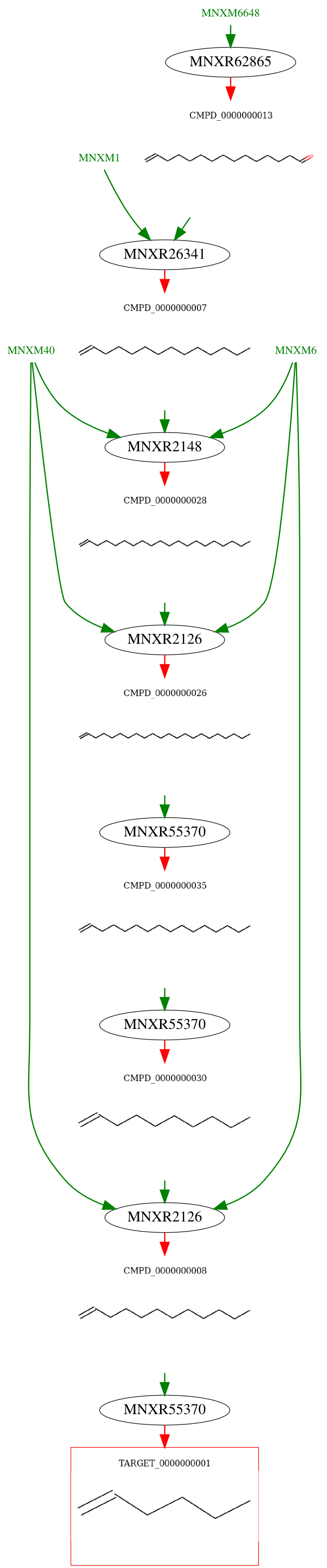

Supplement: Supplementary file 1 — Additional file 1. Monomers maps obtained running Retropath2.0 in section “Virtual screening in the chemical space”. The 17 compounds of the 158 available monomers that can be naturally synthesized and the corresponding synthesis pathways. [file 13321_2017_252_MOESM1_ESM.zip › maps-monomers/butylethylene/path51.png]

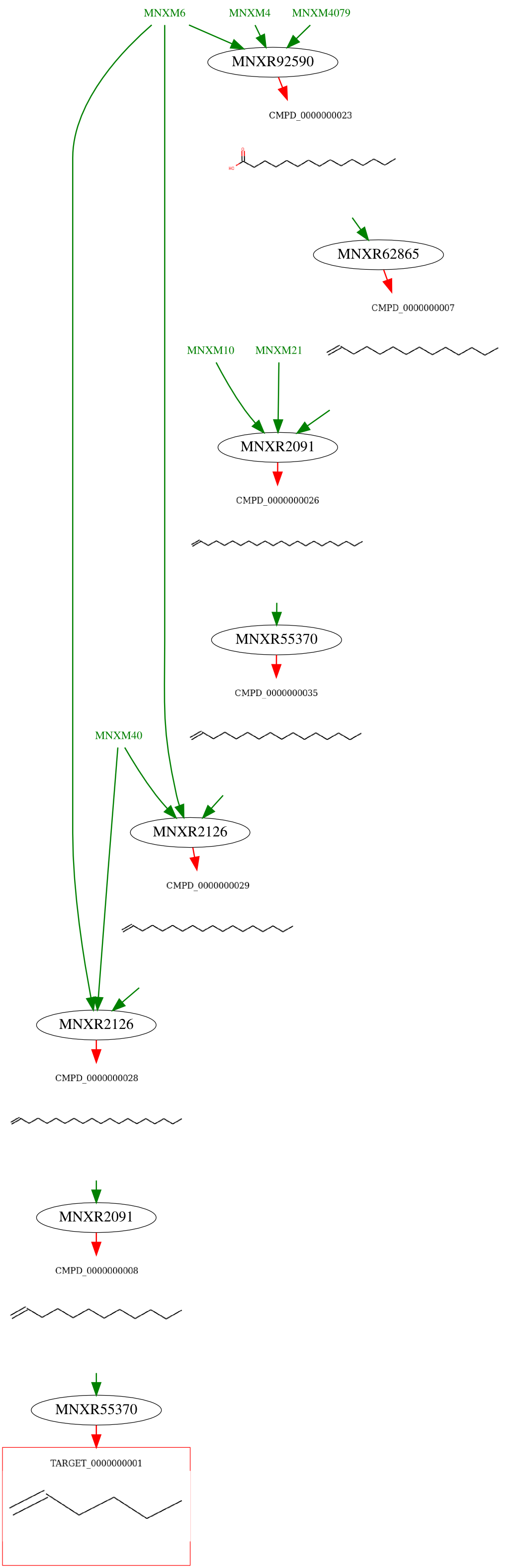

Supplement: Supplementary file 1 — Additional file 1. Monomers maps obtained running Retropath2.0 in section “Virtual screening in the chemical space”. The 17 compounds of the 158 available monomers that can be naturally synthesized and the corresponding synthesis pathways. [file 13321_2017_252_MOESM1_ESM.zip › maps-monomers/butylethylene/path52.png]

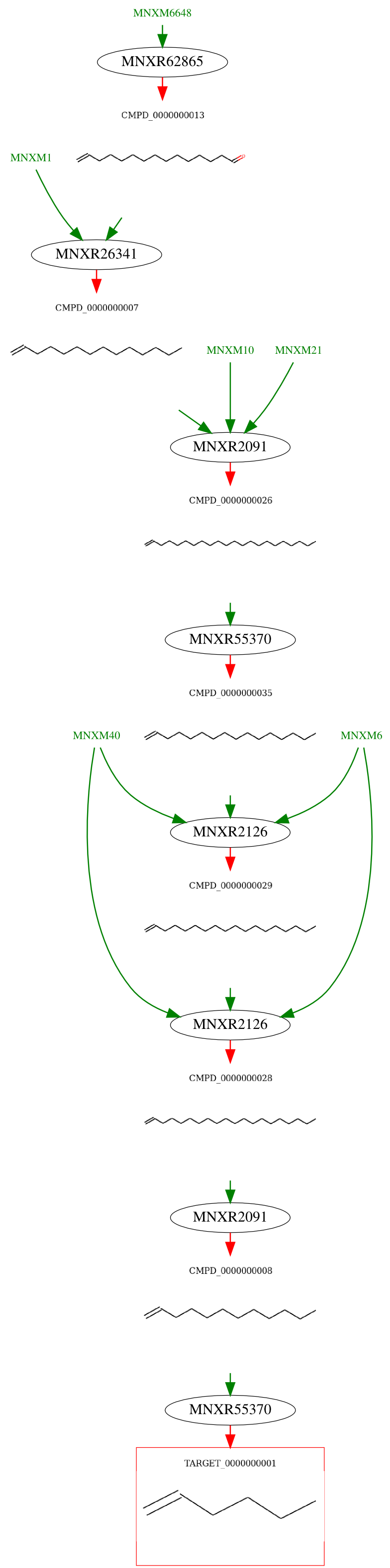

Supplement: Supplementary file 1 — Additional file 1. Monomers maps obtained running Retropath2.0 in section “Virtual screening in the chemical space”. The 17 compounds of the 158 available monomers that can be naturally synthesized and the corresponding synthesis pathways. [file 13321_2017_252_MOESM1_ESM.zip › maps-monomers/butylethylene/path53.png]

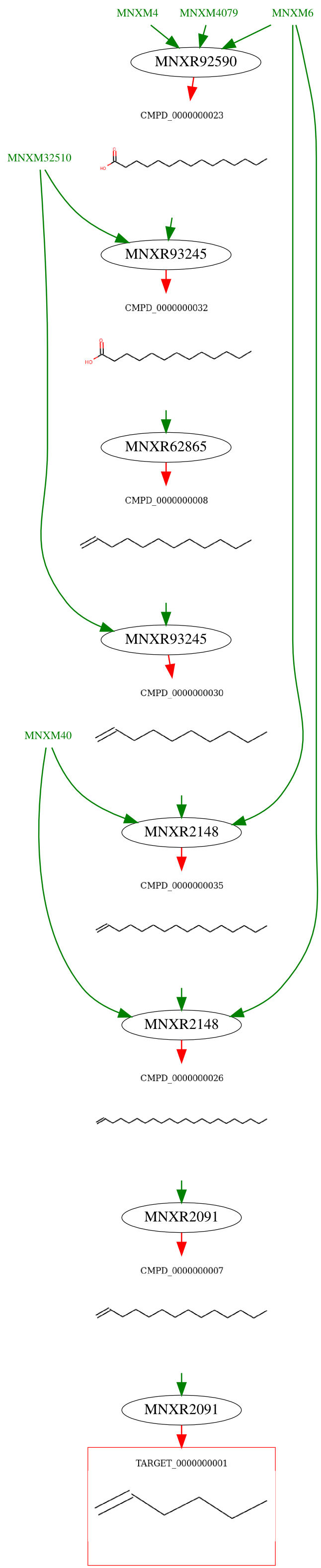

Supplement: Supplementary file 1 — Additional file 1. Monomers maps obtained running Retropath2.0 in section “Virtual screening in the chemical space”. The 17 compounds of the 158 available monomers that can be naturally synthesized and the corresponding synthesis pathways. [file 13321_2017_252_MOESM1_ESM.zip › maps-monomers/butylethylene/path54.png]

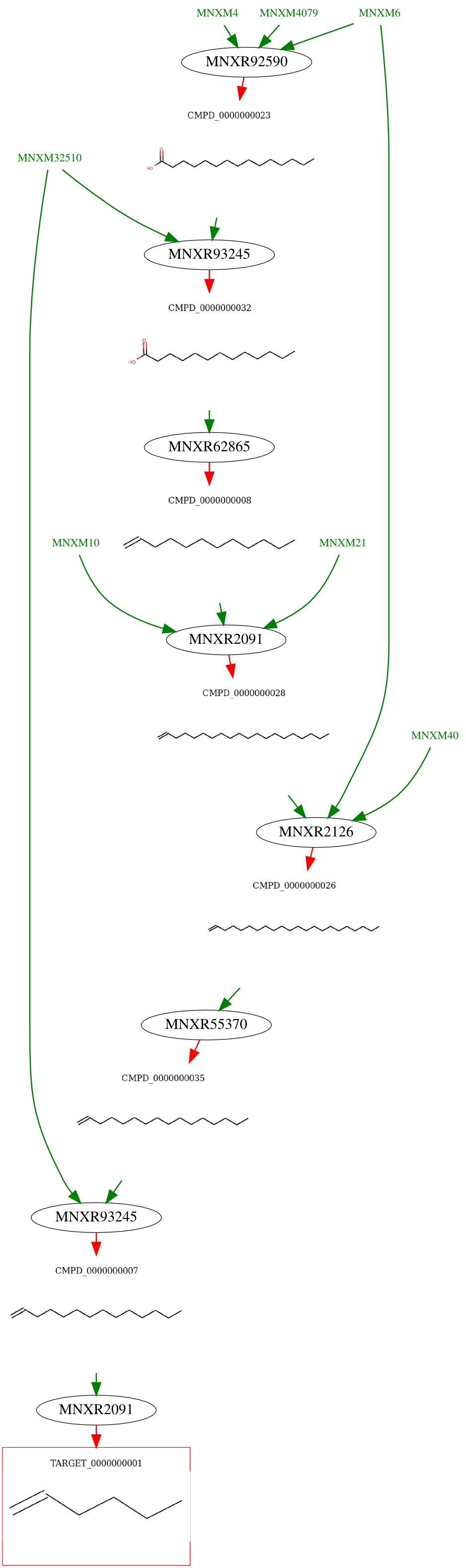

Supplement: Supplementary file 1 — Additional file 1. Monomers maps obtained running Retropath2.0 in section “Virtual screening in the chemical space”. The 17 compounds of the 158 available monomers that can be naturally synthesized and the corresponding synthesis pathways. [file 13321_2017_252_MOESM1_ESM.zip › maps-monomers/butylethylene/path55.png]

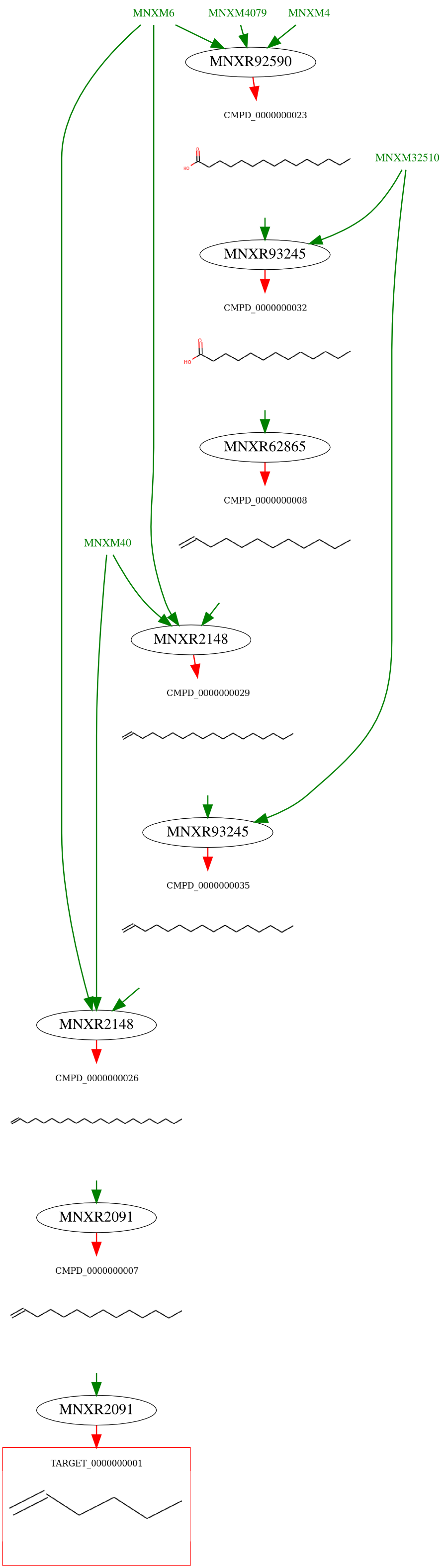

Supplement: Supplementary file 1 — Additional file 1. Monomers maps obtained running Retropath2.0 in section “Virtual screening in the chemical space”. The 17 compounds of the 158 available monomers that can be naturally synthesized and the corresponding synthesis pathways. [file 13321_2017_252_MOESM1_ESM.zip › maps-monomers/butylethylene/path56.png]

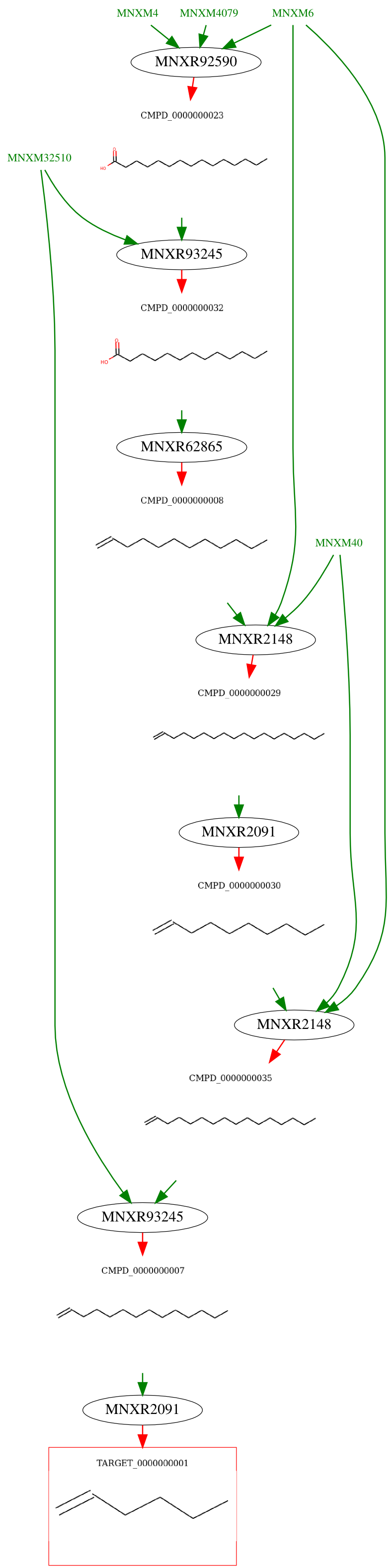

Supplement: Supplementary file 1 — Additional file 1. Monomers maps obtained running Retropath2.0 in section “Virtual screening in the chemical space”. The 17 compounds of the 158 available monomers that can be naturally synthesized and the corresponding synthesis pathways. [file 13321_2017_252_MOESM1_ESM.zip › maps-monomers/butylethylene/path57.png]

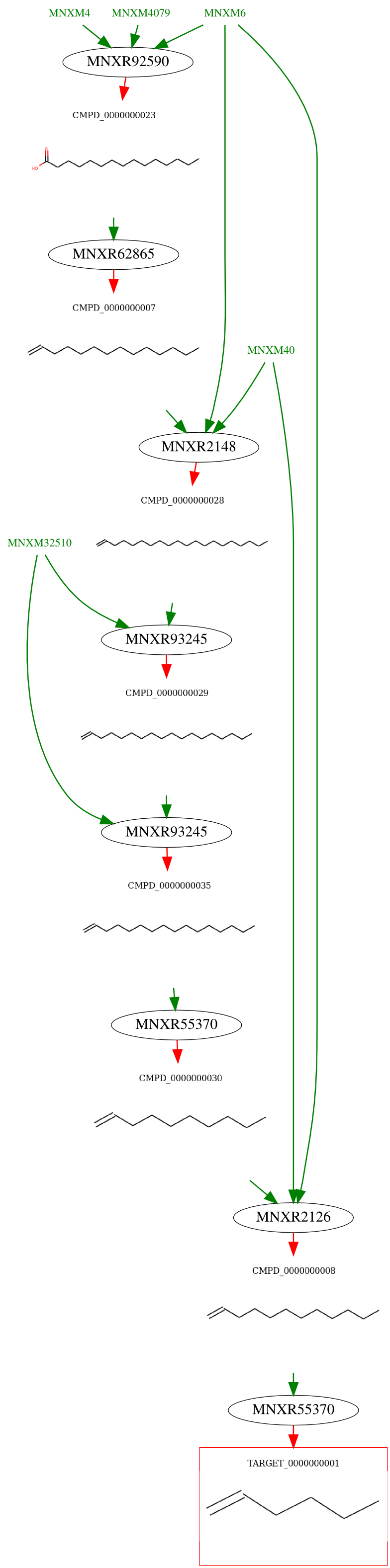

Supplement: Supplementary file 1 — Additional file 1. Monomers maps obtained running Retropath2.0 in section “Virtual screening in the chemical space”. The 17 compounds of the 158 available monomers that can be naturally synthesized and the corresponding synthesis pathways. [file 13321_2017_252_MOESM1_ESM.zip › maps-monomers/butylethylene/path58.png]

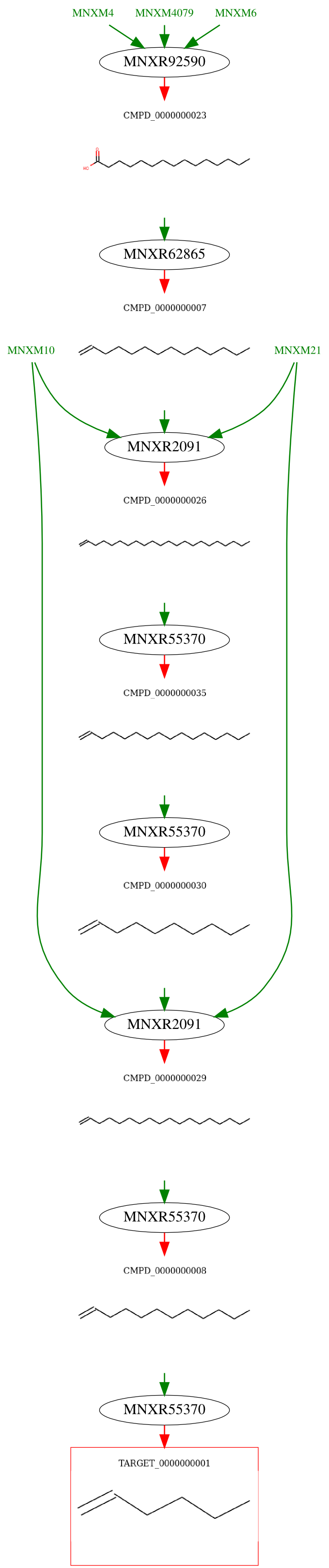

Supplement: Supplementary file 1 — Additional file 1. Monomers maps obtained running Retropath2.0 in section “Virtual screening in the chemical space”. The 17 compounds of the 158 available monomers that can be naturally synthesized and the corresponding synthesis pathways. [file 13321_2017_252_MOESM1_ESM.zip › maps-monomers/butylethylene/path59.png]

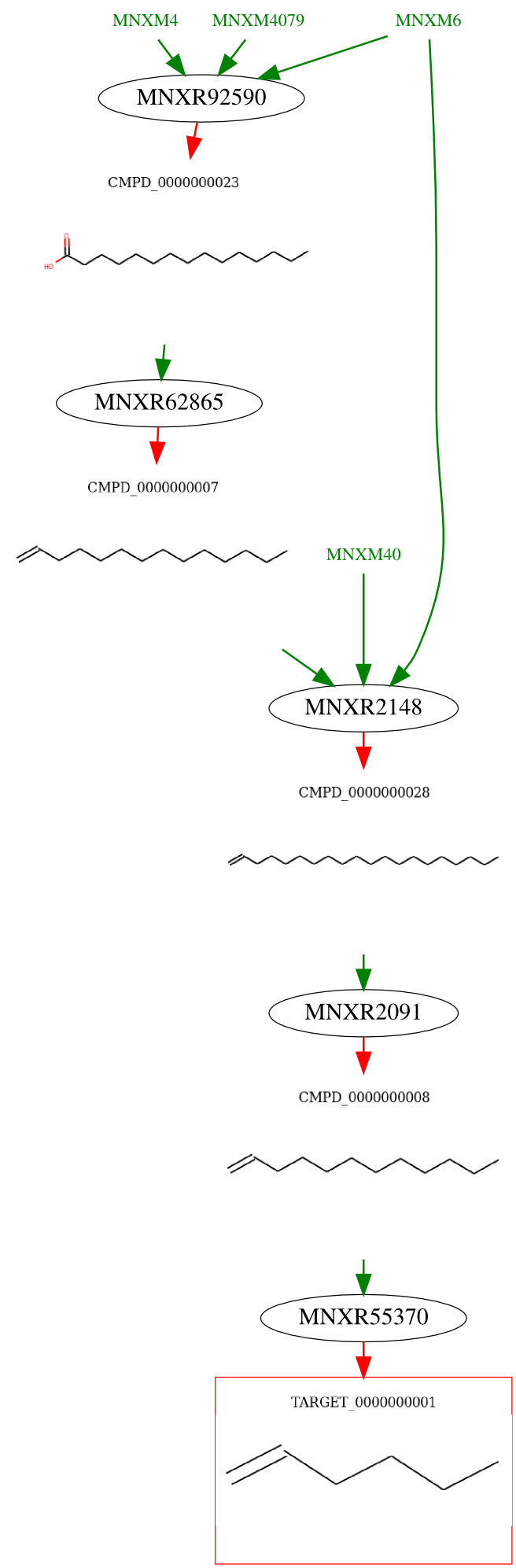

Supplement: Supplementary file 1 — Additional file 1. Monomers maps obtained running Retropath2.0 in section “Virtual screening in the chemical space”. The 17 compounds of the 158 available monomers that can be naturally synthesized and the corresponding synthesis pathways. [file 13321_2017_252_MOESM1_ESM.zip › maps-monomers/butylethylene/path6.png]

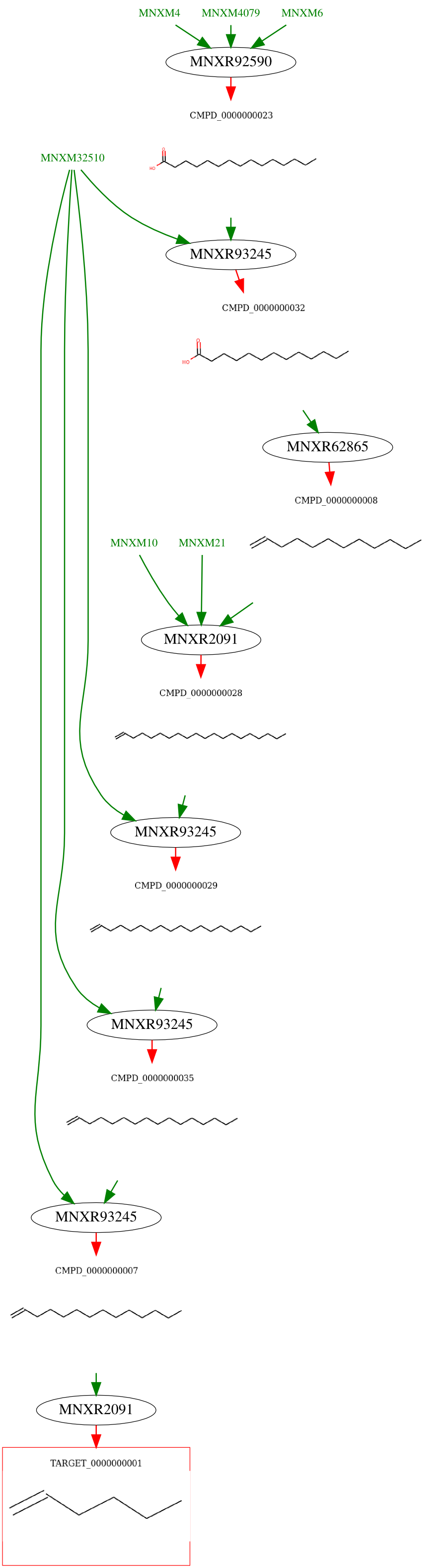

Supplement: Supplementary file 1 — Additional file 1. Monomers maps obtained running Retropath2.0 in section “Virtual screening in the chemical space”. The 17 compounds of the 158 available monomers that can be naturally synthesized and the corresponding synthesis pathways. [file 13321_2017_252_MOESM1_ESM.zip › maps-monomers/butylethylene/path60.png]

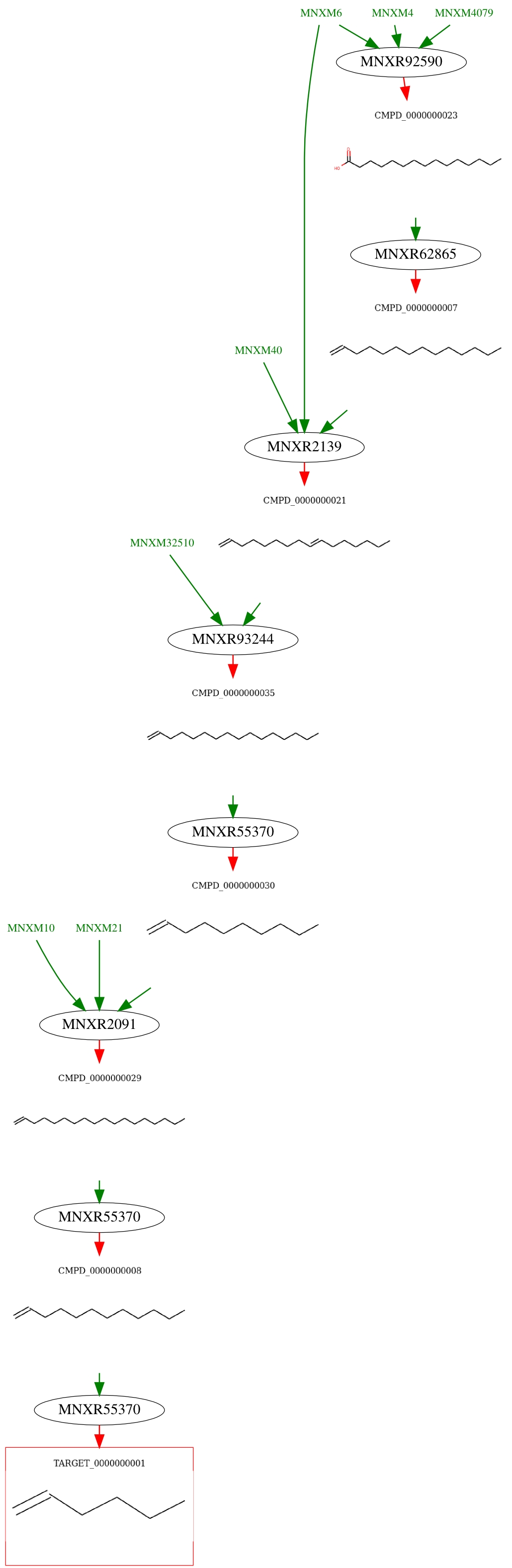

Supplement: Supplementary file 1 — Additional file 1. Monomers maps obtained running Retropath2.0 in section “Virtual screening in the chemical space”. The 17 compounds of the 158 available monomers that can be naturally synthesized and the corresponding synthesis pathways. [file 13321_2017_252_MOESM1_ESM.zip › maps-monomers/butylethylene/path61.png]

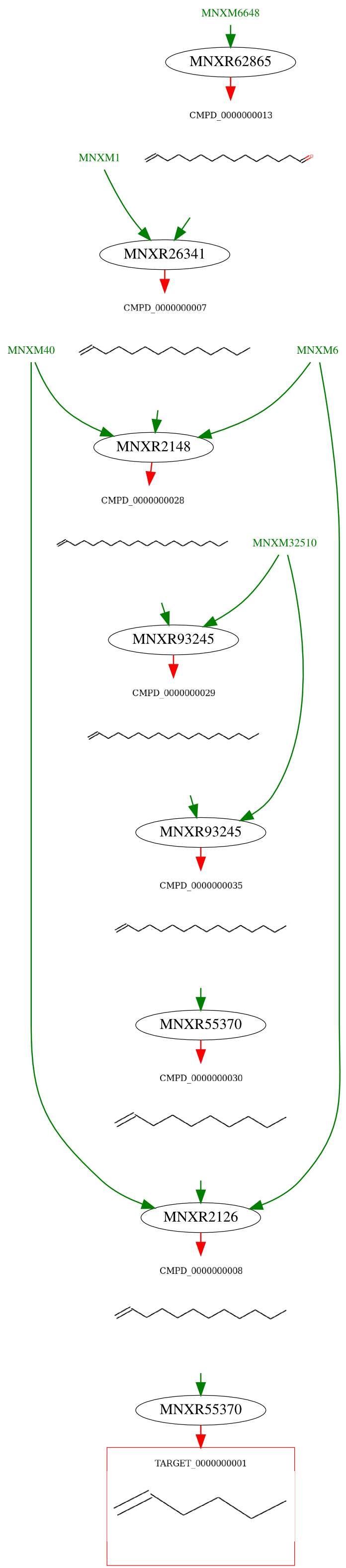

Supplement: Supplementary file 1 — Additional file 1. Monomers maps obtained running Retropath2.0 in section “Virtual screening in the chemical space”. The 17 compounds of the 158 available monomers that can be naturally synthesized and the corresponding synthesis pathways. [file 13321_2017_252_MOESM1_ESM.zip › maps-monomers/butylethylene/path62.png]

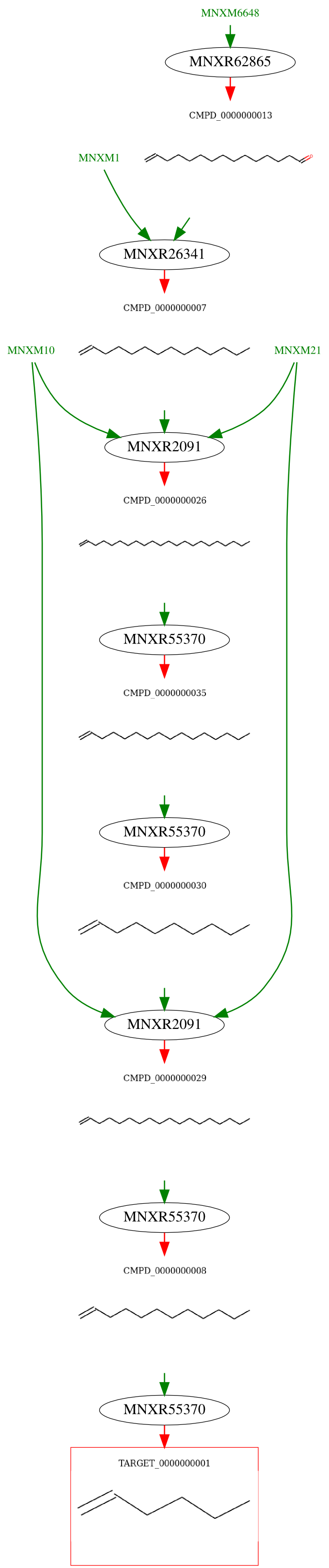

Supplement: Supplementary file 1 — Additional file 1. Monomers maps obtained running Retropath2.0 in section “Virtual screening in the chemical space”. The 17 compounds of the 158 available monomers that can be naturally synthesized and the corresponding synthesis pathways. [file 13321_2017_252_MOESM1_ESM.zip › maps-monomers/butylethylene/path63.png]

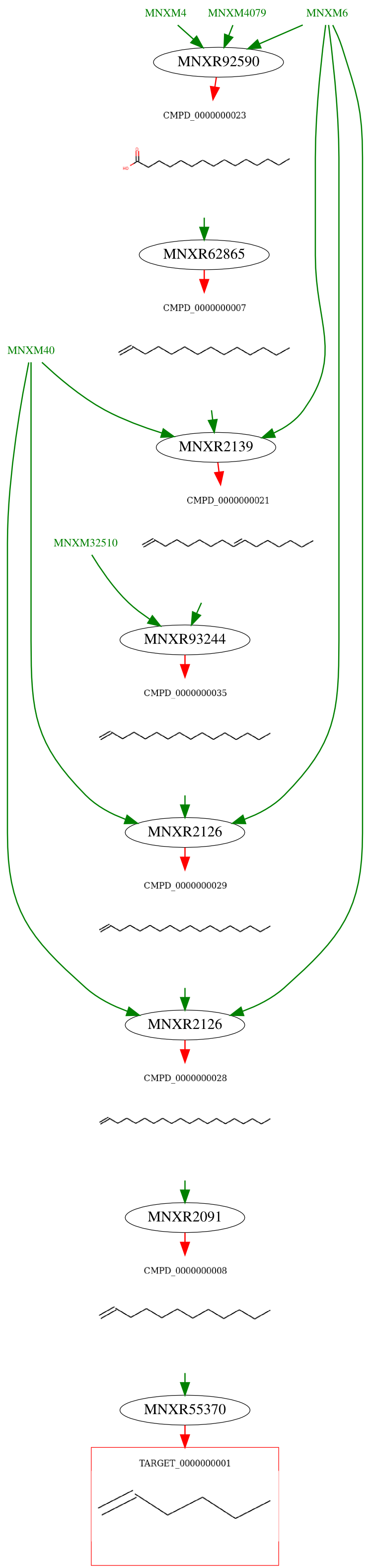

Supplement: Supplementary file 1 — Additional file 1. Monomers maps obtained running Retropath2.0 in section “Virtual screening in the chemical space”. The 17 compounds of the 158 available monomers that can be naturally synthesized and the corresponding synthesis pathways. [file 13321_2017_252_MOESM1_ESM.zip › maps-monomers/butylethylene/path64.png]

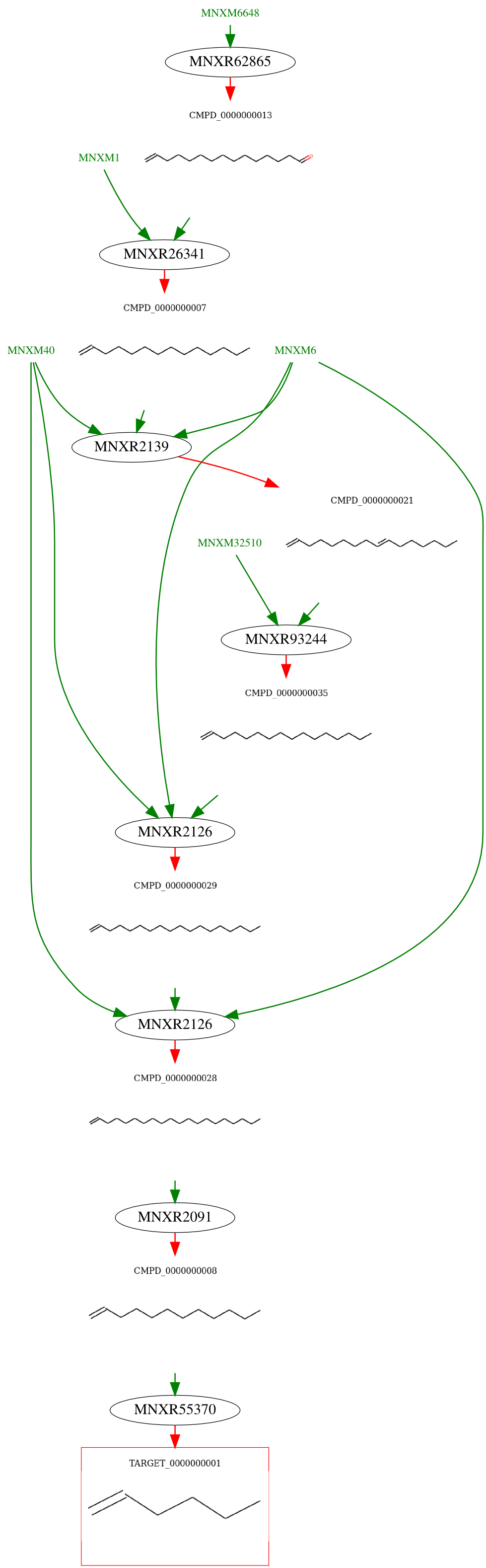

Supplement: Supplementary file 1 — Additional file 1. Monomers maps obtained running Retropath2.0 in section “Virtual screening in the chemical space”. The 17 compounds of the 158 available monomers that can be naturally synthesized and the corresponding synthesis pathways. [file 13321_2017_252_MOESM1_ESM.zip › maps-monomers/butylethylene/path65.png]

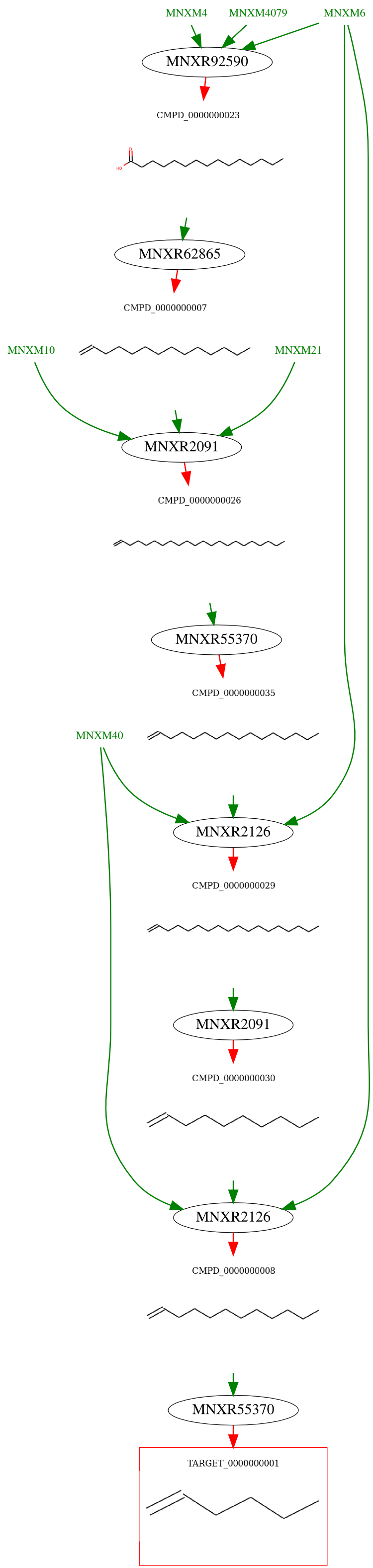

Supplement: Supplementary file 1 — Additional file 1. Monomers maps obtained running Retropath2.0 in section “Virtual screening in the chemical space”. The 17 compounds of the 158 available monomers that can be naturally synthesized and the corresponding synthesis pathways. [file 13321_2017_252_MOESM1_ESM.zip › maps-monomers/butylethylene/path66.png]

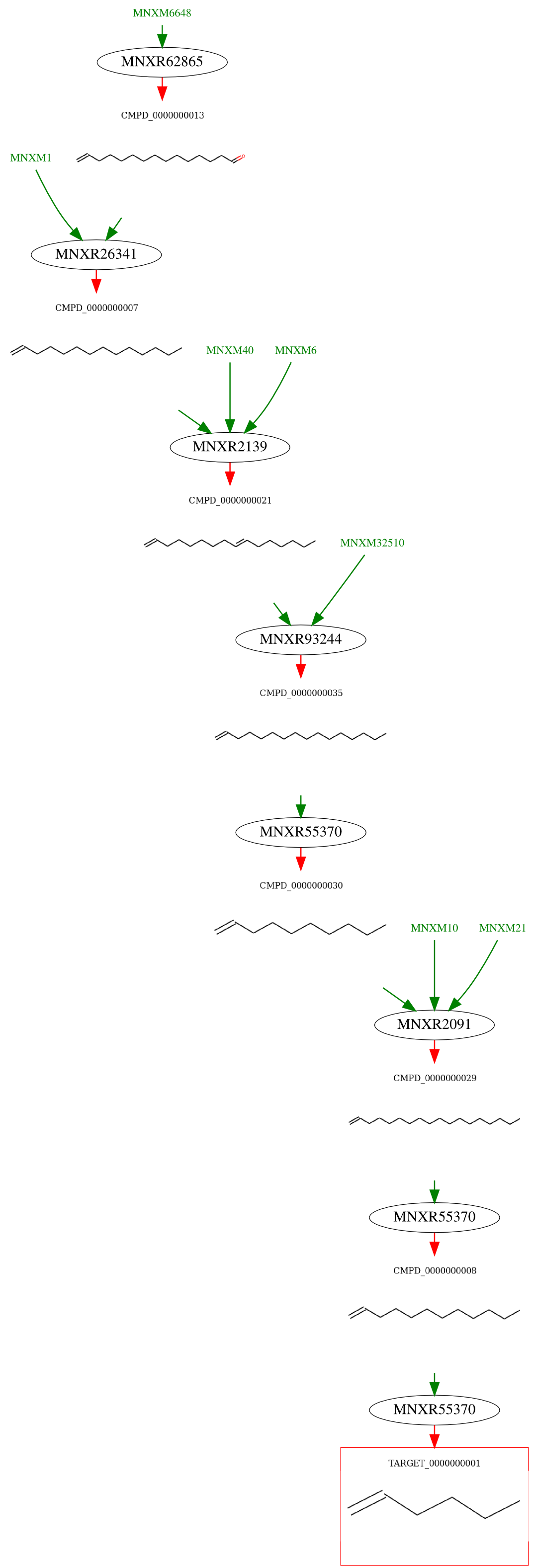

Supplement: Supplementary file 1 — Additional file 1. Monomers maps obtained running Retropath2.0 in section “Virtual screening in the chemical space”. The 17 compounds of the 158 available monomers that can be naturally synthesized and the corresponding synthesis pathways. [file 13321_2017_252_MOESM1_ESM.zip › maps-monomers/butylethylene/path67.png]

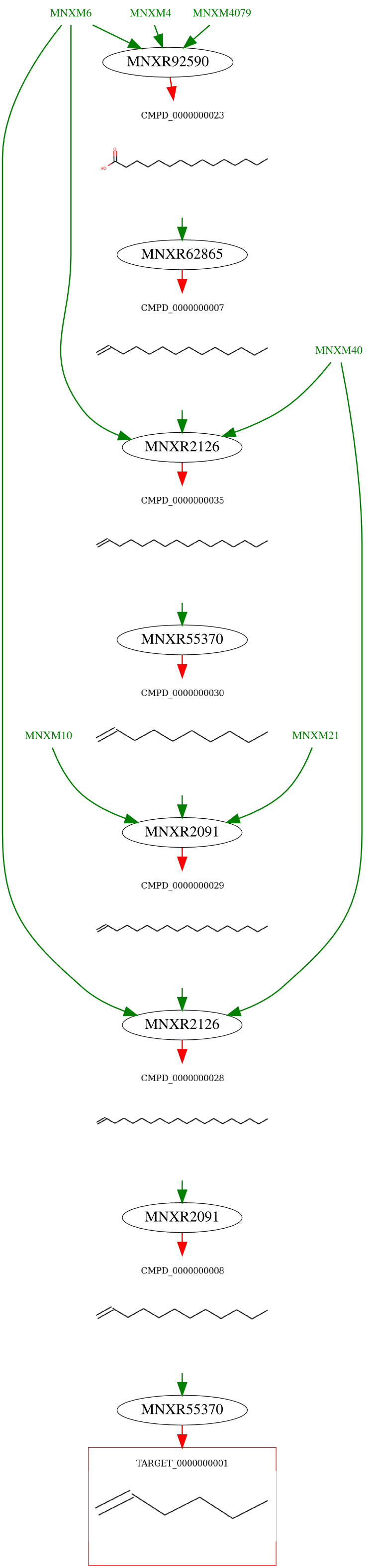

Supplement: Supplementary file 1 — Additional file 1. Monomers maps obtained running Retropath2.0 in section “Virtual screening in the chemical space”. The 17 compounds of the 158 available monomers that can be naturally synthesized and the corresponding synthesis pathways. [file 13321_2017_252_MOESM1_ESM.zip › maps-monomers/butylethylene/path68.png]

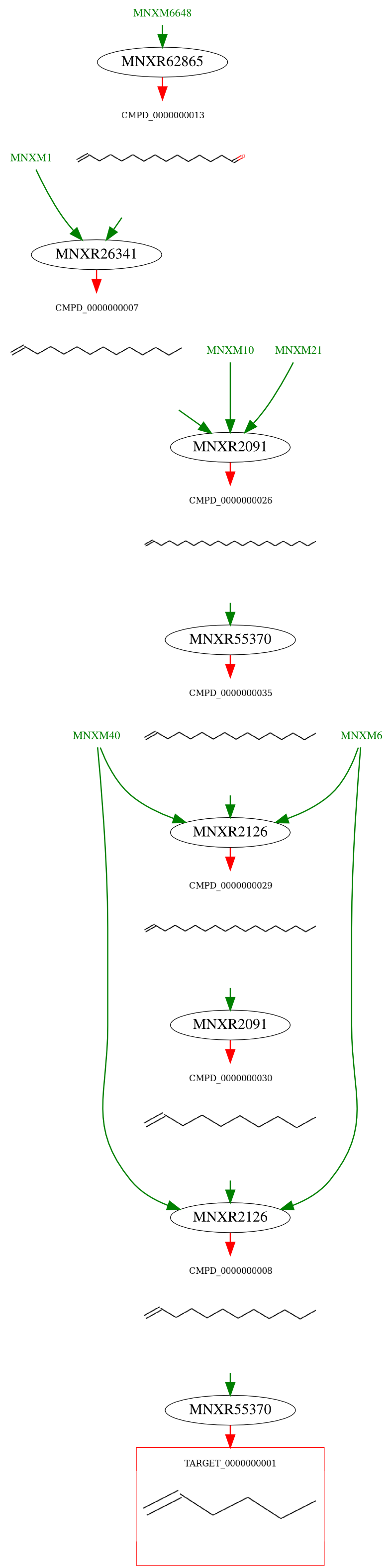

Supplement: Supplementary file 1 — Additional file 1. Monomers maps obtained running Retropath2.0 in section “Virtual screening in the chemical space”. The 17 compounds of the 158 available monomers that can be naturally synthesized and the corresponding synthesis pathways. [file 13321_2017_252_MOESM1_ESM.zip › maps-monomers/butylethylene/path69.png]

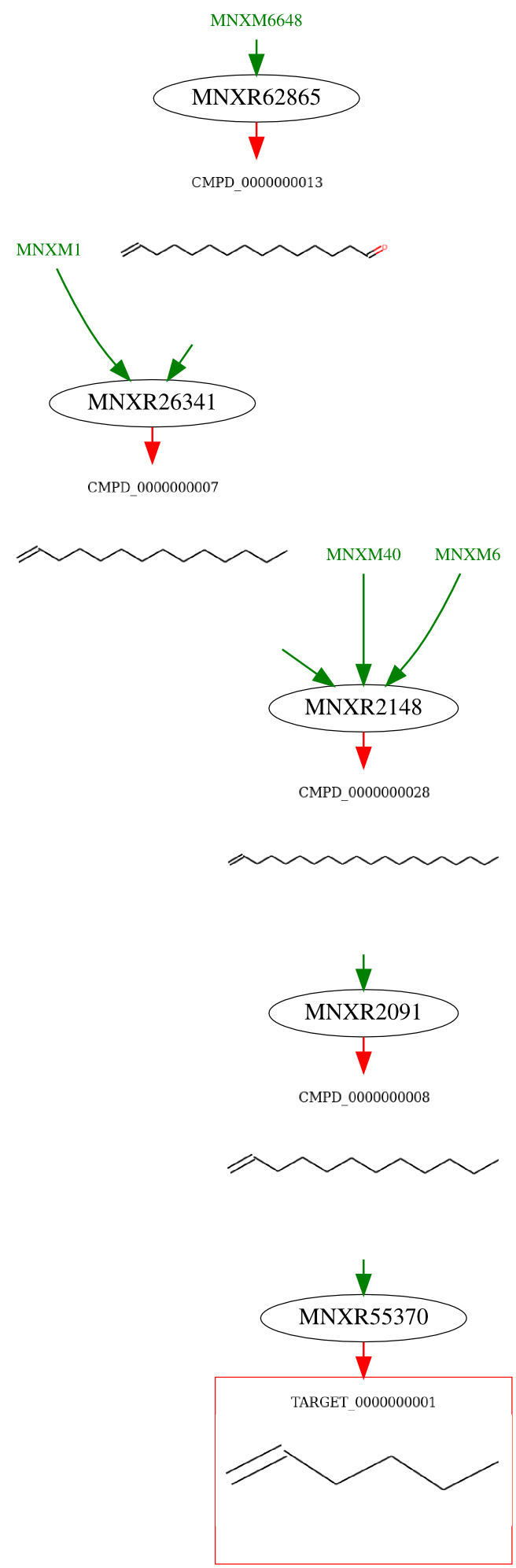

Supplement: Supplementary file 1 — Additional file 1. Monomers maps obtained running Retropath2.0 in section “Virtual screening in the chemical space”. The 17 compounds of the 158 available monomers that can be naturally synthesized and the corresponding synthesis pathways. [file 13321_2017_252_MOESM1_ESM.zip › maps-monomers/butylethylene/path7.png]

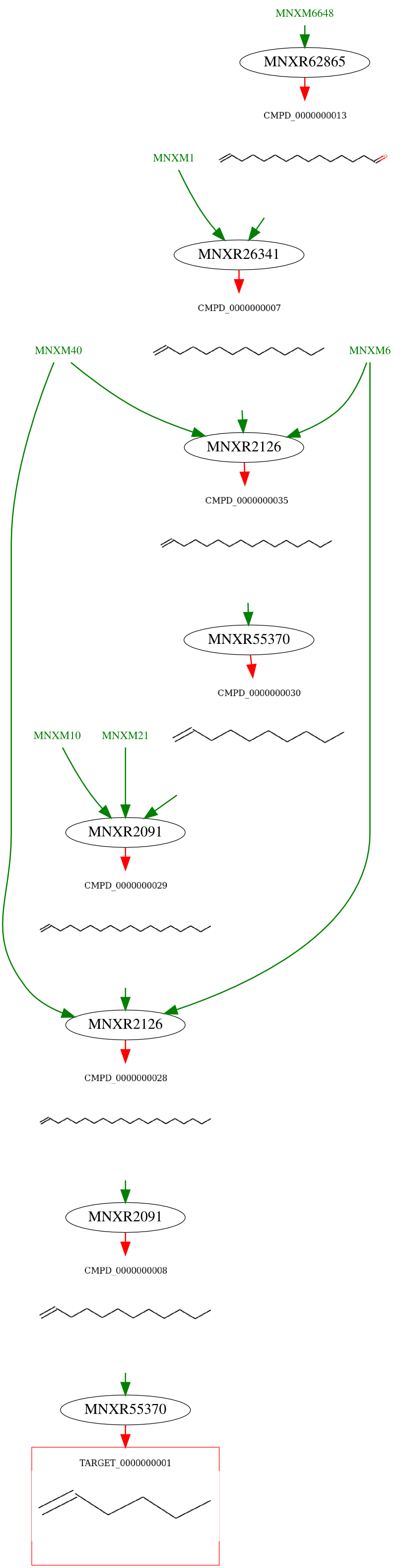

Supplement: Supplementary file 1 — Additional file 1. Monomers maps obtained running Retropath2.0 in section “Virtual screening in the chemical space”. The 17 compounds of the 158 available monomers that can be naturally synthesized and the corresponding synthesis pathways. [file 13321_2017_252_MOESM1_ESM.zip › maps-monomers/butylethylene/path70.png]

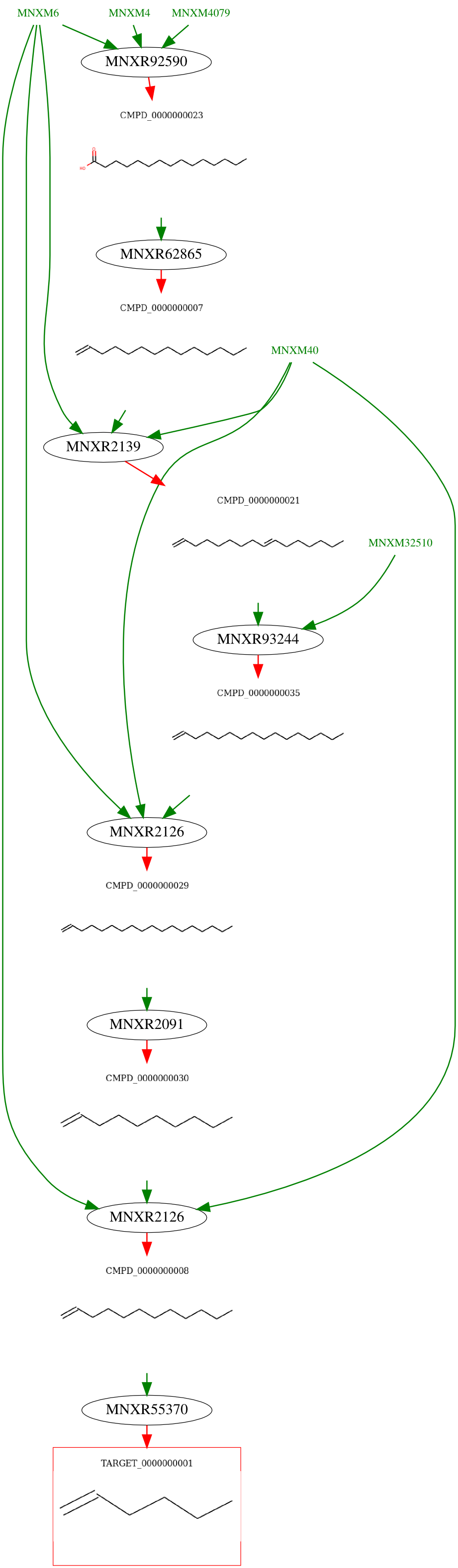

Supplement: Supplementary file 1 — Additional file 1. Monomers maps obtained running Retropath2.0 in section “Virtual screening in the chemical space”. The 17 compounds of the 158 available monomers that can be naturally synthesized and the corresponding synthesis pathways. [file 13321_2017_252_MOESM1_ESM.zip › maps-monomers/butylethylene/path71.png]

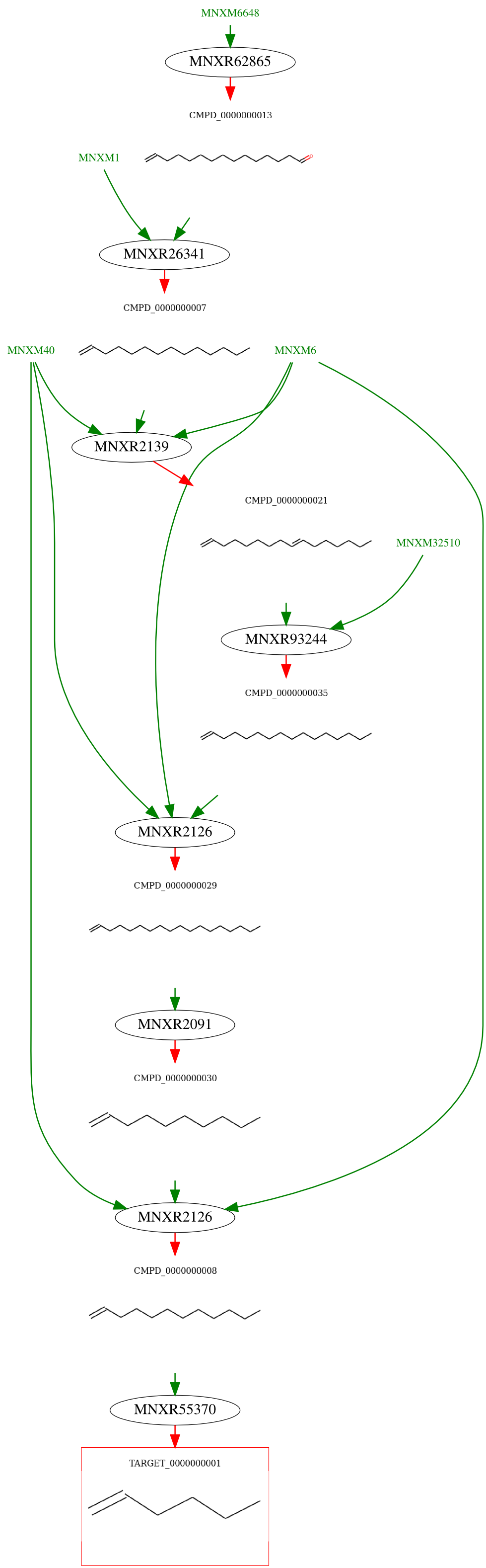

Supplement: Supplementary file 1 — Additional file 1. Monomers maps obtained running Retropath2.0 in section “Virtual screening in the chemical space”. The 17 compounds of the 158 available monomers that can be naturally synthesized and the corresponding synthesis pathways. [file 13321_2017_252_MOESM1_ESM.zip › maps-monomers/butylethylene/path72.png]

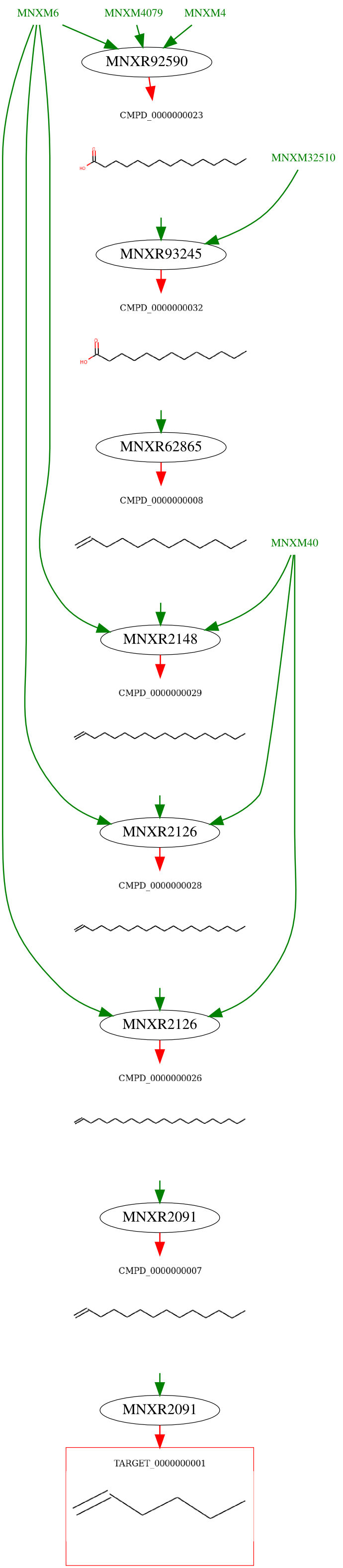

Supplement: Supplementary file 1 — Additional file 1. Monomers maps obtained running Retropath2.0 in section “Virtual screening in the chemical space”. The 17 compounds of the 158 available monomers that can be naturally synthesized and the corresponding synthesis pathways. [file 13321_2017_252_MOESM1_ESM.zip › maps-monomers/butylethylene/path73.png]

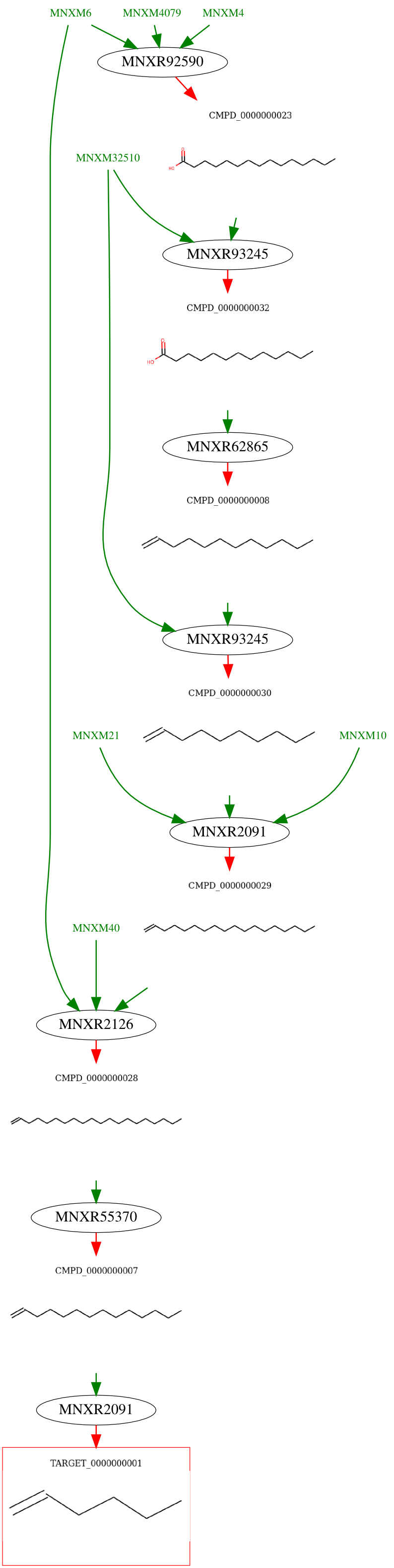

Supplement: Supplementary file 1 — Additional file 1. Monomers maps obtained running Retropath2.0 in section “Virtual screening in the chemical space”. The 17 compounds of the 158 available monomers that can be naturally synthesized and the corresponding synthesis pathways. [file 13321_2017_252_MOESM1_ESM.zip › maps-monomers/butylethylene/path74.png]

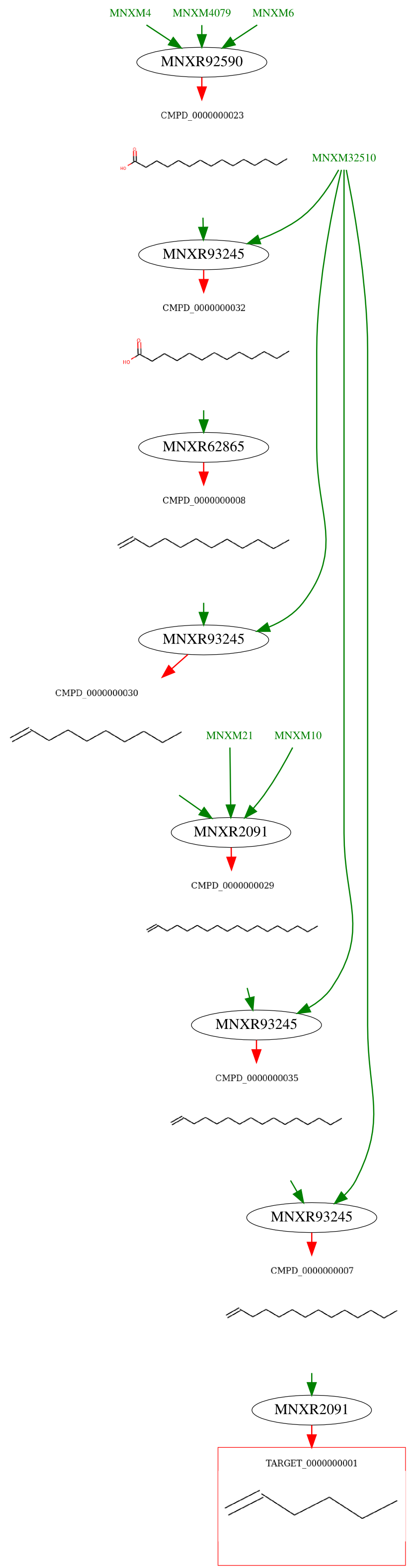

Supplement: Supplementary file 1 — Additional file 1. Monomers maps obtained running Retropath2.0 in section “Virtual screening in the chemical space”. The 17 compounds of the 158 available monomers that can be naturally synthesized and the corresponding synthesis pathways. [file 13321_2017_252_MOESM1_ESM.zip › maps-monomers/butylethylene/path75.png]

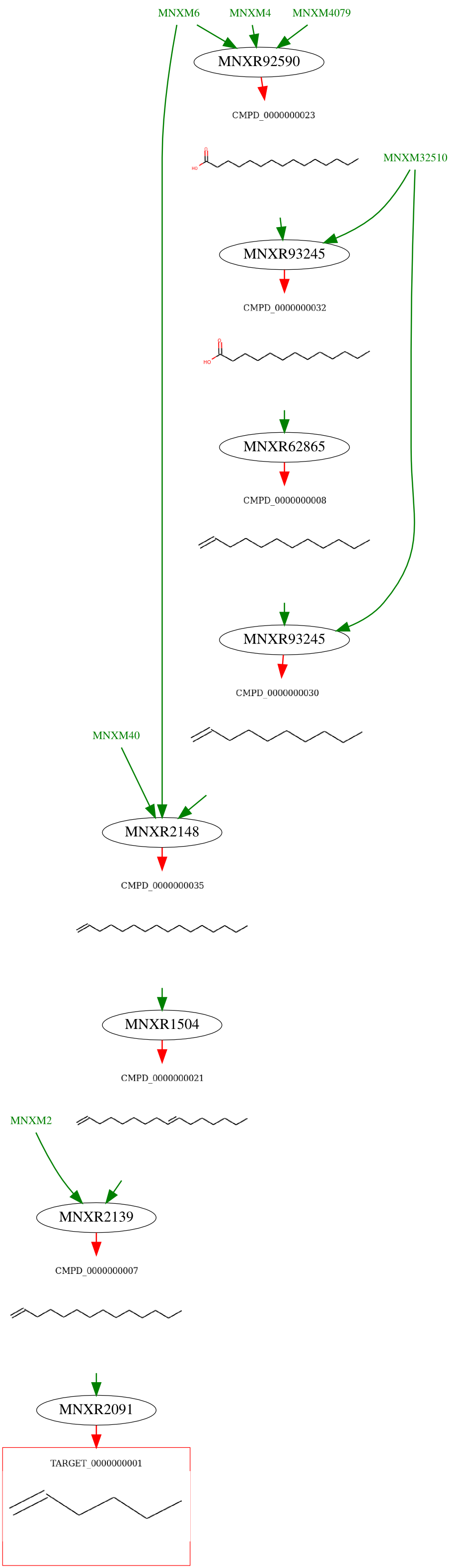

Supplement: Supplementary file 1 — Additional file 1. Monomers maps obtained running Retropath2.0 in section “Virtual screening in the chemical space”. The 17 compounds of the 158 available monomers that can be naturally synthesized and the corresponding synthesis pathways. [file 13321_2017_252_MOESM1_ESM.zip › maps-monomers/butylethylene/path76.png]

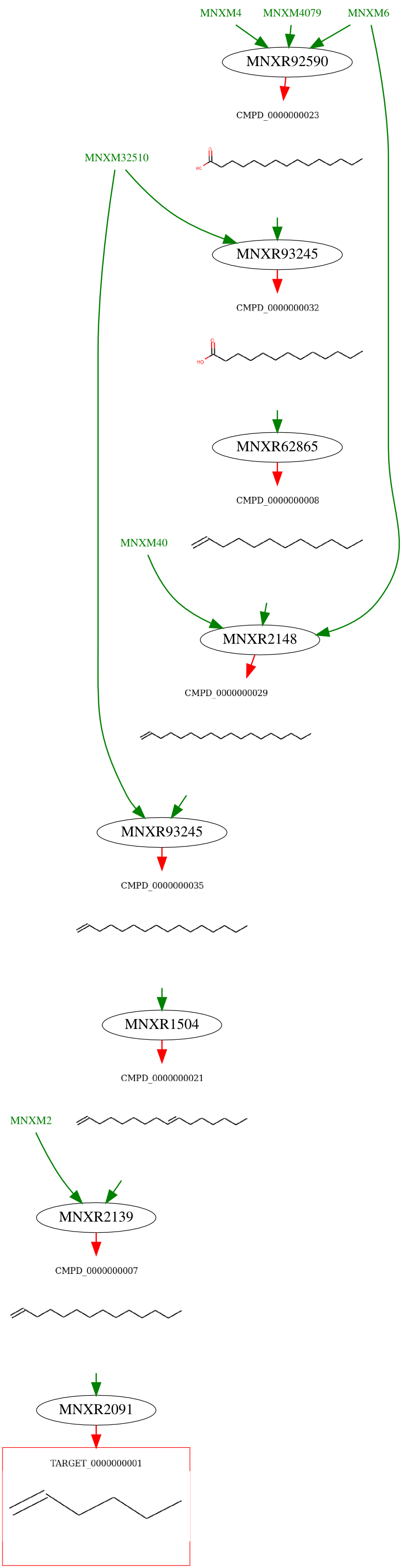

Supplement: Supplementary file 1 — Additional file 1. Monomers maps obtained running Retropath2.0 in section “Virtual screening in the chemical space”. The 17 compounds of the 158 available monomers that can be naturally synthesized and the corresponding synthesis pathways. [file 13321_2017_252_MOESM1_ESM.zip › maps-monomers/butylethylene/path77.png]

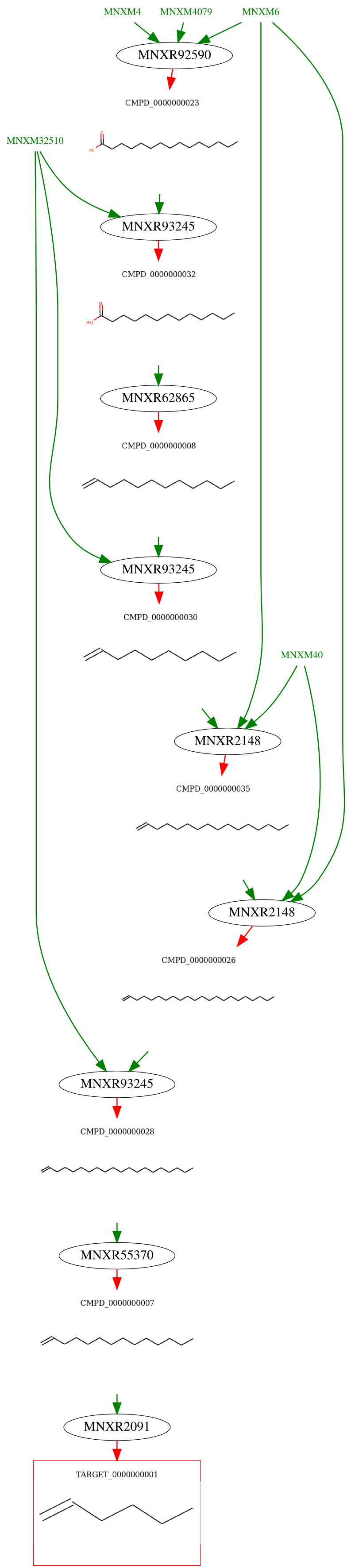

Supplement: Supplementary file 1 — Additional file 1. Monomers maps obtained running Retropath2.0 in section “Virtual screening in the chemical space”. The 17 compounds of the 158 available monomers that can be naturally synthesized and the corresponding synthesis pathways. [file 13321_2017_252_MOESM1_ESM.zip › maps-monomers/butylethylene/path78.png]

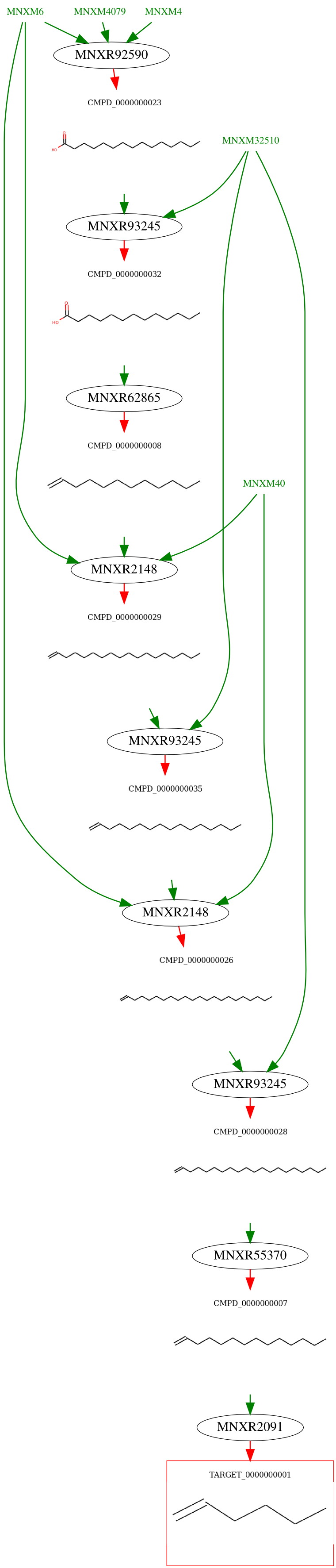

Supplement: Supplementary file 1 — Additional file 1. Monomers maps obtained running Retropath2.0 in section “Virtual screening in the chemical space”. The 17 compounds of the 158 available monomers that can be naturally synthesized and the corresponding synthesis pathways. [file 13321_2017_252_MOESM1_ESM.zip › maps-monomers/butylethylene/path79.png]

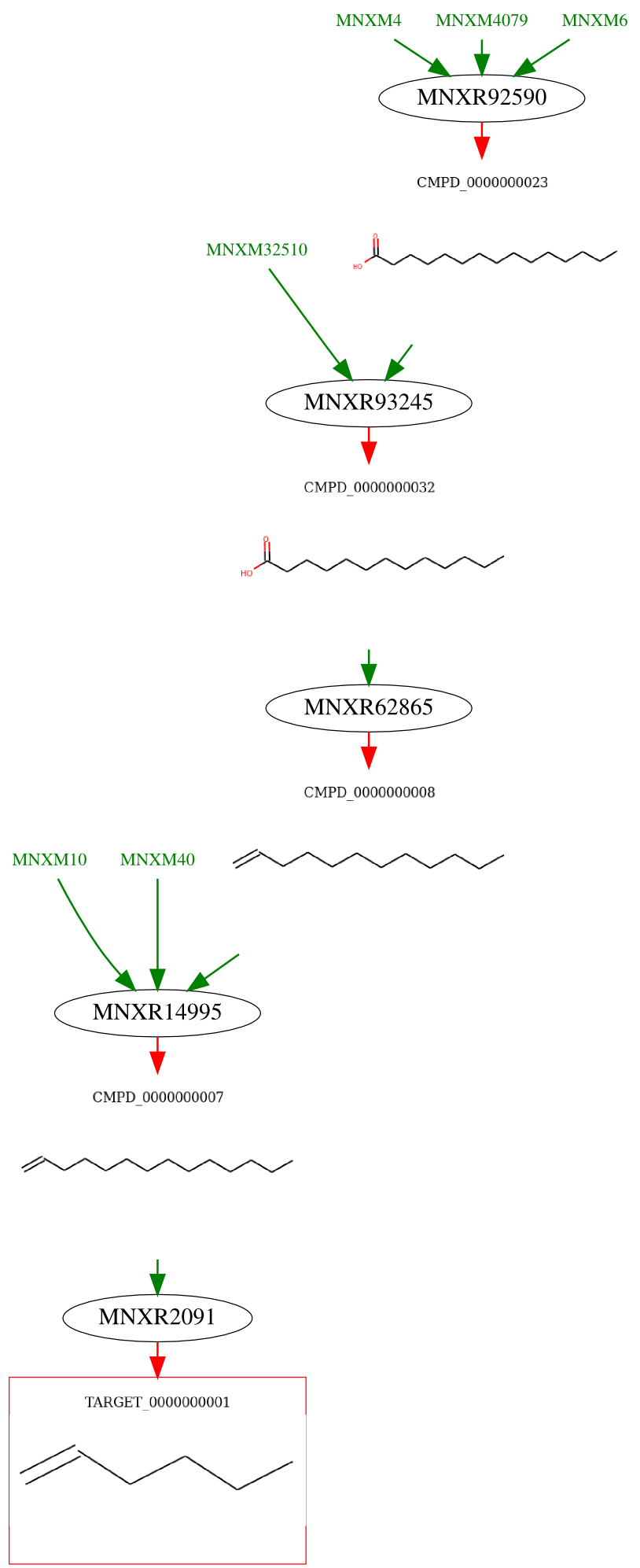

Supplement: Supplementary file 1 — Additional file 1. Monomers maps obtained running Retropath2.0 in section “Virtual screening in the chemical space”. The 17 compounds of the 158 available monomers that can be naturally synthesized and the corresponding synthesis pathways. [file 13321_2017_252_MOESM1_ESM.zip › maps-monomers/butylethylene/path8.png]

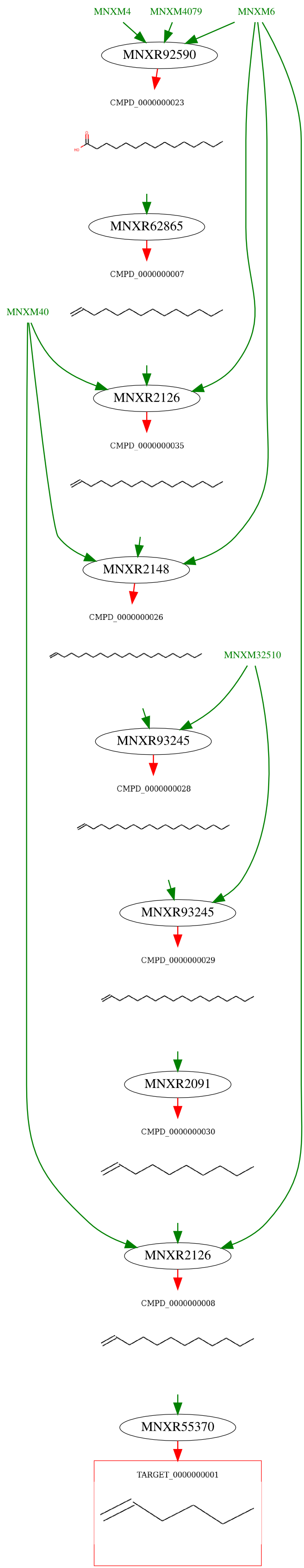

Supplement: Supplementary file 1 — Additional file 1. Monomers maps obtained running Retropath2.0 in section “Virtual screening in the chemical space”. The 17 compounds of the 158 available monomers that can be naturally synthesized and the corresponding synthesis pathways. [file 13321_2017_252_MOESM1_ESM.zip › maps-monomers/butylethylene/path80.png]

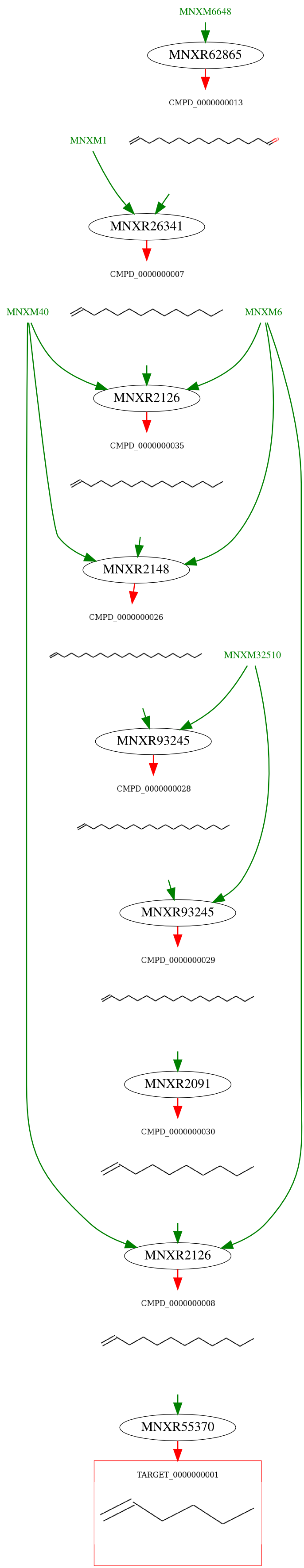

Supplement: Supplementary file 1 — Additional file 1. Monomers maps obtained running Retropath2.0 in section “Virtual screening in the chemical space”. The 17 compounds of the 158 available monomers that can be naturally synthesized and the corresponding synthesis pathways. [file 13321_2017_252_MOESM1_ESM.zip › maps-monomers/butylethylene/path81.png]
