# Supplementary material for: Molecular structures enumeration and virtual screening in the chemical space with RetroPath2.0
Source: J Cheminform. 2017 Dec 19;9:64. doi: 10.1186/s13321-017-0252-9 (PMC5736515; doi:10.1186/s13321-017-0252-9)
Supplement: Supplementary file 2 — Additional file 2. Figure S1 represents the execution time of the tested softwares for alkane enumeration. Figure S2 represents the reduced set of transformation rules excluding triple bounds. Figure S3 represents the distributions of predicted T g values for enumerated isomers and for isomers found in PubChem with varying Tanimoto threshold. [file 13321_2017_252_MOESM2_ESM.docx]

**Molecular structures enumeration and virtual screening in the chemical space with RetroPath2.0**

Mathilde Koch^1^, Thomas Duigou^1^, Pablo Carbonell^2^, Jean-Loup Faulon^1,2,3,*^

1 Micalis Institute, INRA, AgroParisTech, Université Paris-Saclay, 78350 Jouy-en-Josas, France

2 SYNBIOCHEM Centre, Manchester Institute of Biotechnology, University of Manchester, Manchester, UK

3 CNRS-UMR8030 / Laboratoire iSSB, Université Paris-Saclay, Évry 91000, France

*: Corresponding author

Mathilde Koch, Micalis, Institut National de la Recherche Agronomique, Domaine de Vilvert, 78352 Jouy-en-Josas, France; E-mail address: [mathilde.koch@inra.fr](mailto:mathilde.koch@inra.fr)

Thomas Duigou, Micalis, Institut National de la Recherche Agronomique, Domaine de Vilvert, 78352 Jouy-en-Josas, France; E-mail address: [thomas.duigou@inra.fr](mailto:thomas.duigou@inra.fr)

Pablo Carbonell, SYNBIOCHEM Centre, Manchester Institute of Biotechnology, University of Manchester, 131 Princess Street Manchester M1 7DN, UK; E-mail address: pablo.carbonell@manchester.ac.uk

Jean-Loup Faulon, Micalis, Institut National de la Recherche Agronomique, Domaine de Vilvert, 78352 Jouy-en-Josas, France; E-mail address: [jean-loup.faulon@inra.fr](mailto:jean-loup.faulon@inra.fr)


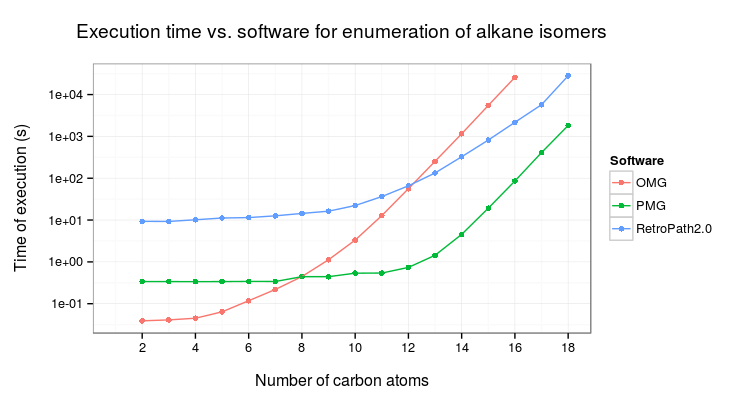


**Figure S1 :** Execution time for each tested software on the enumeration of alkane isomers.


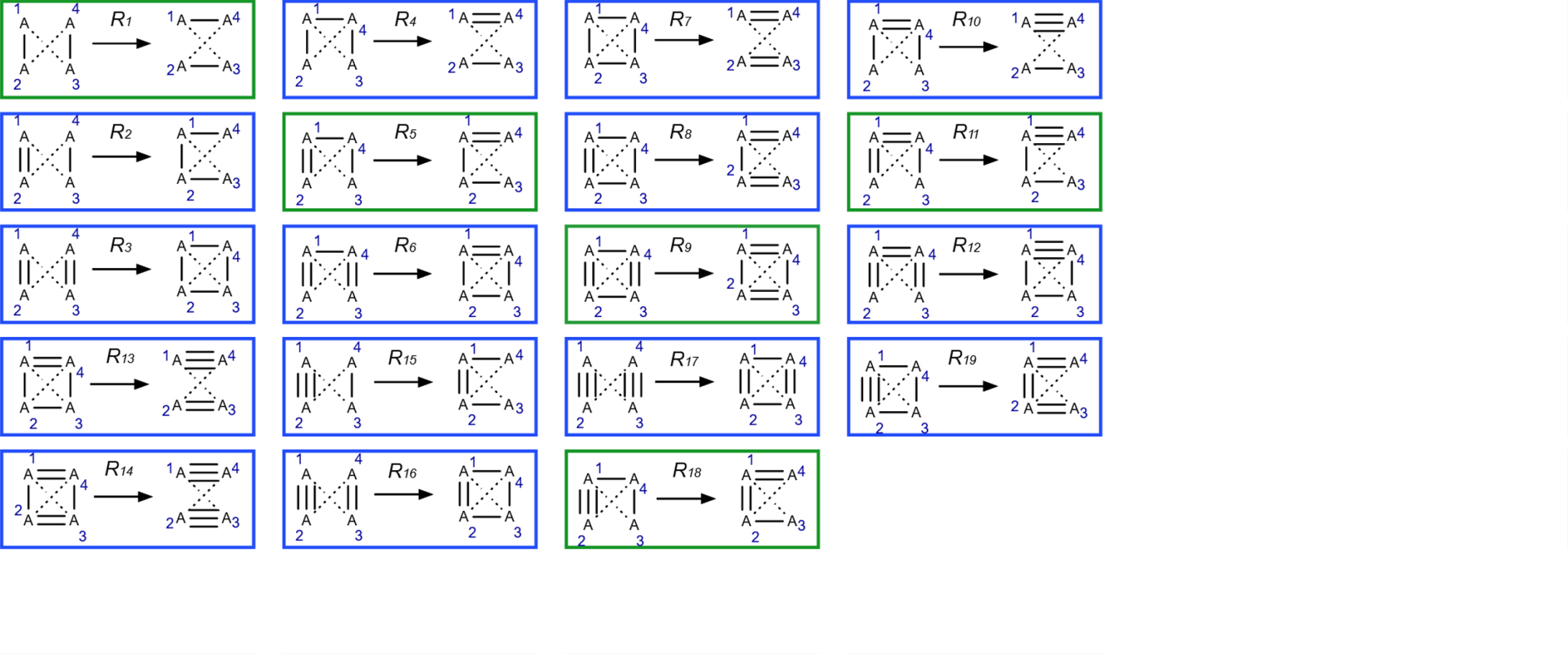


**Figure S2.** Isomer transformation rule set. Reduced set excluding triple bonds. Reactions in green move bonds around without creating or deleting cycles. Reactions in blue change bond order by creating or deleting at least one cycle. To each reaction corresponds a reverse reaction. The reverse reaction of R_1_ is R_1_, for R_2_ it is R_4_, for R_3_ : R_7_, for R_5_: R_5_, for R_6_ : R_8_ and the reverse reaction of R_9_ is R_9_. The bond order a_13_ and a_24_ can take any value from 0 to 3.


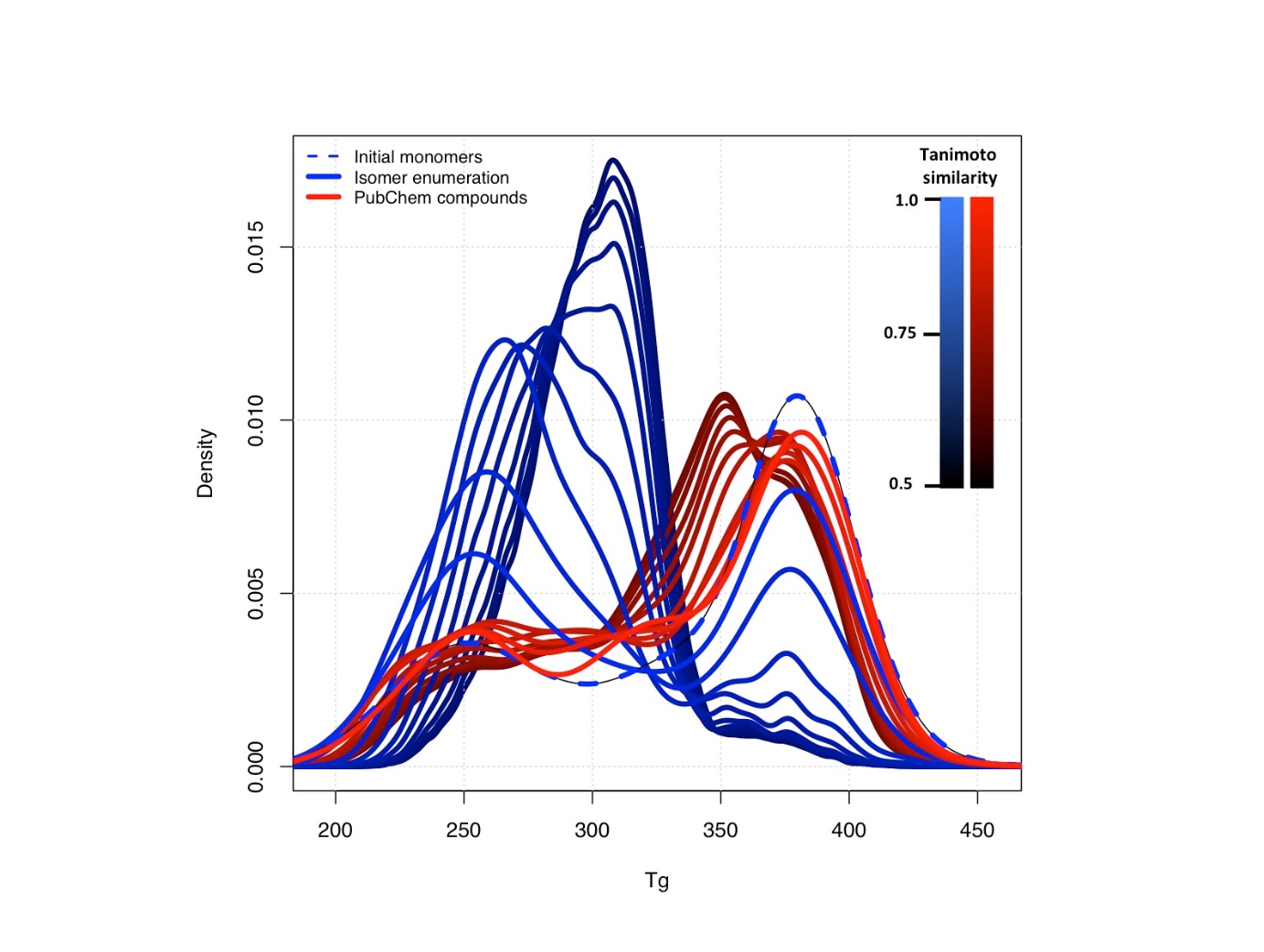


**Figure S3: Distributions of predicted T_g_ values for enumerated isomers and for isomers found in PubChem with varying Tanimoto threshold.** Distribution of predicted polymer glass transition temperature *T*_g_ for enumerated isomers and for isomers found in Pubchem of a reference set of 158 monomers with a Tanimoto similarity greater than a threshold varying between 0.5 and 1.
